# Systematic Identification of Cyclic-di-GMP Binding Proteins in *Vibrio cholerae* Reveals a Novel Class of Cyclic-di-GMP-Binding ATPases Associated with Type II Secretion Systems

**DOI:** 10.1371/journal.ppat.1005232

**Published:** 2015-10-27

**Authors:** Kevin G. Roelofs, Christopher J. Jones, Sarah R. Helman, Xiaoran Shang, Mona W. Orr, Jonathan R. Goodson, Michael Y. Galperin, Fitnat H. Yildiz, Vincent T. Lee

**Affiliations:** 1 Department of Cell Biology and Molecular Genetics, University of Maryland at College Park, Maryland, United States of America; 2 Department of Microbiology and Environmental Toxicology, University of California, Santa Cruz, Santa Cruz, California, United States of America; 3 National Center for Biotechnology Information, National Library of Medicine, National Institutes of Health, Bethesda, Maryland, United States of America; University of Washington School of Medicine, UNITED STATES

## Abstract

Cyclic-di-GMP (c-di-GMP) is a ubiquitous bacterial signaling molecule that regulates a variety of complex processes through a diverse set of c-di-GMP receptor proteins. We have utilized a systematic approach to identify c-di-GMP receptors from the pathogen *Vibrio cholerae* using the Differential Radial Capillary Action of Ligand Assay (DRaCALA). The DRaCALA screen identified a majority of known c-di-GMP binding proteins in *V*. *cholerae* and revealed a novel c-di-GMP binding protein, MshE (VC0405), an ATPase associated with the mannose sensitive hemagglutinin (MSHA) type IV pilus. The known c-di-GMP binding proteins identified by DRaCALA include diguanylate cyclases, phosphodiesterases, PilZ domain proteins and transcription factors VpsT and VpsR, indicating that the DRaCALA-based screen of open reading frame libraries is a feasible approach to uncover novel receptors of small molecule ligands. Since MshE lacks the canonical c-di-GMP-binding motifs, a truncation analysis was utilized to locate the c-di-GMP binding activity to the N-terminal T2SSE_N domain. Alignment of MshE homologs revealed candidate conserved residues responsible for c-di-GMP binding. Site-directed mutagenesis of these candidate residues revealed that the Arg9 residue is required for c-di-GMP binding. The ability of c-di-GMP binding to MshE to regulate MSHA dependent processes was evaluated. The R9A allele, in contrast to the wild type MshE, was unable to complement the Δ*mshE* mutant for the production of extracellular MshA to the cell surface, reduction in flagella swimming motility, attachment to surfaces and formation of biofilms. Testing homologs of MshE for binding to c-di-GMP identified the type II secretion ATPase of *Pseudomonas aeruginosa* (PA14_29490) as a c-di-GMP receptor, indicating that type II secretion and type IV pili are both regulated by c-di-GMP.

## Author Summary

Cyclic-di-GMP (c-di-GMP) is a ubiquitous bacterial signaling molecule that regulates important bacterial functions, including virulence, antibiotic resistance, biofilm formation and cell division. The list of known c-di-GMP receptors is clearly incomplete. Here we utilized a systematic and unbiased biochemical approach to identify c-di-GMP receptors from the 3,812 genes of the *Vibrio cholerae* genome. Results from this analysis identified most known c-di-GMP receptors as well as MshE, a protein not known to interact with c-di-GMP. The c-di-GMP binding site was identified at the N-terminus of MshE and requires a conserved arginine residue in the 9^th^ position. MshE is the ATPase that powers the secretion of the MshA pili onto the surface of the bacteria. We show that c-di-GMP binding to MshE is required for MshA export and the function of the pili in attachment and biofilm formation. ATPases responsible for related processes such as type IV pili and type II secretion were also tested for c-di-GMP binding, which identified the *P*. *aeruginosa* ATPase PA14_29490 as another c-di-GMP binding protein. These findings reveal a new class of c-di-GMP receptor and raise the possibility that c-di-GMP regulate membrane complexes through direct interaction with related type II secretion and type IV pili ATPases.

## Introduction

Cyclic diguanosine monophosphate (c-di-GMP) is a ubiquitous bacterial nucleotide secondary signaling molecule that regulates cellular processes in response to environmental and cellular stimuli. The elements of this canonical pathway of signal production, signal transduction, altered activity and signal removal were elegantly described by the Benziman lab over twenty-five years ago [1, 2]. C-di-GMP is synthesized by diguanylate cyclases (DGCs) via a catalytic GGDEF domain [1, 3, 4]. Once made in the cell, c-di-GMP binds to macromolecule receptors to allosterically alter their activities. C-di-GMP signaling is terminated through hydrolysis by phosphodiesterases (PDEs) that contain catalytic EAL or HD-GYP domains [5–8]. In the characterization of bacterial cellulose synthase in *Komagataeibacter xylinus*, the Benziman lab demonstrated the importance of c-di-GMP in the allosteric activation of the cellulose synthase complex [1, 9, 10]. Recent structure elucidation of the BcsA-BcsB-c-di-GMP complex validated these early finding and provided a molecular mechanism for c-di-GMP activation of cellulose biosynthesis [11]. Since this initial description of c-di-GMP regulation of cellulose biosynthesis, genome sequencing has revealed genes for DGCs in diverse bacteria indicating that c-di-GMP is a ubiquitous and important signaling molecule in prokaryotes that regulates a variety of phenotypes [12].

The identification of receptor proteins for c-di-GMP is needed for understanding the regulation by this ubiquitous signaling molecule. However, the process of identifying c-di-GMP binding proteins has been challenging for several reasons. First, c-di-GMP simultaneously regulates complex traits including promoting biofilm formation, inhibiting motility and additional pathways [13–15] indicating that there are likely many c-di-GMP receptors in the cell. Second, although there are several defined protein domains that bind c-di-GMP (see below), these domains do not accurately predict c-di-GMP binding proteins. For example, the PilZ domain binds c-di-GMP [16], but the PilZ protein in *P*. *aeruginosa*, for which the domain is named, does not bind c-di-GMP [17]. In *V*. *cholerae*, there are five PilZ domain proteins, but these five proteins do not fully explain all of the observed c-di-GMP regulated effects [18]. Third, c-di-GMP binds a number of proteins that do not have predicted binding motifs or were predicted to bind a different ligand. Examples of novel c-di-GMP binding proteins include VpsT [19], VpsR [20] and FlrA [21] in *V*. *cholerae* as well as FleQ in *P*. *aeruginosa* [22]. In addition, the Clp protein in *Xanthomoas* species, a homolog of the cAMP receptor protein (CRP) of *E*. *coli*, binds c-di-GMP rather than cAMP [23, 24]. Another example of a c-di-GMP receptor that was not predicted is the BldD of *Streptomyces coelicolor* binds two dimers of c-di-GMP to repress transcription [25]. Together, these studies reveal the diversity of cellular targets of c-di-GMP that mediate complex regulation and the highlight the challenges in identifying the c-di-GMP-binding receptors that are responsible for c-di-GMP regulation.

Many approaches have been utilized to identify c-di-GMP binding proteins. The first proteins shown to bind c-di-GMP included the enzymes that make and degrade c-di-GMP. The I-site of DGCs binds c-di-GMP to provide product feedback inhibition of DGC activity [4, 26]. Enzymatically active or inactive PDEs are also capable of c-di-GMP binding [5, 27–30]. Bioinformatic studies revealed that the PilZ domain is a c-di-GMP binding domain [16]. In addition, targeted and unbiased approaches have been employed to identify c-di-GMP receptor proteins. In the targeted approach, genes of c-di-GMP regulated processes were tested for c-di-GMP binding [19–25, 31, 32]. These studies led to the discoveries of the c-di-GMP receptor proteins that have not been predicted by bioinformatics and have motivated identification of novel c-di-GMP receptor proteins using systematic approaches. Affinity pull-down assays using c-di-GMP conjugated sepharose resin, biotin, or a tripartite c-di-GMP capture compound enriched c-di-GMP binding proteins from whole cell lysates, which were subsequently identified by mass spectrometry [33–35]. This approach has also been employed to identify binding proteins of another prokaryotic cyclic dinucleotide, cyclic-di-AMP (c-di-AMP) [36–38]. An alternative unbiased approach utilizes the Differential Radial Capillary Action of Ligand Assay (DRaCALA) to systematically screen protein expression libraries for ligand binding activity. DRaCALA relies on the differential spreading of bound and unbound radiolabeled ligand when mixed with protein and spotted on a nitrocellulose membrane [39]. In addition, DRaCALA allowed direct detection of c-di-GMP receptors expressed in *E*. *coli* whole cell lysates thus enabling the screening of individual genes from a target genome [39]. This approach has been used on *Staphylococcus aureus* and *Escherichia coli* open reading frame (ORF) libraries to identify c-di-AMP and c-di-GMP binding proteins, respectively [36, 40].

*Vibrio cholerae* was chosen as an organism for a DRaCALA based screen of c-di-GMP binding proteins since an open reading frame library was available [41] and it is an organisms that extensively utilize c-di-GMP signaling system to regulate motility, biofilm formation, pathogenesis, and survival upon dissemination to environmental reservoirs [42–46]. The *V*. *cholerae* O1 El Tor N16961 genome encodes 62 proteins with domains for c-di-GMP metabolism including 31 GGDEF, 13 EAL, 9 GGDEF+EAL, and 8 HD-GYP domain proteins [47–49]. However, only 5 c-di-GMP receptor proteins have been identified, including 2 PilZ domain proteins PlzC and PlzD [18], and 3 transcription factors VpsT, VpsR, and FlrA [19–21]. From the DRaCALA screen of the *V*. *cholerae* open reading library, a number of predicted c-di-GMP binding proteins were identified. In addition, MshE, an ATPase in the mannose sensitive hemagglutinin (MSHA) type IV pilus operon, was also revealed as a c-di-GMP receptor. Purified MshE specifically binds c-di-GMP with a high affinity (K_d_ approximately 2 μM). Screening of related type II secretion and type IV pili ATPases identified a gene in *P*. *aeruginosa*, *PA14_29490*, as a c-di-GMP receptor. Through fragmentation of MshE and site-directed mutagenesis of the conserved residues, the arginine at position 9 was identified as a residue required for c-di-GMP binding. Complementation of Δ*mshE* mutants with wild type *mshE* restored c-di-GMP regulation of motility, pilus production, and biofilm formation. In contrast, complementation with *mshE R9A* failed to restore c-di-GMP regulation of MshA pilus function. These results define a novel set of c-di-GMP-binding ATPases associated with type IV pili and type II secretion systems and demonstrate the utility of a DRaCALA screen for identification of c-di-GMP receptor proteins.

## Results

### DRaCALA screening of a *V*. *cholerae* ORFeome for c-di-GMP binding activity

We sought to systematically identify protein receptors of c-di-GMP using DRaCALA by individually testing *Vibrio cholerae* ORFs expressed in *E*. *coli* whole cell lysates for ^32^P-c-di-GMP binding activity. The 3,812 unique ORFs from the *V*. *cholerae* O1 El Tor N16961 ORFeome pDONR plasmids were recombined into gateway-compatible histidine (His-ORF) or His-maltose binding protein (His-MBP-ORF) expression vectors in a single Gateway reaction [50] and selected on agar plates containing either carbenicillin or gentamicin, respectively. For each ORF, multiple transformants were inoculated into a single well of a 96-well microtiter plate to create His-ORF and His-MBP-ORF libraries from which whole cell lysates were generated. Protein expression in whole cell lysates was tested for 348 ORFs by PAGE separation and revealed by staining with Coomassie. A band corresponding to the predicted molecular weight was visualized for 49% of His-ORF and 76% of His-MBP-ORF fusions for a combined coverage of 81% of the *V*. *cholerae* ORFeome. These results indicate that most *V*. *cholerae* proteins are overexpressed in the His-ORF and His-MBP-ORF libraries, thus enabling a systematic genome-wide DRaCALA screen for c-di-GMP binding proteins.

**Fig 1:**
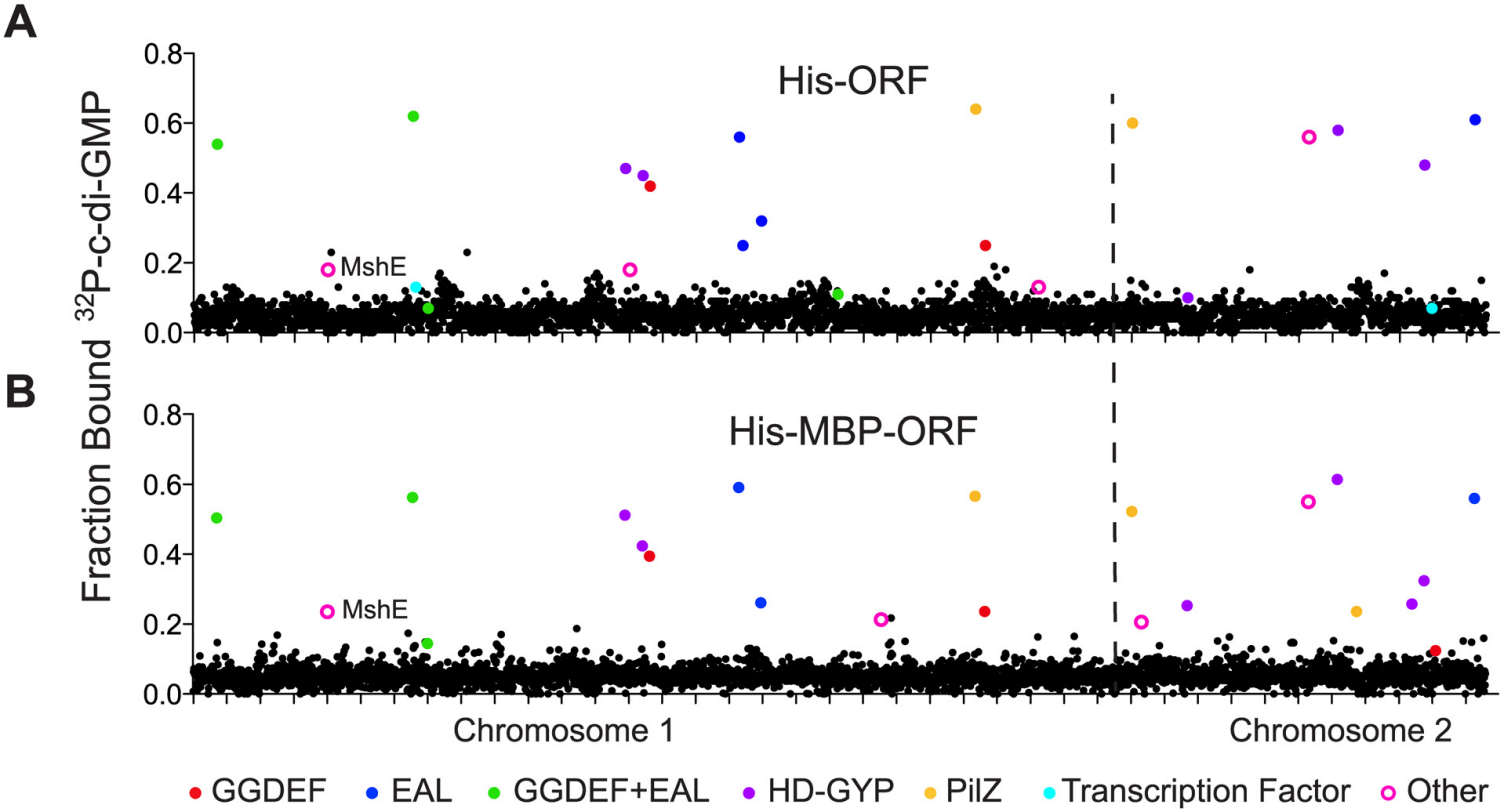
Primary DRaCALA screen of *Vibrio cholerae* ORF libraries. Average fraction bound ^32^P-c-di-GMP vs. individual *V*. *cholerae* (A) His-ORFs and (B) His-MBP-ORFs overexpressed in *E*. *coli* whole cell lysates. ORFs are arranged by VC gene number along the X-axis. ORFs validated as positive in the secondary screen are indicated by color and classified by type of c-di-GMP-binding protein. “Other” refers to proteins that have not been predicted or demonstrated to bind c-di-GMP.

Whole cell lysates from His-ORF and His-MBP-ORF libraries were tested for c-di-GMP binding by DRaCALA using a 96 well pin tool. The fraction bound of ^32^P-c-di-GMP was measured in duplicate for each whole cell lysate and positive *V*. *cholerae* ORFs were defined as those having c-di-GMP fraction bound three standard deviations above the mean for both measurements (Fig 1, S1 Table) (see Material and Methods). The positive control expressing PelD, a known c-di-GMP-binding protein, was above the cutoff in each 96-well plate. This primary screen identified 55 His-ORF and 47 His-MBP-ORF proteins that significantly increased the fraction bound of ^32^P-c-di-GMP. A secondary screen was performed to validate these ORFs. In total, 23 His-ORFs and 22 His-MBP-ORFs (28 unique ORFs total) were validated as positive for c-di-GMP binding (Fig 1, Table 1). The specificity for c-di-GMP binding of cell lysates expressing positive ORFs was determined by competition experiments using unlabeled guanosine nucleotides (Table 1). Unlabeled c-di-GMP significantly reduced ^32^P-c-di-GMP binding for ORFs listed in Table 1. In contrast, unlabeled GTP and cGMP did not reduce ^32^P-c-di-GMP binding, suggesting that the measured binding activity was specific for c-di-GMP. Together these results illustrate how sequential high-throughput DRaCALA screens can identify genetic elements that encode proteins with specific ligand binding activity. This screen of the *V*. *cholerae* ORFeome for c-di-GMP binding proteins identified known and candidate c-di-GMP receptors.

**Table 1:** Specific binding of validated ORFs to c-di-GMP.

	His-ORF				His-MBP-ORF				Protein (c-di-GMP binding domain)	Reference for c-di-GMP Binding and Activity^1^
VC Gene #	NC	c-di-GMP	cGMP	GTP	NC	c-di-GMP	cGMP	GTP		
VC0405	0.29	0.07*	0.30	0.39	0.36	0.03 *	0.32	0.38	MshE	N/A
VC1308	0.34	0.06*	0.26	0.35	-	-	-	-	TyrR	N/A
VC2066	-	-	-	-	0.12	0.02 *	0.08	0.12	FliA	N/A
VC2529	0.08	0.01*	0.07	0.09	-	-	-	-	RpoN	N/A
VCA0071	-	-	-	-	0.05	0.02	0.05	0.05	PstC	N/A
VCA0593	0.55	0.02 *	0.62	0.61	0.53	0.03 *	0.59	0.57	N/A	N/A
VC0665	0.15	0.02 *	0.15	0.14	-	-	-	-	VpsR	(+) [20]
VCA0952	0.17	0.04 *	0.18	0.24	-	-	-	-	VpsT	(+) [19]
VCA0042	0.58	0.03 *	0.65	0.61	0.48	0.07 *	0.50	0.52	PlzD (PilZ)	(+) [18]
VC2344	0.57	0.04 *	0.61	0.62	0.53	0.03 *	0.55	0.55	PlzC (PilZ)	(+) [18]
VCA0735	-	-	-	-	0.28	0.03 *	0.27	0.31	PlzE (PilZ)	(+) [18]
VC1370	0.45	0.03 *	0.51	0.47	0.30	0.02 *	0.34	0.37	(GGDEF)	N/A
VC2370	0.16	0.04 *	0.20	0.15	0.07	0.04	0.13	0.08	(GGDEF)	N/A
VCA0965	-	-	-	-	0.17	0.02 *	0.14	0.13	CdgF (GGDEF)	(+) DGC [109]
VC0658	0.55	0.02 *	0.60	0.61	0.56	0.02 *	0.58	0.59	(GGDEF + EAL)	N/A
VC0072	0.36	0.00 *	0.49	0.48	0.47	0.02 *	0.51	0.55	(GGDEF + EAL)	N/A
VC0703	0.19	0.05 *	0.19	0.23	0.23	0.01 *	0.19	0.20	MbaA (GGDEF + EAL)	N/A
VC1934	0.24	0.02 *	0.21	0.22	-	-	-	-	(GGDEF + EAL)	N/A
VC1641	0.58	0.02 *	0.66	0.63	0.30	0.04 *	0.32	0.36	(EAL)	N/A
VC1710	0.42	0.06 *	0.50	0.58	0.42	0.02 *	0.47	0.56	(EAL)	N/A
VCA1083	0.57	0.04 *	0.64	0.61	0.53	0.04 *	0.53	0.58	(EAL)	N/A
VC1652	0.36	0.08 *	0.47	0.52	-	-	-	-	VieA (EAL)	(+) PDE [110]
VCA0681	0.51	0.01 *	0.50	0.58	0.50	0.01 *	0.48	0.55	(HD-GYP)	(+) PDE [49]
VCA0210	0.17	0.03 *	0.17	0.15	0.33	0.04 *	0.41	0.34	(HD-GYP)	N/A
VC1295	0.46	0.02 *	0.55	0.52	0.51	0.02 *	0.55	0.57	(HD-GYP)	N/A
VCA0931	0.33	0.01 *	0.36	0.42	0.36	0.02 *	0.41	0.48	(HD-GYP)	N/A
VC1348	0.49	0.02 *	0.55	0.52	0.54	0.02 *	0.54	0.56	(HD-GYP)	N/A
VCA0895	-	-	-	-	0.28	0.01 *	0.27	0.26	(HD-GYP)	N/A

^1^ Binding and enzymatic activity is deemed positive if activity has been demonstrated for the purified protein or if mutation of residues required for c-di-GMP binding or enyzmatic activity regulate a c-di-GMP dependent phenotype.

(+) = c-di-GMP binding observed,

DGC = diguanylate cyclase activity observed,

PDE = phosphodiesterase activity observed.

Two-way ANOVA was used to determine if the fraction bound to ^32^P-c-di-GMP in the presence unlabeled c-di-GMP competitor differed significantly from no competitor (NC).

* = p < 0.001.

### Positive ORFs encode c-di-GMP binding proteins

The ORFs identified in our DRaCALA-based ORFeome screen included several proteins with either predicted or demonstrated c-di-GMP binding activity. The list of 28 positive ORFs include three with GGDEF domains, four with EAL, four with both GGDEF and EAL, six with HD-GYP, three with PilZ, two encoding known c-di-GMP binding transcription factors, and six proteins lacking a defined c-di-GMP binding domain (Table 1). Of the proteins containing only the GGDEF domain, three ORFs identified contain the RxxD motif required for c-di-GMP binding (VC1370, VC2370, VCA0965 (CdgF)), which is present in only 11 of the 37 ORFs with GGDEF domains (S1 Fig). Four ORFs (VC0072, VC0658, VC0703 (MbaA), VC1934) were identified with both GGDEF and EAL domains, but lacking the RxxD motif, suggesting that c-di-GMP binding occurs at the EAL domain. Both EAL and HD-GYP domains can bind c-di-GMP as a substrate for hydrolysis. The screen identified four ORFs containing an EAL domain (VC1641, VC1652 (VieA), VC1710, VCA1083) and six ORFs containing a HD-GYP domain (VC1295, VC1348, VCA0210, VCA0681, VCA0895, VCA0931). The *V*. *cholerae* genome contains 5 ORFs that encode PilZ domains. While four of these (PlzA, -C, -D, and -E) retain RxxxR and DxSxxG motifs required for c-di-GMP binding, only PlzC (VC2344) and PlzD (VCA0042) have been demonstrated to bind c-di-GMP biochemically [18, 51]. From the DRaCALA-based ORFeome screen, PlzC and PlzD were identified in both His-ORF and His-MBP-ORF libraries, while PlzE (VCA0735) was identified only in the His-MBP-ORF library. Previous work demonstrated c-di-GMP binding for His-fusions of PlzC and PlzD but not PlzE, suggesting that a MBP fusion to PlzE may be required for proper folding of the c-di-GMP binding site during heterologous expression of PlzE [52, 53]. Finally, two of three c-di-GMP binding transcription factors, VpsT (VCA0952) [19] and VpsR (VC0665) [20] were identified, but not FlrA (VC2137) [21]. In total, DRaCALA identified 22 of 46 (48%) proteins predicted to bind c-di-GMP and 6 of 11 (55%) proteins previously shown to bind c-di-GMP (Table 2). These results demonstrate that DRaCALA-based screen can identify all known categories of c-di-GMP binding proteins and represents an unbiased approach to discovering receptor proteins of signaling molecules.

**Table 2:** Hit rate of *V*. *cholerae* ORFs encoding predicted and previously demonstrated cdiGMP-binding proteins.^1^

C-di-GMP binding domain	Gene number	c-di-GMP binding	DRaCALA screen
GGDEF (RxxD)^2^			
	VC0900 (CdgG)	Predicted	Negative
	VC1104	Predicted	Negative
	VC1185	Predicted	Negative
	VC1216	Predicted	Negative
	VC1370	Predicted	Positive
	VC1593	Predicted	Negative
	VC2370	Predicted	Positive
	VCA0217	Predicted	Negative
	VCA0697 (CdgD)	Predicted	Negative
	VCA0960	Predicted	Negative
	VCA0965 (CdgF)	Predicted	Positive
	Total	11	3 (27%)
PilZ (RxxxR…DxSxxG)^3^			
	VC0697 (PlzA)	Predicted	Negative
	VC2344 (PlzC)	Experimentally Demonstrated [18]	Positive
	VCA0042 (PlzD)	Experimentally Demonstrated [18]	Positive
	VCA0735 (PlzE)	Predicted	Positive
	Total	4	3 (75%)
EAL^4^			
	VC0072	Predicted	Positive
	VC0130 (CdpA)	Experimentally Demonstrated [111]	Negative
	VC0137 (CdgJ)	Experimentally Demonstrated [112]	Negative
	VC0653	Predicted	Negative
	VC0658	Predicted	Positive
	VC0703 (MbaA)	Predicted	Positive
	VC1086	Experimentally Demonstrated [113]	Negative
	VC1211	Predicted	Negative
	VC1592 (AcgA)	Experimentally Demonstrated [114]	Negative
	VC1641	Predicted	Positive
	VC1652 (VieA)	Experimentally Demonstrated [42]	Positive
	VC1710	Predicted	Positive
	VC1934	Predicted	Positive
	VC2750	Predicted	Negative
	VC1851	Predicted	Negative
	VCA0080	Predicted	Negative
	VCA0101	Predicted	Negative
	VCA1083	Predicted	Positive
	VCA0785 (CdgC)	Predicted	Negative
	Total	20	8 (40%)
HD-GYP^5^			
	VC1295	Predicted	Positive
	VC1348	Predicted	Positive
	VC2340	Predicted	Negative
	VC2497	Predicted	Negative
	VCA0210	Predicted	Positive
	VCA0681	Experimentally Demonstrated [49]	Positive
	VCA0895	Predicted	Positive
	VCA0931	Predicted	Positive
	Total	8	6 (75%)
Transcription Factors			
	VC0065 (VpsR)	Experimentally Demonstrated [20]	Positive
	VC2137 (FlrA)	Experimentally Demonstrated [21]	Negative
	VCA0952 (VpsT)	Experimentally Demonstrated [19]	Positive
	Total	3	2 (67%)
**Total**			
	Overall	46	22 (48%)
	Experimental demonstrated	11	6 (55%)

^1^ Only genes represented in the *V*. *cholerae* ORF library are shown.

^2^ GGDEF containing the RxxD I-site was determined by sequence alignment (S1 Fig) [27].

^3^ PilZ domains containing the RxxxR…DxSxxG were determined by sequence alignment [16][38]. PlzB is omitted since it lacks the RxxxR…DxSxxG motifs.

^4^ EAL include all proteins containing the EAL domain (PF00563).

^5^ HD-GYP is defined as described in [60].

Six ORFs were identified which encode proteins that are not known or predicted to bind c-di-GMP, namely VC0405 (MshE), VC1308 (TyrR), VC2066 (FliA), VC2529 (RpoN), VCA0071 (PstC), and VCA0593 (Table 1). These ORFs represent potentially novel types of c-di-GMP binding proteins. To determine if these proteins bind c-di-GMP directly, His-MBP-ORF fusions were purified and assayed for c-di-GMP binding activity by DRaCALA. C-di-GMP binding was detected for purified MshE, but not for RpoN, FliA, TyrR, or VCA0593 (S2 Fig). We were unable to purify PstC. These results suggest that heterologous expression of RpoN, FliA, TyrR, or VCA0593 can induce the expression of c-di-GMP binding proteins encoded within the *E*. *coli* genome. In the remainder of the manuscript, we characterize the c-di-GMP binding properties of MshE.

### MshE specifically binds c-di-GMP with high affinity

**Fig 2:**
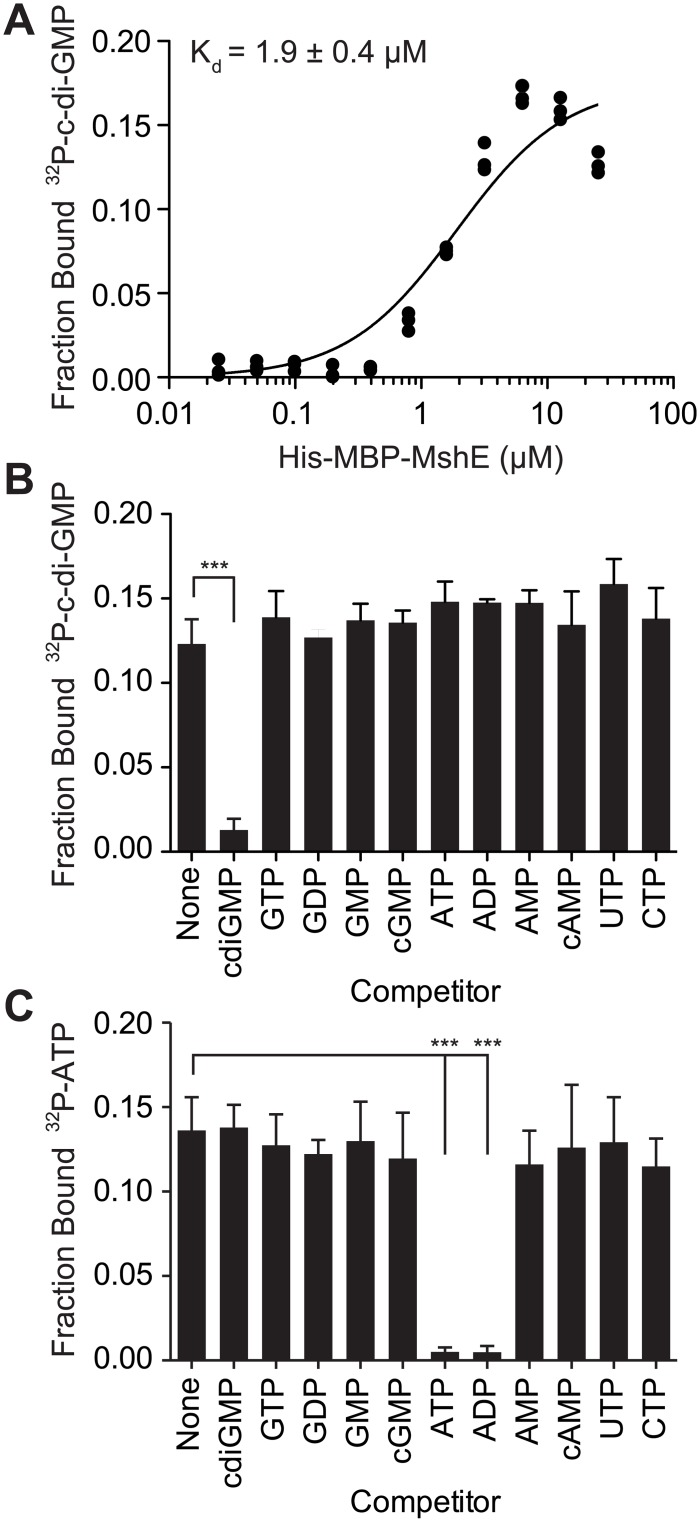
C-di-GMP bind to MshE ATPase with high affinity, specificity and independently of ATP. (A) Fraction bound ^32^P-c-di-GMP to decreasing concentrations of purified His-MBP-MshE. The dissociation constant (K_d_) is indicated. Fraction bound of His-MBP-MshE to (B) ^32^P-c-di-GMP and (C) ^32^P-ATP in the presence of 100 μM nucleotide competitors. P-value was determined by two-tailed t-test (*** p≤0.001). All data are average of three independent assays and standard deviation is indicated by error bars.

To determine the affinity and specificity of c-di-GMP-binding to MshE, purified His-MBP-MshE was assayed for binding to ^32^P-c-di-GMP by DRaCALA. The affinity of c-di-GMP-binding was determined by quantifying the fraction bound of ^32^P-c-di-GMP in serial dilutions of His-MBP MshE (Fig 2A). Non-linear regression analysis of c-di-GMP-binding vs. protein concentration using a one site-binding model estimated the dissociation constant (K_d_) for c-di-GMP to be 1.9 ± 0.4 μM. To determine the specificity of c-di-GMP-binding to MshE, we measured the fraction bound of ^32^P-c-di-GMP in the presence of unlabeled nucleotide competitors. ^32^P-c-di-GMP-binding to His-MBP-MshE was significantly decreased by unlabeled c-di-GMP, but not by GTP, GDP, GMP cGMP, ATP, ADP, AMP, cAMP, CTP, or UTP (Fig 2B). MshE also binds ATP specifically since only ATP and ADP compete for ^32^P-ATP binding (Fig 2C). These results indicate that MshE specifically binds c-di-GMP with micromolar affinity and at a site that is distinct from the ATP binding site.

### A subset of ATPases that regulate type IV pili and type II secretion systems bind c-di-GMP

**Fig 3:**
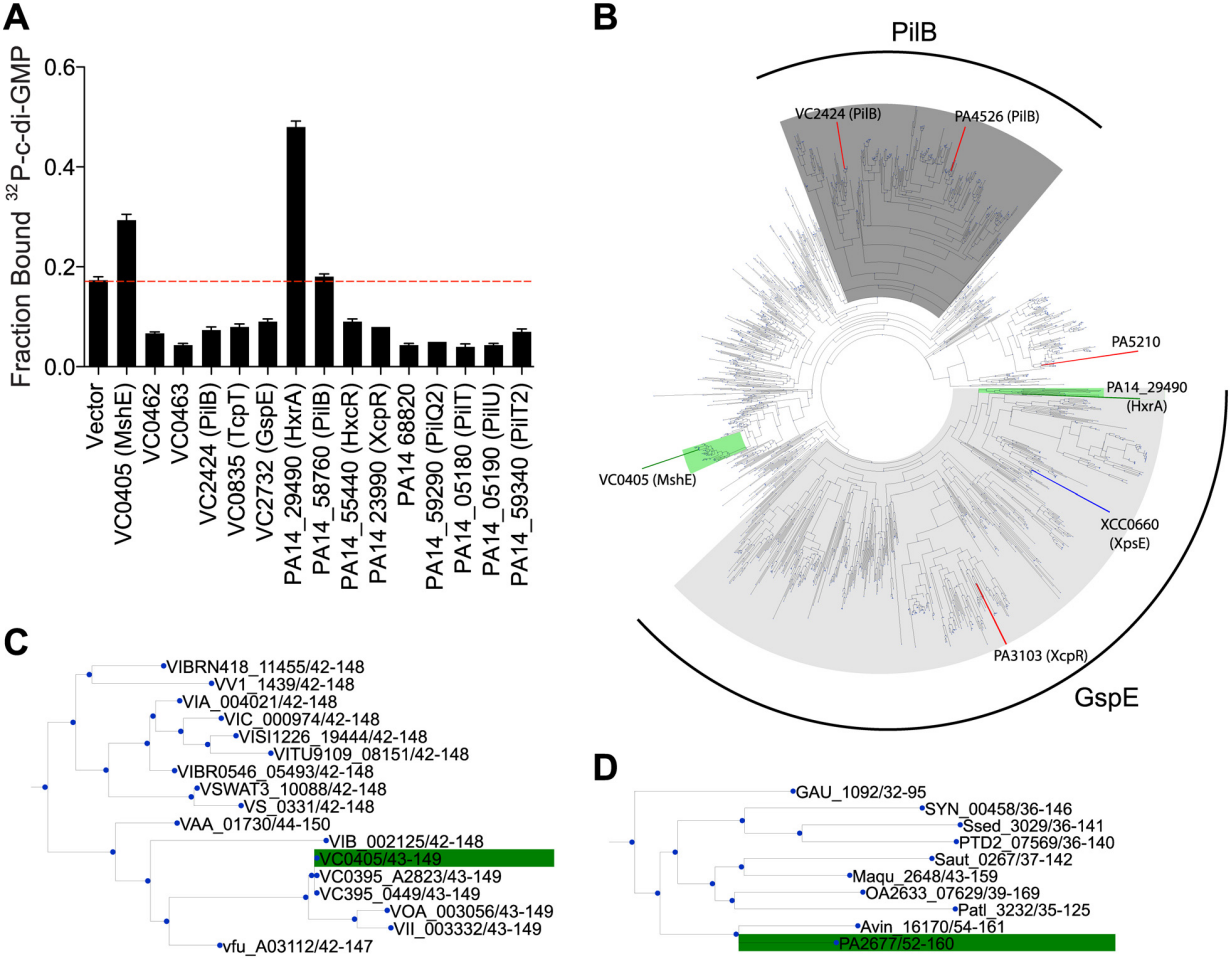
C-di-GMP binds to *V*. *cholerae* and *P*. *aeruginosa* homologs of MshE. (A) Average fraction bound ^32^P-c-di-GMP of *E*. *coli* whole cell lysate expressing *Vibrio cholerae* and *Pseudomonas aeruginosa* homologs of MshE. The dashed red line indicates background binding for a vector control strain. All data are average of three independent assays and standard deviation is indicated by error bars. (B) Unrooted phylogenetic tree of the T2SSE_N domain. Protein sequences present in the tree corresponding to proteins analyzed for c-di-GMP binding are highlighted in green (binds c-di-GMP), red (does not bind), or blue (candidate binding protein). The dark grey background corresponds to primarily type IV pili PilB sequences, and the light grey background corresponds to type II secretion protein E ATPase sequences. (C, D) Sub-trees containing VC0405 or PA14_29490 and closely related proteins.

MshE belongs to a family of ATPases associated with the biosynthesis and retraction of type IV pili and secretion by type II secretion systems. These phylogenetically related ATPases are called as T2SSE ATPases based on the type II secretion system (T2SS) nomenclature for General secretion protein E (GspE) [54, 55]. To determine if c-di-GMP-binding is a conserved feature of T2SSE ATPases, we identified homologs of MshE and assayed them for c-di-GMP-binding. Protein-Blast search with the full-length MshE amino acid sequence against the complete protein sets of *V*. *cholerae* O1 El Tor N16961 and *P*. *aeruginosa* PA14 identified five additional ATPases in *V*. *cholerae* and nine in *P*. *aeruginosa* with an E value < 1x10^-15^. These ATPases include those required for type IV pili function: PilB (VC2424 and PA14_58750,) PilT (PA14_05180 and PA14_59340), PilU (PA14_05190), and TcpT (VC0835); and type II secretion: GspE (VC2732), XcpR (PA14_23990), HxcR (PA14_55440), and HxrA (PA14_29490) [56–63]. We constructed His-ORF fusions for each *V*. *cholerae* and *P*. *aeruginosa* T2SSE ATPase and assayed c-di-GMP-binding by DRaCALA in *E*. *coli* whole cell lysate (Fig 3A). Expression of *V*. *cholerae* MshE and *P*. *aeruginosa* PA14_29490, but not other T2SSE ATPases, significantly increased the fraction bound of ^32^P-c-di-GMP. These data identified MshE and PA14_29490 as c-di-GMP-binding receptor proteins, the only ones among the T2SSE family in *V*. *cholerae* and *P*. *aeruginosa*.

PA14_29490 is the reciprocal best BLAST hit for MshE [55]. Based on genomic context, MshE functions within the MSHA operon [64, 65], while PA14_29490 is encoded within a putative T2SS operon [66, 67]. Sequence comparison of T2SSE-type ATPases from *V*. *cholerae* and *P*. *aeruginosa* showed that they differ substantially in protein length. MshE and PA14_29490, both 575 aa long, were ~200 aa longer than PilT ATPases VC0462, VC0463, PA14_05180, PA14_05190, PA14_58760 and PA14_59340. Other T2SSE-type ATPases, annotated as GspE- and PilB-type, ranged in length from 469 to 562 aa, and only PA14_68820 was the same length as MshE. All these enzymes had very similar C-terminal ATPase domains and differed primarily in their N-terminal parts, with longer proteins containing an additional domain, referred to as T2SSE_N (PF05157) domain in the Pfam database [48]. A phylogenetic tree of the N-terminal fragments of T2SSE_N-containing ATPases showed that MshE is located in the branch related to PilB ATPases responsible for type IV pili, whereas PA14_29490 is located in the branch related to GspE ATPases, which participate in type II secretion (Fig 3B). The canonical PilB and GspE ATPases that were tested (VC2424, VC2732 and their counterparts in *P*. *aeruginosa*) belong to branches of the tree that are distinct from those including MshE and PA14_29490. Expansion of the branches containing MshE (Fig 3C) and PA14_29490 (Fig 3D) reveal many homologous proteins that may also be receptors of c-di-GMP. Together, these results suggest that a subset of T2SSE ATPases represented by MshE and PA14_29490 are c-di-GMP-binding proteins.

### PA14_29490 and MshE bind c-di-GMP specifically in the N-terminal domain

**Fig 4:**
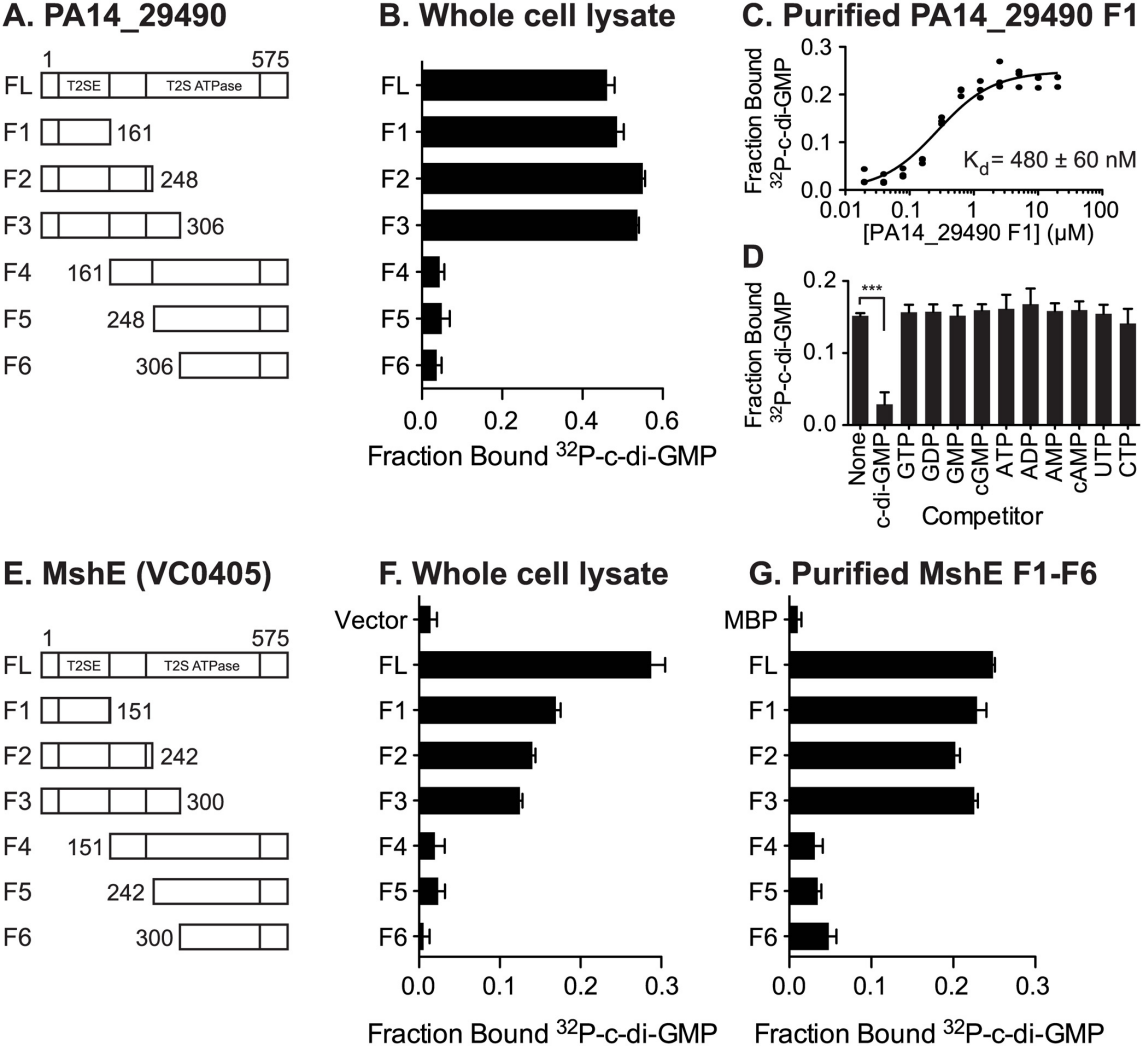
C-di-GMP binds MshE and PA14_29490 in the N-terminal T2SSE_N domain. Schematic of the truncations generated in (A) PA14_29490 and (E) MshE. C-di-GMP binding to *E*. *coli* whole cell lysates expressing each fragment of (B) PA14_29490 and (F) MshE. (C) Fraction bound ^32^P-c-di-GMP to decreasing concentrations of purified PA14_29490 fragment 1. (D) Fraction bound of purified PA14_29490 fragment 1 to ^32^P-c-di-GMP in the presence of 100 μM nucleotide competitors. (G) Fraction bound of ^32^P-c-di-GMP to purified MshE and fragments 1–6. Indicated p-value was determined by two-tailed t-test (*** p≤0.001) by comparing the indicated samples. All data are average of three independent assays and standard deviation is indicated by error bars.

Both MshE and PA14_29490 lack known c-di-GMP binding protein sequence motifs. To locate the binding site(s) on these proteins, truncation analysis was performed on both proteins. The N-terminal T2SSE_N domain and C-terminal T2SSE ATPase domain of PA14_29490 and MshE were separated at three points that were predicted to be at the ends of secondary structural elements (Fig 4A and 4E). These fragments were expressed in *E*. *coli* and the whole cell lysates were tested for c-di-GMP binding. The N-terminal fragments (F1-F3) of both PA14_29490 and MshE bound ^32^P-c-di-GMP, while the C-terminal fragments (F4-F6) did not (Fig 4B and 4F). Purified fragment 1 of PA14_29490 binds c-di-GMP with a K_d_ of 480 ± 60 nM (Fig 4C) and this binding was competed away only with c-di-GMP (Fig 4D), indicating that binding is specific. Each of the fragments of MshE was purified and tested for binding to ^32^P-c-di-GMP. Only the purified N-terminal fragments (F1-F3) of MshE bound ^32^P-c-di-GMP, while the C-terminal fragments (F4-F6) did not (Fig 4G). These results indicate that the binding site for c-di-GMP is located in the N-terminal domain of the protein and is distinct from the ATPase domain in the C-terminus.

### MshE binding to c-di-GMP requires a specific arginine residue

**Fig 5:**
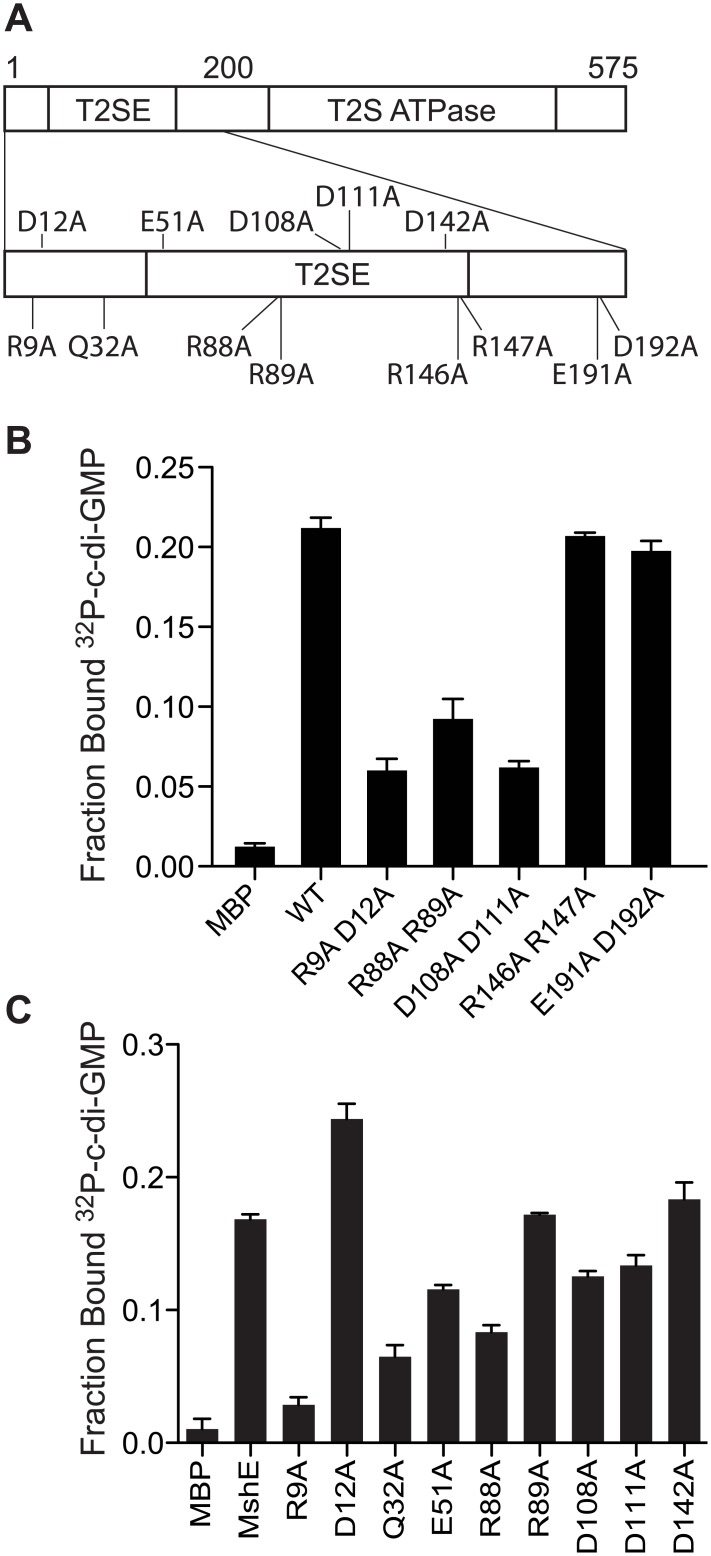
R9 is required for MshE to bind c-di-GMP. (A) Schematic of the charged and polar conserved residues in the first 200 amino acids of MshE targeted for site-directed alanine substitution. (B) Fraction bound of ^32^P-c-di-GMP to purified MshE (WT) and indicated pairs of alanine substitutions. (C) Fraction bound of ^32^P-c-di-GMP to purified MshE (WT) and indicated individual alanine substitution. All data are average of three independent assays and standard deviation is indicated by error bars.

We hypothesized that c-di-GMP regulation of MSHA is an evolutionarily conserved property. To test this idea, we identified MshE homologs from genomes containing MSHA-like operons as defined by having the *mshN* gene upstream of *mshE* and the *mshG* gene downstream of *mshE* (S3A Fig). ClustalW alignment revealed residues within the first 151 amino acids that are 100% identical among these proteins, including 4 conserved motifs: RLGDLLV, ARRxRAL, SDPADL, and DxxYRRT (S3B Fig). Since charged residues, in particular arginines, have been shown to participate in c-di-GMP binding in a variety of receptor proteins, mutant variants with alanine replacements within these 4 motifs were generated by site-directed mutagenesis including R9A/D12A, R88A/R89A, D108A/D111A, and R146A/R147A (Fig 5A). As a control, the conserved E191 and D192 residues, which are located outside of the first 151 amino acid fragment, were also changed to alanine. Purified R9A/D12A, R88A/R89A, and D108A/D111A variants were reduced for binding to c-di-GMP, whereas R146A/R147A and E191A/D192A did not affect binding to c-di-GMP (Fig 5A). These results indicate that motifs 1, 2 and 3 contribute to c-di-GMP binding, while motif 4 is dispensable. The E191A/D192A variant binds c-di-GMP similarly to the wild-type protein (Fig 5A), in agreement with the results from fragment analysis (Fig 4E). To determine the contribution of each amino acid residue within the first 3 motifs and the other conserved, charged amino acids, we generated and purified MshE variants with single alanine substitution in positions R9, D12, Q32, E51, R88, R89, D108, D111 and D142 (S4A Fig). The R9A variant had an 83% reduction in c-di-GMP binding, while Q32A and R88A variants had 61% and 50% reduction, respectively (Fig 5C). Interestingly, the R9/D12 residues represent an RxxD motif described in DGC I-site [26], PelD [31] and GIL domain of BcsE [40]. In those proteins, both residues are critical for c-di-GMP binding. In contrast, the MshE D12A variant actually binds c-di-GMP better than wild-type MshE (Fig 5C), indicating that MshE does not contain an I-site-like binding sequence. Each of these MshE variants was also tested for ATP binding. The R88A variant showed an 88% reduction in ATP binding and was the only variant that had a reduction by more than 50% (S4B Fig). Thus, the defect associated with c-di-GMP binding in R88A variant may be due to a general folding problem for this specific protein. Together, these results indicate MshE contains a novel c-di-GMP binding site that requires R9 residue with contribution from the Q32 residue.

### MshE binding to c-di-GMP enhances ATPase activity

The effect of c-di-GMP on the ATPase activity of MshE was assessed by testing the WT MshE and the R9A proteins in the presence and absence of c-di-GMP. WT MshE produced 68 μM of phosphate from ATP without c-di-GMP and increased to 75 and 76 μM with the addition of 10 and 33 μM of c-di-GMP, respectively (S5 Fig). In contrast, R9A protein produced 60, 64, and 58 μM of phosphate with addition of 0, 10, and 33 μM of c-di-GMP. These results indicate that the ATPase activity is increased for WT MshE in response to c-di-GMP at concentrations above the dissociation constant, while c-di-GMP had no effect on the R9A protein. However, the magnitude of the enhanced ATPase activity is only about 10% suggesting that c-di-GMP may have additional effects on MshE interaction with the MshA substrate or other components of the MSHA export machinery.

### C-di-GMP binding to MshE is required for MSHA function in attachment and biofilm formation

**Fig 6:**
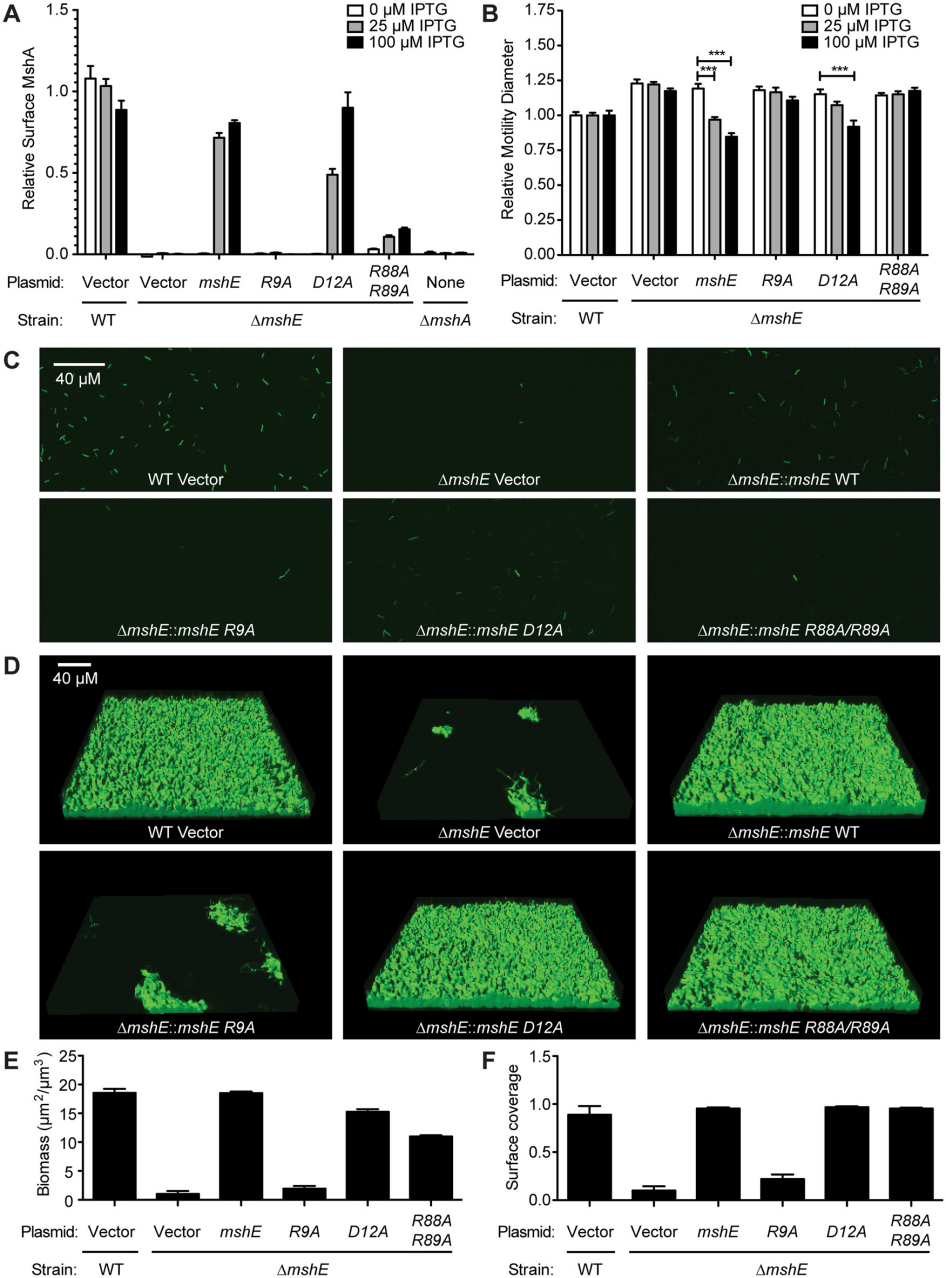
MshE interaction with c-di-GMP is essential for MSHA function. (A) Surface MshA pilin production under varying IPTG induction determined via ELISA with MshA-specific antibody. Two biological replicates were assayed in duplicate and normalized to the average of the WT strain with vector. (B) Expression of *mshE* reduces motility in soft agar. The diameters of migration zones were measured after 16 hours of growth at 30°C with or without IPTG induction and normalized to the motility of the WT strain harboring the empty vector. Three biological replicates were performed in duplicate. Induced strains were compared to uninduced strains with ANOVA followed by Bonferroni Multiple Comparison test. (** p≤0.01, *** p≤0.001). (C) Attachment phenotypes observed with confocal microscopy 4 h post inoculation in a flow cell system. Scale bar represents 40μm. (D) Three-dimensional biofilm structures observed with confocal microscopy 24 h post inoculation in a flow cell system. Scale bar represents 40μm. (E,F) COMSTAT quantitative analysis of biomass and surface coverage of biofilms from (D). Three images from each of two independent experiments were analyzed.

MSHA is a responsible for initial attachment of *V*. *cholerae* to surfaces and subsequent biofilm formation [68]. Recently, MSHA was demonstrated to reduce flagella mediated swimming motility [69]. We sought to determine whether MshE is the c-di-GMP receptor regulating MSHA activity by assessing the amount of MshA pili on the bacterial surface, the effect on swimming motility, and biofilm formation. The effect of MshE on the export of MshA to the surface of *V*. *cholerae* was assessed by surface ELISA using antibodies specific to MshA (Fig 6A, WT and Δ*mshA*). The Δ*mshE* mutant is defective for MshA export (Fig 6A). This defect was complemented by wild type *mshE* and the *D12A* allele, but not the *R9A* allele (Fig 6A). Complementation with the *R88A*/*R89A* allele was able to restore the export of ~10% of MshA observed in wild type cells. A recent study revealed that *V*. *cholerae* swimming motility is reduced by interaction of MSHA with surfaces [69]. *V*. *cholerae* was assessed for motility through soft agar assay. Wild type *V*. *cholerae* had reduced swimming motility and the Δ*mshE* mutant has increased motility (Fig 6B, WT and Δ*mshE* vector) recapitulating previous observations [69]. The ability of either wild-type *mshE* or *mshE* variant under the *tac* promoter in trans on a plasmid to complement Δ*mshE* phenotypes were evaluated. Induction of either allele that binds c-di-GMP, wild-type *mshE* or the *D12A* variant, reduced motility to wild type levels (Fig 6B). Induction of the variant defective for c-di-GMP binding (*R9A*) failed to reduce motility (Fig 6B). The *R88A/R89A* allele, that had reduced binding to c-di-GMP and ATP, also failed to reduce motility. Additionally, the requirement for MshE to bind c-di-GMP on biofilm formation was assessed using a flow cell system. At 4 hours post-inoculation, wild-type was able to attach to the surface, whereas fewer Δ*mshE* mutants attached to the surface (Fig 6C). Complementation of Δ*mshE* mutants with wild type *mshE* and the *D12A* allele restored attachment, whereas complementation with either *R9A* or *R88A*/*R89A* allele did not (Fig 6C). At 24 hours post-inoculation, the initial differences in attachment were more pronounced. Wild type and Δ*mshE* mutant complemented with either *mshE* or *D12A* formed mature biofilm (Fig 6D). Complementation with the *R88A/R89A* allele also restored biofilm formation indicating a small amount of surface MshA can restore MSHA activity. In contrast, the Δ*mshE* mutant complemented with *R9A* showed small patches of biofilms similar to Δ*mshE* with the vector control (Fig 6D). Quantification of the biofilms revealed that both biomass (Fig 6E) and surface coverage (Fig 6F) are reduced for Δ*mshE* complemented with *R9A*. Together, these results indicate that the ability of MshE to bind c-di-GMP via the R9 residue is required for MshA export to the cell surface and MSHA-mediated phenotypes.

## Discussion

### Evaluation of DRaCALA ORF screens for identification of ligand binding proteins

DRaCALA screens are a relatively new method for identifying ligand binding proteins that combines arrayed protein libraries with a high-throughput biochemical assay of protein-ligand interactions. Two recent publications have successfully used DRaCALA screening to identify novel cyclic-dinucleotide protein receptors. A screen for c-di-AMP binding proteins in the *Staphylococcus aureus* strain COL ORFeome identified PstA and KdpD [36]. The screen also identified KtrA, which was the only protein identified by affinity pull-down using c-di-AMP magnetic beads in the same study [36]. The crystal structures of PstA-c-di-AMP [70–72] and KtrA-c-di-AMP complexes [73] have been solved. KdpD binding motif has been recently identified [74]. Since the DRaCALA screen, one other additional c-di-AMP receptor has been identified in Gram-positive bacteria, pyruvate carboxylase of *Listeria monocytogenes* [38]. The *S*. *aureus* pyruvate carboxylase has different residues within the binding motif than the *L*. *monocytogenes* and does not bind c-di-AMP [38]. Together these studies demonstrate that DRaCALA was able to identify three novel bona-fide c-di-AMP receptors and correctly not detect a protein that does not bind c-di-AMP. Additionally, a DRaCALA screen for c-di-GMP binding proteins in the *E*. *coli* K12 ASKA overexpression gene library revealed three clones overexpressing putative c-di-GMP binding proteins: BcsE, IlvH and RimO [40]. Of these, BscE was further characterized and shown to contain a novel c-di-GMP binding domain, which the authors named GIL [40]. In conjunction with the results from these screens, our identification of MshE as a c-di-GMP receptor important for MSHA pilus function in *V*. *cholerae* demonstrates that DRaCALA is a powerful approach for finding new small-molecule receptors across different bacterial species.

The results of these three DRaCALA screening experiments allow us to further assess the limitations of the screen. Our screen of the *V*. *cholerae* ORFeome identified many, but not all, of the expected binding proteins. These false negatives could be due to several factors. First, the enzymatic activity of these proteins may prevent detection. Phosphodiesterases active in the assay conditions could degrade the ^32^P-c-di-GMP probe prior to application on nitrocellulose, while active diguanylate cyclases could produce excess c-di-GMP to compete for binding with the subsequently added ^32^P-c-di-GMP probe. The failure to detect binding in many EAL and GGDEF domain proteins in our screen, as well as the lack of binding detected for all I-site-containing GGDEF proteins assayed in the *E*. *coli* DRaCALA screen [40], could be due to these factors. Second, poor expression of the ORF can result in a false negative because DRaCALA relies on the expression of proteins above the K_d_. In our assay of protein expression, we found that only a subset of the ORFs tested had a protein band of the correct size on SDS-PAGE. Similarly, some false negatives of c-di-GMP binding proteins in the *E*. *coli* screen were due to poor protein expression as detected by protein band intensity on SDS-PAGE [40]. Third, the DRaCALA screen interrogates binding of individually expressed ORFs within a heterologous system. Thus proteins that require endogenous binding partners or activating factors to interact with ligand may not be active. Our screen also yielded false positives, which could be due to two factors. First, the expression of proteins that can activate the production of c-di-GMP binding proteins encoded in the *E*. *coli* genome can result in a positive signal. Second, the statistical method utilized to identify “positive” fraction bound may falsely identify proteins whose fraction bounds are near the cutoff, as was likely the case for Adk, a false positive result in the *S*. *aurus* c-di-AMP screen [36]. Both these categories of false positives can be detected after re-assaying binding with purified protein.

We suggest several methods to increase the fidelity of DRaCALA-based screens: 1. Testing the ORFs with multiple fusion proteins can increase the likelihood of overexpressing recombinant proteins that retain ligand binding activity, 2. Express the ORFeome in a strain genetically modified to remove endogenous c-di-GMP signaling components and thus reduce the likelihood of false positives [75, 76], and 3. Alter the buffer used to resuspend lysates—in the case of EAL domain PDE-As, resuspension in a buffer containing Ca^2+^ rather than Mg^2+^ can inhibit the activity of PDE-As [2] and increase the likelihood of detecting proteins that degrade c-di-GMP. Although DRaCALA did not identify all c-di-GMP proteins in the *V*. *cholerae* genome, DRaCALA is an unbiased approach that allows discovery of novel receptor protein such as MshE. We believe DRaCALA-based approach can be a powerful tool for the discovery of novel receptor proteins of other small nucleotide signaling molecules.

### MshE is the founding member of a new class of c-di-GMP receptor

The MshE is a bona fide c-di-GMP binding protein was demonstrated by 1. High affinity binding with the K_d_ of 1.9 μM, 2. High specificity of binding based on competition assays, 3. A defined binding site located in the N-terminal T2SSE_N domain and 4. The requirement of the conserved R9 residue for c-di-GMP binding in vitro and for MSHA function in vivo. MshE represents a new category of c-di-GMP binding proteins since it lacks any of the previously defined c-di-GMP binding domains (DGC I-sites, EAL, HD-GYP, PilZ or GIL) and is the first T2SSE ATPase demonstrated to bind c-di-GMP. The conserved R9 and D12 residues are reminiscent of the RxxD c-di-GMP binding motif present in the I-site of DGCs or the RxGD binding motif present in GIL domains. For both DGC I-sites and GIL domains, the R and D residues are required for c-di-GMP binding. In contrast, MshE requires only the R9 residue for binding to c-di-GMP, while the D12 residue of MshE is dispensable. In addition to MshE, only one other type II secretion/type IV pili ATPase, PA14_29490 from *P*. *aeruginosa*, bound c-di-GMP. Other members of this subfamily likely will also have the ability to bind c-di-GMP, including XpsE from *X*. *campestris* (Fig 3B, blue line). In contrast, related ATPases including PilT and PilU are unlikely to be regulated by c-di-GMP since they are shorter and lack the T2SSE_N domain. Although MshE and PA14_29490 both bind c-di-GMP in the N-terminal T2SSE_N domain, their sequence conservation within this domain is quite low. Nonetheless, proteins containing the T2SSE_N domain should be investigated for their ability to interact with c-di-GMP (Fig 3B, 3C and 3D).

### Predicted structure of the c-di-GMP binding fragment of MshE

Two T2SSE_N domain of T2SSE ATPases have been structurally characterized including N-terminal fragment of *V*. *cholerae* GspE (Protein Data Bank (PDB) entry 2BH1_X) [77] and a related domain from *Xanthomonas campestris* (PDB: 2D27, 2D28) [78]. Sequence alignment the N-terminal 151-aa fragment of MshE to PA14_29490, *V*. *cholerae* GspE and *X*. *campestris* GspE by the Conserved Domain Database [79] revealed that the *V*. *cholerae* GspE lacks the R9 and Q32 residues. However, these residues were conserved in the GpsE/XpsEN domain from *X*. *campestris* (S6A Fig). Remarkably, the *X*. *campestris* XpsEN was crystallized in two different forms that reflect two distinct conformational states that differ in the position of two N-terminal helices [78], which include the R9 and Q32 residues (S6B Fig). In one of the structures (PDB: 2D27), R9 and Q32 are positioned within a reasonable distance from each other and, upon the rotation of first helix, could form a potential c-di-GMP-binding site (S6B Fig). In the other structure (PDB: 2D28), the region around Q32 proved so flexible that the exact position of Q32 could not be resolved [78], which is consistent with the ability of this residue to move around and participate in ligand binding. Such flexibility has also been observed in the binding sites of PilZ-containing c-di-GMP receptors [18, 51]. The conformational change of the two N-terminal helices of MshE upon binding c-di-GMP has the potential to alter its activity. Thus, type II secretion/type IV pili ATPases with the conserved arginine and glutamine residues corresponding to R9 and Q32 of MshE represent candidate c-di-GMP receptors (S6B Fig).

### Implications of MshE binding to c-di-GMP in the regulation MSHA during the *V*. *cholerae* infection cycle

*V*. *cholerae* cycles between environmental reservoirs and human infections in part using two type IV pili, MSHA and Tcp. MSHA contributes to the *V*. *cholerae* infection cycle by maintaining an environmental reservoir through attachment to chitin surfaces [80, 81] and tolerance to environmental hypotonic stress [82] through biofilm formation [83]. Upon entry into the next host, the preformed biofilms protect *V*. *cholerae* from lower pH [84] and bile [85]. Subsequently, *V*. *cholerae* express toxin co-regulated pili (Tcp) to promote colonization [86, 87] while concomitantly turning off MSHA [88]. The repression of MSHA within the host is critical since a strain with the *msha* promoter replaced with the constitutive P_*lac*_ promoter was outcompeted by the wild type strain in an infant mouse infection model [88]. This defect can be attributed to binding of secretory IgA directly to MSHA since these strains do not show a competitive difference in mice lacking IgA [88] indicating that repression of MSHA during infection is a necessary for immune evasion. Based on recombination-based in vivo expression technology (RIVET) studies [46], *tcpA* is transcribed while the *msh* operon is repressed early in the infection; in contrast, the expression pattern is reversed at the late stage of infection. The decrease in *msh* transcription early in the infection is likely regulated in part by the reduced levels of c-di-GMP since several DGCs are not expressed until late in the infection [46]. However, the precise mechanism for regulation of the c-di-GMP signal is subject to strain specificity [49, 89] and changes in the host microenvironment. Another mechanism to reciprocally regulate Tcp and MSHA is from the TcpJ pre-pilin peptidase. TcpJ has the unique property of processing both TcpA [90] and MshA [91], but cleavage of MshA by TcpJ leads to rapid degradation [91]. Our finding that MshE binding to c-di-GMP is required for MSHA production and function adds another mechanism to enhance the switch between Tcp and MSHA pili. Upon entering the host, the c-di-GMP levels are reduced, thus reducing both transcription of the *msh* operon and the function of existing export machinery to export MshA. In combination with the degradation of newly synthesized MshA by TcpJ, *V*. *cholerae* can quickly change from the immunogenic MSHA to the adhesive Tcp in the host. Late in the infection, this regulation is reversed allowing the bacteria exiting the host to express MSHA instead of Tcp to prepare for the environment.

### Implications of MshE on c-di-GMP regulation of type IV pili and type II secretion systems

Binding of MshE to c-di-GMP activates MSHA dependent phenotypes including 1. Production of MshA pili to the cell surface, 2. MSHA reduction of flagella motility, 3. Adherence to surfaces and biofilm formation. These studies show that all of these effects are lost in the *mshE R9A* mutant that is defective in binding to c-di-GMP. The implication of our results is that c-di-GMP binding to MshE is required for its activity in polymerizing MshA. This finding is intriguing since several type IV pili are regulated by c-di-GMP through different mechanisms. In both *Xanthomonas* and *Pseudomonas* spp., type IV pili are regulated by PilZ and FimX proteins [92–95]. However, the precise mechanisms of regulation appear to be different. In *X*. *axonopodis citri* and *X*. *campestris*, PilZ binds FimX and the PilB ATPase, a homolog of MshE, to form a tripartite regulatory complex, but these interactions are not conserved in *P*. *aeruginosa* [94, 96, 97]. In addition to regulating the PilB ATPase, c-di-GMP interacts with a second PilZ-domain protein XC_2249 to regulate interactions the PilT and PilU retraction ATPases in *X*. *campestris* [98]. These studies and our MshE results indicate that c-di-GMP regulates type IV pili ATPases through a number of different mechanisms. The ability of PA14_29490 to bind c-di-GMP indicates that there may be also complex regulation of type II secretion systems by c-di-GMP.

### Sustained sensing of c-di-GMP through multi-tiered regulation

C-di-GMP regulates major lifestyle changes in response to altered environmental cues. These changes incur a high cost in expenditure of cellular resources as well as an opportunity cost of committing to a sessile lifestyle. There is an emerging pattern in which c-di-GMP regulates the same phenotype at the transcriptional and post-translational levels. Examples of this include Pel polysaccharide synthesis in *P*. *aeruginosa* [31, 99], and now MSHA in *V*. *cholerae* [100]. The idea of regulation by c-di-GMP first to activate gene expression and later to activate protein function can be thought of as “sustained sensing”. Sustained sensing enables bacteria to repeatedly assess environmental and cellular conditions through multi-tiered regulation, and has been previously described for responses to iron availability, oxidative stress, and other signals [101, 102]. The concept of sustained signaling applied to c-di-GMP enables prediction of additional c-di-GMP receptors. In addition to known examples, there are additional c-di-GMP-regulated processes that could be regulated by sustained sensing. These include two classes: 1. Operons that are transcriptionally regulated by c-di-GMP, but lack known c-di-GMP receptor proteins and 2. Operons that encode c-di-GMP receptor proteins, but lack known c-di-GMP transcriptional regulation. Examples of the first class include a number of biosynthetic operons of extracellular polysaccharides, such Vps and Eps in *V*. *cholerae* [19, 100, 103], Psl in *P*. *aeruginosa* [22], *xagABC* in *X*. *campestris* [32, 104], and Bcam1330-Bcam1341 in *Burkholderia cenocepacia* [105]. These operons also likely encode a c-di-GMP binding protein to regulate polysaccharide biosynthesis at the post-translational level. Examples of the second class include cellulose synthesis in *E*. *coli* [40], the alginate biosynthesis operon in *P*. *aeruginosa* [17] and the large adhesin protein (Lap) of *P*. *fluorescens* [28]. Furthermore, c-di-GMP can also bind to riboswitches [106, 107] to provide another regulatory tier in sustained sensing. Therefore, based on the emerging theme of sustained sensing of c-di-GMP through multi-tiered regulation, we suggest that newly discovered processes regulated by c-di-GMP either transcriptionally or post-translationally be investigated for additional levels of c-di-GMP control.

## Materials and Methods

### Gateway destination vector construction

pVL791 Cb GW and pVL847 Gn GW were constructed as destination vectors in LR reactions (Life Technologies) for cloning VC ORFs. pVL791 Cb and pVL847 Gn are pET-19 derivatives that are carbenecillin or gentamycin resistant and produce N-terminal His- and His-MBP fusions respectively. The gateway destination cassette was amplified from pRFA and cloned in frame with the N-terminal fusions to produce the gateway adapted vectors.

### His-ORF and His-MBP-ORF expression library construction

The *V*. *cholerae* N16961 ORF library was obtained from BEI. LR Clonase reactions were performed per NEB protocol using miniprepped pDONR vectors from the *V*. *cholerea* ORFeome in combination with pVL791 Cb GW and pVL847 Gn GW destination vectors. Gateway reactions were transformed into an *E*. *coli T7IQ* strain (NEB) and recombinants were selected on LB agar plates containing either carbenecillin or gentamycin. Multiple colonies from individual transformations were inoculated in LB M9 rich media in 96-well plate format and grown overnight with shaking at 30°C. Overnight cultures were subcultured 1:50 into fresh media and grown for 4 hours at 30°C with shaking. Protein expression was induced by addition of 1 mM IPTG and cultures were grown for an additional 4 hours after induction. 1.5 mL of induced culture was centrifuged and cells were resuspended in 150 μL of c-di-GMP-binding buffer supplemented with 10 μg / mL DNAse, 250 μg / mL Lysozyme and 1 μM PMSF. 20 μL aliquots were transferred to 96-well microtiter plates and stored at -80°C.

### DRaCALA

Whole cell lysates for DRaCALA screening were prepared by freeze-thawing resuspended cells in microtiter plates a total of 3 times. After the final thaw, 20 μL of c-di-GMP-binding buffer supplemented with 16 pM ^32^P-c-di-GMP and 500 mM unlabeled GTP was added to whole cell lysate plates or purified proteins. 2 μL of this mixture was then spotted in duplicate on nitrocellulose using a 96-well pin tool. DRaCALA of purified proteins was performed with concentrations of protein and unlabeled competitor as indicated. Spots were allowed to dry completely (about 20 minutes) before exposing a phosphorimager screen and capturing with a Fujifilm FLA-7000. Photostimulated luminescence (PSL) from the inner spot and total PSL of the spot were quantitated with Fuji Image Gauge software. The fraction bound was calculated using measurements of the total area (A_outer_), the area of the inner circle (A_inner_), the total PSL intensity (I_total_), and the inner intensity (I_inner_) as described [39]. DRaCALA was also used to determine the ability of purified MshE and variants to bind c-di-GMP. Dissociation constants were estimated assuming a one-site binding model by a nonlinear regression of protein concentration and fraction bound where, Fraction bound = (Maximum possible Fraction Bound) * [protein concentration] / (Kd + [protein concentration]).

### Identification of positive *V*. *cholerae* ORFs in primary and secondary DRaCALA screens

In the primary screen of His-ORF and His-MBP-ORF libraries, mixtures of whole cell lysate and radiolabeled ^32^P-c-di-GMP were spotted twice. To identify DRaCALA spots with significantly increased fraction bound ^32^P-c-di-GMP, a positive cutoff three standard deviations above the mean fraction bound was created for each 96-well plate of whole cell lysates. Positive spots were iteratively removed from calculations of mean and standard deviation for individual plates, thereby decreasing the positive cutoff until no additional positives were identified. His-ORFs and His-MBP-ORFs for which both DRaCALA spots had positive binding were defined as positive and subjected to a secondary screen. Additionally, 8 His-ORF and 8 His-MBP-ORFs with only 1 DRaCALA spot with positive binding were also included in the secondary screen, but none of these ORFs were positive for c-di-GMP binding in the secondary screen.

For each primary positive His-ORF and His-MBP-ORF, 8 whole cell lysates were generated from individual clones that were isolated from the pooled transformants used to create the His-ORF and His-MBP-ORF libraries. Each His-ORF and His-MBP-ORF lysate was spotted twice by DRaCALA and individual lysates were compared to a set of 8 lysates generated from vector controls. Positive fraction bound ^32^P-c-di-GMP for DRaCALA spots was defined as those with at least 2 fold increase above the average fraction bound for the set of plate-matched vector controls. Additionally, each lysate was assayed by PCR to verify the size of the inserted ORF. Positive ORFs from this secondary screen displayed positive fraction bound ^32^P-c-di-GMP for both DRaCALA spots created from PCR positive lysates.

### Generation of phylogenetic tree

Protein sequences corresponding to COG2840 were obtained from the EggNOG 4.1 database. Each sequence aligned to Pfam Family PF05157 (Type II Secretion System, protein E, N-terminal domain) using the version 10 HMM with HMMer 3.1 and the subsequences corresponding to the T2SSE N-terminal domain were extracted. The remaining 1437 domain sequences were aligned using the MAFFT 7.157b E-INS-i algorithm and trimmed using TrimAl 1.4 to eliminate columns with more than 90% gaps. An unrooted phylogenetic tree was constructed using FastTree 2.1.8.

### Strains and plasmids

Strains used are listed in S2 Table. Plasmids are listed in S3 Table.

### Generation of MshE fragments and site-directed alanine substituted variants

Fragments of MshE and PA14_29490 were generated using the primers indicated in S4 Table. Site-directed alanine substitutions of MshE were generated by PCR amplification with the indicated primers, DpnI digest and transformation into *E*. *coli* DH5α. pVL791-MshER88A, pVL791-MshER89A, pVL791-MshED111A were generated using the primers indicated in S3 Table and the NEB Q5 Site-Directed Mutagenesis Kit. All constructs were verified by DNA sequencing.

### Protein expression and purification

*V*. *cholerae* MshE and variants were purified as previously described [39]. Briefly, *E*. *coli* T7Iq strains or *E*. *coli* BL21(DE3) containing expression plasmids were grown overnight, subcultured in fresh media and grown to OD_600_~1.0 when expression was induced with 1 mM IPTG. Induced bacteria were pelleted and resuspended in 10 mM Tris pH 8, 100 mM NaCl, 25 mM imidazole and frozen at -80°C until purification. Proteins were purified over a Ni-NTA column and eluted with 10 mM Tris pH 8, 100 mM NaCl, 250 mM imidazole. Purified proteins were exchanged into 10 mM Tris pH 8, 100 mM NaCl using Sephadex G25. Proteins were aliquoted, and frozen at -80°C until use.

### Plate motility assay

Motility plates consist of LB containing 0.3% agar supplemented with 20μg/mL ampicillin and 25 or 100μM IPTG where appropriate. Plates were poured and allowed to dry at room temperature for 4 h prior to inoculation. Colonies from overnight LB agar plates grown at 30°C were transferred to motility plates and incubated for 16 h at 30°C. Motility diameter was measured and normalized to the average of WT on each plate. Experiments were performed with three biological replicates in triplicate and data were analyzed with a Oneway ANOVA followed by Dunnett’s multiple comparison test.

### Confocal laser scanning microscopy (CLSM) and flow cell biofilm studies

Inoculation of flow cells was done by diluting overnight-grown cultures to an OD600 of 0.04 and injecting into a μ-Slide VI0.4 (Ibidi, Martinsried, Germany). To inoculate the flow cell surface, bacteria were allowed to adhere at room temperature for 1 h. Flow of 2% v/v LB (0.02% tryptone, 0.01% yeast extract, 1% NaCl; pH 7.5) containing 20μg/mL ampicillin and 100μM IPTG was initiated at a rate of 7.5 ml/h and continued for 24 h. Confocal images were obtained on a Zeiss LSM 5 PASCAL Laser Scanning Confocal microscope. Images were obtained with a 40X dry objective and were processed using Imaris (Bitplane, Zurich, Switzerland). Quantitative analyses were performed using the COMSTAT software package [108]. Statistical significance was determined using Oneway ANOVA with Dunnett’s Multiple Comparison test. Two biological replicates were performed in triplicate. Images presented are from one representative experiment.

### Surface pilin ELISA

Surface pili composed of MshA were quantified using an ELISA based on a previously published protocol [92]. Briefly, overnight culture was diluted 1:100 in fresh LB medium and grown to OD_600_ 0.5 at 30°C. Cells (125μL) were added to a 96-well plate (Greiner Bio-One, Monroe, NC) and incubated at 30°C for one hour. Cells were fixed with 100μL of methanol for 10 minutes at room temperature, then washed twice with PBS. Samples were blocked in 5% nonfat dry milk and immunoblotted with polyclonal rabbit anti-MshA (1:1000 dilution, gift of J. Zhu) and horseradish peroxidase (HRP)-conjugated secondary antibody (Santa Cruz Biotechnology, Santa Cruz, CA). After three washes in PBS, 100μL of TMB (eBioscience, San Diego, CA) was added and incubated for 30 minutes at room temperature followed by the addition of 100μL of 2N H_2_SO_4_. Absorbance was recorded at 490nm and the samples were normalized to the change in WT. Two biological replicates were assayed in duplicate and statistical significance was determined with a Oneway ANOVA followed by a Dunnett’s Multiple Comparison test.

### Protein purification

*E*. *coli* BL21 harboring plasmids for gene expression were grown to an OD_600_ of 0.4 at 30°C in LB containing 100μg/mL ampicillin. Cultures were shifted to 18°C and IPTG was added to a final concentration of 100μM. 16h post induction, cells were harvested by centrifugation at 10,000 x g for 15 minutes and stored at -80°C. Cell pellets were resuspended in GST Lysis Buffer (25mM Tris pH 8.0, 0.5M NaCl containing PI cocktail tablets (Roche Life Science, Indianapolis, IN). Cells were lysed by sonication and cell lysate was cleared via centrifugation. Cleared lysate was loaded onto GS4B resin and washed with five column volumes of lysis buffer. Protein was eluted from the resin in 5mL elution buffer (25mM Tris pH8.0, 0.25M NaCl, 10mM glutathione). Samples were dialyzed against buffer (25mM Tris-HCl, 150mM NaCl, 250μM DTT, pH 7.5) overnight using 12 kDa cutoff dialysis tubing (Fisherbrand, Pittsburgh, PA) and concentrated to approximately 1mL using an Amicon 10KDa cutoff spin filter (EMD Millipore, Darmstadt, Germany). An aliquot of dialyzed protein was diluted in 6M guanidinium HCl and concentration determined via A_280_.

### ATPase assay

ATPase activity of purified proteins was determined by measuring the production of inorganic phosphate from ATP using the Enzchek Phosphate Assay Kit (Invitrogen). The standard reaction mixture was prepared with the addition of 2mM MgCl_2_, 10mM KCl, and 1mM DTT. Purified protein in buffer (25mM TrisHCl pH 7.5, 100mM NaCl) was added to the standard reaction mixture to a final concentration of 1μM with the indicated concentration of c-di-GMP. After a 10 minute incubation at room temperature, ATP was added to a final concentration of 10mM and reactions were incubated at 22°C for 30 minutes. Production of inorganic phosphate was monitored by reading OD_360_ and compared to a standard curve of solutions of KH_2_PO_4_. The experiment was performed in triplicate. Significance was determined via ANOVA and Bonferroni test.

## Supporting Information

S1 Fig Alignment of *V*. *cholerae* GGDEF domains. *V*. *cholerae* proteins which encode a GGDEF domain were aligned by Clustal-W. C-di-GMP binding was predicted by the presence of an RxxD motif (underlined in red) and DGC activity was predicted by the presence of a GGDEF motif (underlined in black). The presence of protein-encoding ORFs in the *V*. *cholerae* ORF library, potential c-di-GMP binding domains, prediction of activity, protein names, *V*. *cholerae* ORF numbers, and residues comprising the GGDEF domain are indicated. VCA0560 encodes 2 GGDEF domains.
(PDF)

S2 Fig Testing c-di-GMP binding to purified ORFs identified in the DRaCALA screen that lack known c-di-GMP binding domains. (A) Purified MBP-FliA (VC2066), MBP-RpoN (VC2529), MBP-TyrR (VC1308), and MBP-VCA0593 were separated on a 12% PAGE and stained with Coomassie Brilliant Blue. (B) ^32^P-c-di-GMP binding to purified MBP-FliA (VC2066), MBP-RpoN (VC2529), MBP-TyrR (VC1308), and MBP-VCA0593. All data are average of three independent assays and standard deviation is indicated by error bars.
(EPS)

S3 Fig Conserved amino acids in MshE revealed by BLAST-P and ClustalW alignment. (A) BLAST-P scores for proteins most homologous to MshN, MshE and MshG. The genus and species and the scores for each gene are indicated. The maximum score is for *V*. *cholerae*. (B) ClustalW alignment of the first 237 amino acids of MshE homologs is shown. Amino acids shaded in gray are 100% identical in all homologs. Asterisks indicate charged and polar residues targeted with site-directed mutagenesis. Position 151 indicates the C-terminus of Fragment 1 that retains c-di-GMP binding activity.
(EPS)

S4 Fig Purification of MshE point mutants and their ability to bind ^32^P-ATP. (A) Purified MshE and variants with indicated single alanine substitution were separated on a 12% PAGE and stained with Coomassie Brilliant Blue. (B) ^32^P-ATP binding to purified MshE and variants with indicated single alanine substitution. All data are average of three independent assays and standard deviation is indicated by error bars.
(PDF)

S5 Fig MshE binding to c-di-GMP enhances ATPase activity. ATPase activity of WT MshE and R9A proteins was assessed by detecting free phosphate released using the EnzCheck phosphate assay in the presence of 0, 10 and 33 μM c-di-GMP. Each condition was assayed with three independent reactions. Statistical analyses were performed using ANOVA followed by Bonferroni Multiple Comparison test. (** p<0.01).
(PDF)

S6 Fig Positions of c-di-GMP-binding residues in MshE sequence and structure. (A) Sequence alignment of the N-terminal fragment of *Vibrio cholerae* MshE (VC0705) with PA14_29490 from *Pseudomonas aeruginosa*, GspE proteins from *Xanthomonas campestris* (UniProt: GPSE_XANCP, PDB: 2D27) and *V*. *cholerae* (GPSE_VIBCH, PDB: 2BH1). (B) Structure of the N-terminal domain of GSPE_XANCP (PDB: 2D27), Arg9 and Gln32 are shown in stick representation.
(PDF)

S1 Table Fraction bound of ^32^P-c-di-GMP from primary DRaCALA screen of *V*. *cholerae* ORF library. (PDF)

S2 Table Strains used in this study. (PDF)

S3 Table Plasmids used in this study. (PDF)

S4 Table Primers used in this study. (PDF)
