# Supplementary material for: Systematic Identification of Cyclic-di-GMP Binding Proteins in Vibrio cholerae Reveals a Novel Class of Cyclic-di-GMP-Binding ATPases Associated with Type II Secretion Systems
Source: PLoS Pathog. 2015 Oct 27;11(10):e1005232. doi: 10.1371/journal.ppat.1005232 (PMC4624772; doi:10.1371/journal.ppat.1005232)
Supplement: S1 Fig — V. cholerae proteins which encode a GGDEF domain were aligned by Clustal-W. C-di-GMP binding was predicted by the presence of an RxxD motif (underlined in red) and DGC activity was predicted by the presence of a GGDEF motif (underlined in black). The presence of protein-encoding ORFs in the V. cholerae ORF library, potential c-di-GMP binding domains, prediction of activity, protein names, V. cholerae ORF numbers, and residues comprising the GGDEF domain are indicated. VCA0560 encodes 2 GGDEF domains. (PDF) [file ppat.1005232.s001.pdf]

Present in ORFeome	Domains	Predicted activity: Binding	Predicted activity: DGC	Protein name	VC ORF	Clustal-W alignment of GGDEF domains
Yes	GGDEF	Yes	Yes	CdgG	VC0900	364 AQLDPLTKVYNRSSFTER--LEHEYRRWIRTQHNLRVVLFDIDKFKSINDSFGYTAGDKALSIIARTIKKEL-RDSD-------TVA-RFSGEEFILLLPERSDNESYQIIHQIQLNVSKLPFKFRDKS------LTITLSAASIRFMDSDTPETVLDRLNLTLSEAKHIG--- 517
Yes	GGDEF	Yes	Yes		VC1104	335 AARDPMTGLLNRRGMESFLK-GKRHSQYL------AIAVLDIDDFKQINDAYGHDMGDRVICYIGEQIENHI-RSSD-------AVA-RFGGEEFVVYVTAKEKEQITRIMQRIFDAVCRESPLILEPG------FTISGGIEVVESTTDRSFEDLFKAADEKLYVAKTSGKNQ 486
Yes	GGDEF	Yes	Yes		VC1185	274 AHRDALTGAINRHGIRDWLQDQARLVRWQLNH--VSVLFIDIDYFKQVNDVYGHSLGDDLLREFALVISREI-RESD-------KLV-RWGGEEFVVFCAQTTLEQAIELAERLRAKIADHSWIHGG---------AITCSIGIAQMG-DERITETVSRADEALYRAKRLGRNR 426
Yes	GGDEF	Yes	Yes		VC1216	207 -MTDSLTGLPNRRKAMRQLVLHWNLAQDNQQP--FVCVMIDIDGFKAVNDHHGHDVGDKVLTTIANMLRDHF-RSDD-------LVC-RLGGDEFLVICPETNTAGGVYIAEQVCRAIQQQVISLENN---IRW-QGSVSMGVAAFASNMKDHHELLRAADQAVYLAKNSGKNR 364
Yes	GGDEF	Yes	Yes		VC1370	271 -ERDSLTGILNRRAFKQQLSHALIKAKRSNMK--LALLFFDLDKFKEINDTKGHEVGDLVLQEIAQRLKGSI-RESD--------FCGRLGGDEFVVCLDLLQDHSGVINKAYQMLEKL-QKPLQLN---QESL-EIGVSIGVALYPEQATQVPELLRIADEAMYTAKHQSGN- 426
Yes	GGDEF	Yes	Yes		VC1593	267 -YIDNLTRLNNRHFLN-KISTKFIEHPHSIA------AMIDIDHFKKINDQYGHITGDRILQAVASCLRKNV-RDGD-------TII-RFGGEEFLLLFQAHSNKEAWYMLDRLRQRVKDDHS--------LYG-TTISIG--FTFIDGS--LPAAISRADTALYQAKEAGRNQ 410
Yes	GGDEF	Yes	Yes		VC2370	411 -FLDSLTHVANRRRFDEQLHTLWHLHVREGKP--LSIILCDVDYFKDYNDAYGHLMGDETLKQIAIAFTQVANRHSD-------CVA-RYGGEEFGILLPNTPQSGAILVAERIHEKVRGLAIPHDHSKVADRI-TVSLGIVTLIPRPEDVP-EQMVELADRALYQAKANGRNQ 571
Yes	GGDEF	Yes	Yes		VCA0217	183 ----ALTGTLNRHQLDGFLKKCLRHRQLANES--AVIAVIDIDHFKSVNDLYGHDTGDKVITQVVEIMNTHC-RELD-------LLF-RLGGDEFLLLFENTSLTDATLVMSHIG-CRIQQTHYPYHAK------VTVSVGLAEALRTDDP--EQWFKRADQALYHSKKMGKNR 332
Yes	GGDEF	Yes	Yes	CdgD	VCA0697	481 --KDALTDLWNRRKFDQTISLECAKRRRYPDQAQSCLAIIDIDHFKRINDKFGHNEGDLVLRTVAKGIQDQL-RESD-------FIA-RIGGEEFAIIFPYTSIEEAEQVLNRVRLHIASLHHQQ------------VTLSGGVTDVCTS--PDQSYKRADLALYESKTSGRNQ 629
Yes	GGDEF	Yes	Yes		VCA0960	294 ALHDPLTHLPNRRYFIYTIEHYFENAKRSHSEGNFALLNIDIDRFKSINDSHGHSAGDKVLVACAERIKSSL-RVSD--------LVARIGGDEFLVLIPRIHREQDVLKVSDNILKRISETPIVYD---DKLI-HVRVSIGYALYDQSFATPDEMFKLADERMYTAKRRQ--- 451
Yes	GGDEF	Yes	Yes	CdgF	VCA0965	197 AYLDPLTGLANRWSFETWATEKLKEQQSSNTIT--ALVFLDIDNFKRINDSYGHDVGDQVLKHFAHRLRNNI-RNKDRATNQHDYSIARFAGDEFVLLLYGVRNLRDLDNILNRICNLFVDRYPETDM---LNN--LTVSIGAAIYPKDAITLPELTRCADKAMYAAKHGGKNQ 362
Yes	GGDEF	No	Yes	CdgB	VC1029	153 -HMDHLTQVYNRYAMDSLLPQALDIARLDRVS--SALLMIDIDCFKEYNDGYGHIQGDDVLRKVSQTLR-QWKS--------NLDLCFRYGGDEFLIFMTRVDAKQCQQRAEKLMAMIHELNIPHLSSRVADHV-TITVGIRHCDQIEKEMTAEKLVLVADKALFHAKHEQRG- 312
Yes	GGDEF	No	Yes	CdgH	VC1067	533 SHTDNLTKLRNR-------RALYNRYRRG-LSPRLSLVYLDVNTFKSINDQYGHEVGDKVLKQLAQRIE---AV--------WRGRSYRIGGDEFILIG----ECSAKRLEHVVAQCERFMFVDAER---DVSF-EVSVAIGIAKNRERTESLNEVMHQADIAMYRAKAEST-- 677
Yes	GGDEF	No	Yes		VC1353	809 --EDDLTGLANRRAFDMYLKQAFSRLQNPDQQ--VSIALLDIDHFKQINDRYSHIIGDQAIVAVSQELLGYVGD--------KTRVA-RWGGEEFTILYVGD-PKQAWGYFEKLRCKIEQVDLSAVAPGLN----VTVSIG--FADAQQAESYETVLKLADHALLTAKKLGRNR 962
Yes	GGDEF	No	No	CdgE	VC1367	497 --QDSLHGVLSRTSGLERLTHIEACQQTEHVH---LLALLDIDQLRQFNERHGYERGDIALQNTINTLLHQLRA---------KEFVCRLGDDEFLVVMPNCRRTLSETRLFVLHHALNGKTPTDAKS-----TVGLSVTLSYLAVESGLASFTHFYPKLDSALSIAKQSGN-- 649
Yes	GGDEF	No	Yes		VC1372	232 -IQDELTGAFNRRHFKEQVQ---QKLKQSKQKS-MVA-LVDIDHFKQINDSYGHDIGDSVITYVVRHLESLALT---------ECLVARYGGEEFAIFAREN---DFERVSQALDSVRDAISVGFHVEQQP-LK-VSVSIGAVIFES--SHEYGEVLRQADKALYQAKQQGRNR 383
Yes	GGDEF	No	Yes		VC1599	179 ASRDPLTGAHNRLSLTTSFQHFERFSDQTS----LCLLVIDLDFFKSINDQFGHDTGDKVLIETTRLFTQVVGD---------NNLY-RIGGEEFCVTLFDQSLEQAGRVCEHLRAIVSQHLFAFREKR------VQVTISIGVCEYQAGDQLNDLLKFADMELYRAKKAGRNQ 332
Yes	GGDEF	No	Yes		VC2224	357 AVRDKLTGAYNRQVFEELVDDAISLANKESHP--LSLAVIDLDHFKQVNDNYGHPAGDLVLQRVVALCQRHIAQ--------VGTLC-RWGGEEFVVLLPHMTQQEAYQRMESIRAEIAMHASQP-----------QVTVSIGVVQYQQNESLLHLFNRADQAMYTAKSEGRNK 508
Yes	GGDEF	No	Yes	VpvC	VC2285	340 -EHDPLTRISNRRHLEKQLK---SYLSDRPQAY-LVLFLVDIDFFKRFNDSFGHLAGDEALCSVADVLQSVEFH--------GEKIVARFGGEEFCVVLASDCAFDAEQYAQQMRSKIEQLAIANPVDALC-QY-LTVSIGGVYAISPKMESYLSLFHQADMALYHAKEHGRDR 498
Yes	GGDEF	No	Yes		VC2454	249 -EQDTLTGLKNRLGCEKFVLRKQR----ASTTM--VLLLIDLDGFKQVNDTLGHAAGDEVLREIAKRFYALAQT------HFSDFVVGRLGGDEFAIYIPLDEFVAESIERFAAQLIESSSEPIGLGQ---QSA-QVGCSIGISHMNSLHIDLEKLLLQADKAMYWVKYRGK-- 403
Yes	GGDEF	No	Yes		VC2697	156 -QIDPLTQLKNRRGFFT-----WSEPIAPETL--LGLIIFDIDHFKQINDTYGHPAGDYVLRQIAGLLEQNLP---------PLSCCVRWGGEEFLVCFECDDMANVIGLAENIRDYVESHTLMWQ----GQQI-ALTLSAGCTMGAMMSHNWDNLLHTADQALLSAKRLGRNR 307
Yes	GGDEF	No	Yes	CdgA	VCA0049	136 VFTDPLTKLHNRRWLDVKLKDLL----LHETP--FAFLVVDIDHFKSINDELSHLVGDKAIVNVSSELASYFKF--------RGASCVRFGGEEFLVILENVTSDMAQMHAETYRQRI-- 239
Yes	GGDEF	No	Yes		VCA0074	211 ALTDPLTGLENR-------TAMFAELERHRRSGGFSLFLLLDLNGFKQINDTYGHQMGDAVLKQVAYRLNNSIPS--------FDYRVFRMGGDEFAIILSSINSTEQMMMQRMIKQCFDHEFELSG----DLRA-KLNTSVGVSTYPLDSTNLSQLIHLADKNMYEMK------ 358
Yes	GGDEF	No	Yes		VCA0165	532 AKLDPLTRLGNRRMLEHQLEQTCEQTIKEVVN--YGVILLDIDHFGLFNNCYGHLEGDIALMRIGNILSRHAQS--------EHELFCRIGGEEFLLLVANRSAEEIHLLAENIRKSIEAECIEHCENPSGELL-TVSIGYAASRYKPREIQFDQLYAEADKALYRAKSQGRNQ 694
Yes	GGDEF	No	Yes		VCA0557	310 AKTDSLTDIANRRSFFEH------LEAEQTRSGSLTLMVFDIDDFKTINDRFGHGAGDNAICFVVGCVRQALAS--------DTYFA-RIGGEEFAIVARGKNAEESQQLAERICQRVAEKKWVVNAQHS-----LSLTISLGCAFYLHPARPFSLHD-ADSLMYEGKRNGKNQ 462
Yes	GGDEF	No	Yes		VCA0848	145 -ERDKLTGLLNRRTLEDRLRHTFAINPSTEENHKLWIAMLDIDHFKAINDHFGHMIGDEILLMFAQQMQHYFGP--------SSQLF-RFGGEEFVIIFSSGNEPQIKQQLDGFRQQIRRHNFP------RIGE-LSFSAG--FCSLRPGDYLPTILDHADKALYYAKEHGRNQ 299
Yes	GGDEF	No	Yes		VCA0939	157 ANTDSLTQLCNRRKLWADFRAAFARAKRLRQP--LSCISIDIDNFKLINDQFGHDKGDEVLCFLAKLFQSVIS---------DHHFCGRVGGEEFIIVLENTHVETAFHLAEQIRQRFAEHPFFEQ----NEHI-YL--CAGVSSLHHGDHDIADIYRRSDQALYKAKRNGRNR 312
Yes	GGDEF	No	Yes		VCA0956	180 ALFDSLSGLYNRRAFDGDM----FTLIHAGQQ--VSLIMLDIDHFKALNDNYGHLFGDQIIRAIAKRLQSLCRD--------GVTAY-RYGGEEFALIAPHKSLRIARQFAESVRRSIEKLTVKDRRSGQSVGS-ITASFG--VVEKIEGDSLESLIGRADGLLYEAKNLGRNR 335
Yes	GGDEF	No	Yes		VCA0560_A	238 AFNDPLTQIPGRQALDQD-------LKHIGRK--FTLAMLDVDHFKKFNDTYGHDTGDDVLRLVASRLREIN-G--------KARVY-RYGGEEFSIIYKGKLAKEVLPFIEALRQDIE--- 337
Yes	GGDEF, EAL	No	Yes		VC0072	338 ARYDLLTHTLNRNQFELELAKALKETDSQLRTH--AMLYLDLDQLKVLNDTAGHDAGDGAIQFCASMLEDVLPF---------KATLARMGGDEFSVLLR-DCTERDAVLVAQSIIHALSEVAFVWEH---IRF-NLTCSIGIRMIDHTATSPQMVHAQADTACHAAKEEGRNR 495
Yes	GGDEF, EAL	No	Yes		VC0653	256 AHIDSLTGLYNRHGFTKRLEQCIQ----NETPL--VMLYLDIDNFKNINDSLGHHIGDKVIKEVAARLKRLLP---------QQAVLGHLGGDEFGLILPEPEHN-RSAEMLADRIISLINQPFDLHH---FSK-RLACSIGSVRYPGDGNDARVLLQNADTAMYEAKERGRNR 409
Yes	GGDEF, EAL	No	Yes		VC0658	242 AYYDSLTGLANRSLLLQKLSDAIETAQNSHSTG--VLFYLDLDRFKTINDSLGHTIGDQLIKAVALRMEAWTKN---------DYLSARIGGDEFAVLIP-YLSPAKAEEVAKQLLTLISN-PYAVDD---HQF-YCTVSIGISVFPSVGSCNIDVLRQADTALYRAKASGRNK 398
Yes	GGDEF, EAL	No	Yes	MbaA	VC0703	340 -ENDHLTKLANRYQFQVQADLLLSRCYDTQHI---WVMYIDLDNFKYVNDKYGHQIGDSLLVSFATHVRQLCKNFEASHNTYS--IAARLSGDEFAILLVSPKRFNDCAKIFAQRLLAPIQNKDNSPL---SHF-PITASIGIATFPKDGEHIEKLLLNADTAMYQAKNAGKNQ 503
Yes	GGDEF, EAL	No	No		VC1934	212 AWHDPLTRLKNRNFIVKKLKKRRRNQQEP-----IALILFDLNRFKELNDTMGYAFGDQLLINIAELLTQRCRS--------FAYQCARIGADEFAVLLHPCTGNADFFIRNLWNDLTKLVQENDPTKRLSVAMGVVTCQTQDFTESSSKLRASSLLNNADLALNIAKKAPE-- 370
Yes	GGDEF, EAL	No	Yes		VC2750	430 --YDSLTQLLSREGLVEHCHQIPQLNG--------GLILIGIDKFRDINDSLGHHQADQLLVAIAQRLKVTFPEP---------ALIARIGGDEFAIYLPDVNQQDDLKSVATDILKLFASPFSMPSD-------NIAVNVSIGIVYLEEPNMTVWLRNGSIALSNAKAEH--- 574
Yes	GGDEF, EAL	No	No		VCA0080	181 -SHDNLTGLMNRNDLAEKLTALVQEDRHH-----FTLAFLDIDEFRSINDLHGHYLGDLVLKFVADAIKQAVPE---------EGYAFRIAADEFAFLTTDRE--PMKICQTILNKLAQDYIDQDRRLKISVSIGITKYS-------GEKLNADQLLFNASLALKECKRNHN-- 328
Yes	GGDEF, EAL	No	No	CdgC	VCA0785	205 --HDLGTGFLNRTALEQQLAMQLAQLAEHEEL---AVIHIGFANARQLQARLGYHLWDDVLKQLRERLGPVTEG---------ELLTARPNSTNLTLILKAHPLDTQLNQLCHRLIHAGQAQFVTEGLP------VHLNPYIGVALSRETRDPQQLLRHAVSSMLACKDSG--- 355
No	GGDEF	No	No		VCA1082	237 AYLDPVSHLGNRAYYMSQLSGWLSESGIG------GVAILQAEFIKELYEEKGYEAGDGMVRELADRLKNSITIK--------DISIARISTYEFGIIMPNMDETELKIVAESIITCVDDINPDPTG-------MAKANLSLGVVSNKRQSSTTTLLSLLDNALLKRN------ 383
No	GGDEF	No	Yes		VC1376	361 -EHDSLTNIYNRRFFSQH------LCKMLDEKQSFTLISFDIDRFKQINDSYGHLAGDYALTHVVDVVKKELVE--------SDIFA-RFGGDEFAILSSVTDETALYTYLERIRKVVEAEPVMLNAETP-----LTLTISIGASINCEYSEPEILQS-VDDQLYLSKSKGRNR 512
No	GGDEF, EAL	Yes	No	CsrD	VC0398	232 -FLDQLTGTANRVLYDSKLESALLESGAHG-----GVMMLRVDDLESAREESPKRTLDEFIIEVGECLSNIVQRYPD-------AILSRYYEDVFALFIPHQGSKDIAQVATQAIKLIERINPPEPLP-------EDNWFHIGVTMYQEGERRGRIIDEMETALKSAQLQGVN- 384
