# Supplementary figures and images for: Systematic Identification of Cyclic-di-GMP Binding Proteins in Vibrio cholerae Reveals a Novel Class of Cyclic-di-GMP-Binding ATPases Associated with Type II Secretion Systems
Source: PLoS Pathog. 2015 Oct 27;11(10):e1005232. doi: 10.1371/journal.ppat.1005232 (PMC4624772; doi:10.1371/journal.ppat.1005232)

**A**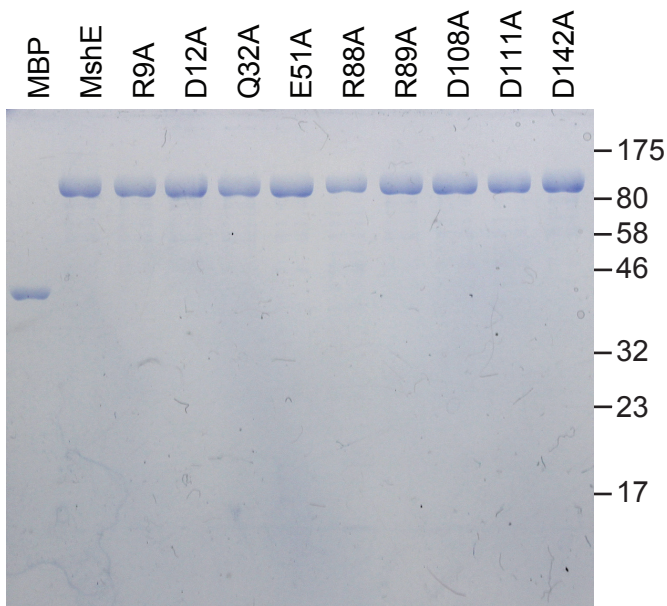**B**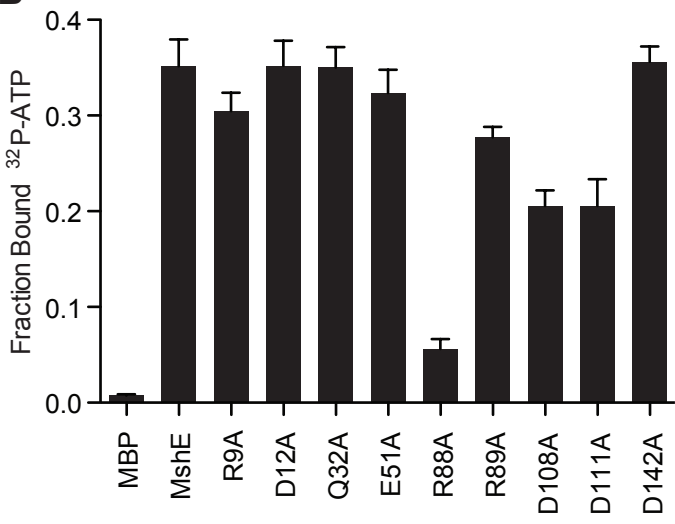

Supplement: S4 Fig — (A) Purified MshE and variants with indicated single alanine substitution were separated on a 12% PAGE and stained with Coomassie Brilliant Blue. (B) 32P-ATP binding to purified MshE and variants with indicated single alanine substitution. All data are average of three independent assays and standard deviation is indicated by error bars. (PDF) [file ppat.1005232.s004.pdf]

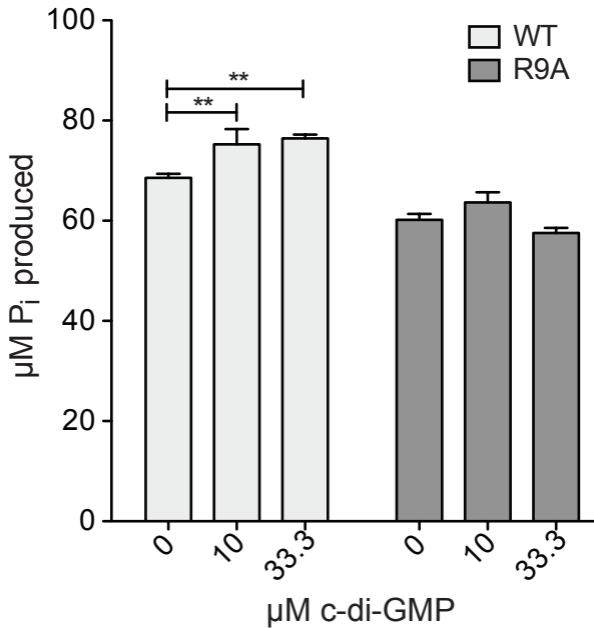

Supplement: S5 Fig — ATPase activity of WT MshE and R9A proteins was assessed by detecting free phosphate released using the EnzCheck phosphate assay in the presence of 0, 10 and 33 μM c-di-GMP. Each condition was assayed with three independent reactions. Statistical analyses were performed using ANOVA followed by Bonferroni Multiple Comparison test. (** p<0.01). (PDF) [file ppat.1005232.s005.pdf]
