# Supplementary material for: Systematic Identification of Cyclic-di-GMP Binding Proteins in Vibrio cholerae Reveals a Novel Class of Cyclic-di-GMP-Binding ATPases Associated with Type II Secretion Systems
Source: PLoS Pathog. 2015 Oct 27;11(10):e1005232. doi: 10.1371/journal.ppat.1005232 (PMC4624772; doi:10.1371/journal.ppat.1005232)
Supplement: S6 Fig — (A) Sequence alignment of the N-terminal fragment of Vibrio cholerae MshE (VC0705) with PA14_29490 from Pseudomonas aeruginosa, GspE proteins from Xanthomonas campestris (UniProt: GPSE_XANCP, PDB: 2D27) and V. cholerae (GPSE_VIBCH, PDB: 2BH1). (B) Structure of the N-terminal domain of GSPE_XANCP (PDB: 2D27), Arg9 and Gln32 are shown in stick representation. (PDF) [file ppat.1005232.s006.pdf]

A

```
MshE VIBCH      1   MPINKLRKRLGDLLVEEGIVSEAQLEQALNAQKNTGRRLGDTLISLGFLSETQLLNFLAQ   60
PA14_29490     14   AEWIPLGQRLLERGLVS----GQELERALDLQRRLGGRLGGILVRSGAISENTLMQVLAE   69
GSPE_XANCP      1   MEQRSAETRIVEALLERRRLKDTDLLRARQLQAESGMGLLALLGRLGLVSERDHAETCAE   60
2D27/SEC_STR        -----HHHHHHHHHHH-----HHHHHHHHHHHHHH---HHHHHHH-----HHHHHHHHHH

MshE VIBCH     61   QLSLPVIDLSRAHVDIDAV--PLLP---EVHARRLRALVIGRSG-DTLRIAMSDPADLFA  114
PA14_29490     70   QLRLPLVGDDLRKPSEESIAAFLLSTPINTDWFVEEQLVVWEEG-EQLLFAARDPLSPSI  128
GSPE_XANCP     61   VLGLPLVDARQLGDTPPEM-LPEVQGLSLRFLKQFHLCPVGERD-GRLDLWIADPYDDYA  118
GSPE_VIBCH      1   -------MTEMVISPAERQ--SIRR-LPFSFANRFKLVLDWNEDFSQASIYYLAPLSMEA   50
2D27/SEC_STR        HH--------------------------HHHHHHH-EEEEEE-----EEEEE-----HHH
2BH1/SEC_STR        ----------------------------HHHHHHH-EEEEEE-----EEEEEE----HHH

MshE VIBCH    115   QEALLNQLPDYGFEFVIAPEKQLVDGFDRYYRRTKEI 151
PA14_29490    129   RETLGYFYPERSIQAVLCRSQDLDGWLEHSLDLARQG 165
GSPE_XANCP    117   IDAVRLATG-LPLLLHVGLRSEIDDLIERWYGQGRSA 154
GSPE_VIBCH     51   LVETKRVVK-HAFQLIELSQAEFESKLTQVYQRDSSE  86
2D27/SEC_STR        HHHHHHHH----EEEEE--HHHHHHHHHHH-------
2BH1/SEC_STR        HHHHHHHH----EEEEEE-HHHHHHHHHHHH------
```

B

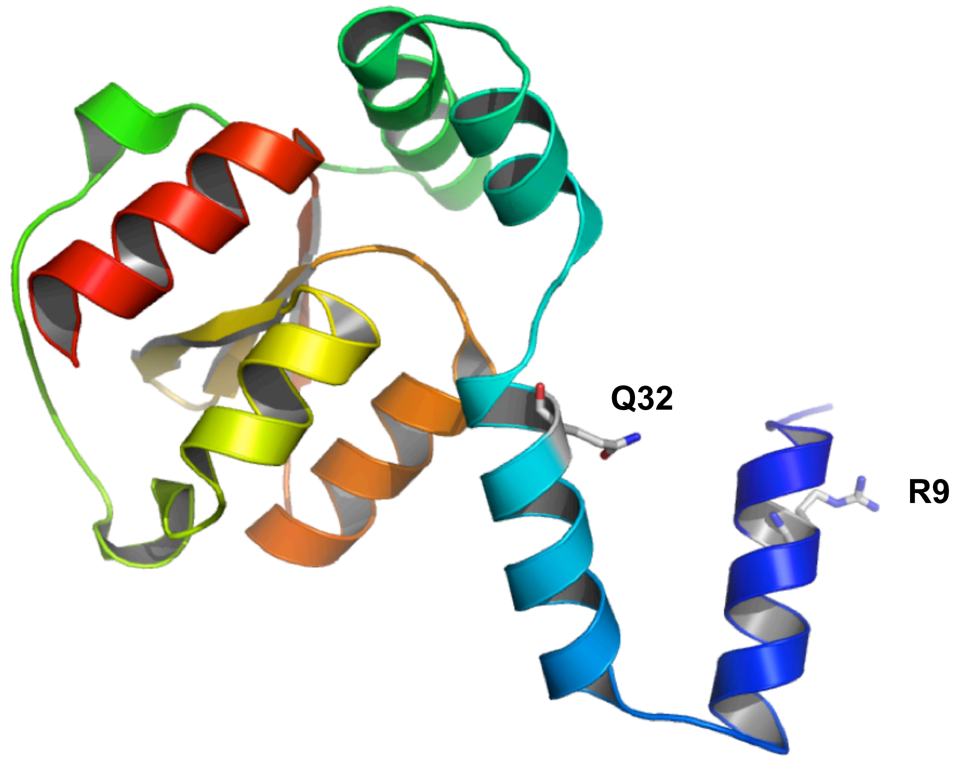

Figure S6. Positions of c-di-GMP-binding residues in MshE sequence and structure.
