# Supplementary material for: Systematic Identification of Cyclic-di-GMP Binding Proteins in Vibrio cholerae Reveals a Novel Class of Cyclic-di-GMP-Binding ATPases Associated with Type II Secretion Systems
Source: PLoS Pathog. 2015 Oct 27;11(10):e1005232. doi: 10.1371/journal.ppat.1005232 (PMC4624772; doi:10.1371/journal.ppat.1005232)
Supplement: S1 Table — (PDF) [file ppat.1005232.s007.pdf]

S1 Table: Fraction Bound ^{32}P-c-di-GMP in Primary DRaCALA Screen of *V. cholerae* ORFs

VC ORF #	His-ORF Fraction Bound		His-MBP-ORF Fraction Bound	
	Replicate 1	Replicate 2	Replicate 1	Replicate 2
VC0001	0.09	0.07	0.02	0.00
VC0002	0.04	0.05	0.06	0.06
VC0003	0.04	0.03	0.07	0.08
VC0004	0.04	0.06	0.06	0.08
VC0005	0.09	0.05	0.05	0.07
VC0006	0.03	0.04	0.06	0.05
VC0007	0.00	0.04	0.05	0.06
VC0008	0.03	0.04	0.06	0.04
VC0009	0.05	0.06	0.06	0.07
VC0010	0.05	0.05	0.05	0.06
VC0011	0.05	0.05	0.03	0.03
VC0012	0.01	0.02	0.01	0.02
VC0013	0.01	0.01	0.02	0.00
VC0014	0.03	0.04	0.04	0.03
VC0015	0.05	0.08	0.05	0.05
VC0016	0.04	0.06	0.05	0.04
VC0017	0.09	0.09	0.03	0.05
VC0018	0.02	0.02	0.06	0.09
VC0019	0.06	0.06	0.05	0.06
VC0020	0.09	0.10	0.02	0.02
VC0021	0.04	0.03	0.09	0.13
VC0022	0.04	0.04	0.04	0.02
VC0023	0.10	0.10	0.01	0.03
VC0024	0.04	0.05	0.08	0.06
VC0025	0.10	0.10	0.03	0.05
VC0026	0.04	0.05	0.08	0.06
VC0027	0.03	0.05	0.07	0.04
VC0028	0.03	0.03	0.10	0.06
VC0029	0.03	0.02	0.06	0.08
VC0030	0.06	0.06	0.06	0.08
VC0031	0.05	0.06	0.04	0.04
VC0032	0.08	0.08	0.03	0.03
VC0033	0.08	0.08	0.04	0.09
VC0034	0.05	0.04	0.04	0.05
VC0035	0.05	0.05	0.05	0.04
VC0036	0.03	0.04	0.04	0.05
VC0037	0.07	0.10	0.06	0.07
VC0038	0.09	0.06	0.06	0.05

VC0039	0.04	0.04	0.04	0.05
VC0040	0.04	0.07	0.06	0.06
VC0041	0.03	0.05	0.06	0.06
VC0042	-0.01	0.00	0.06	0.06
VC0043	0.01	0.03	0.05	0.04
VC0044	0.04	0.06	0.04	0.05
VC0045	0.04	0.06	0.04	0.06
VC0046	0.03	0.03	0.00	0.00
VC0047	0.02	0.01	0.01	0.02
VC0048	0.03	0.03	0.04	0.04
VC0049	0.04	0.05	0.05	0.05
VC0050	0.04	0.06	0.06	0.07
VC0051	0.05	0.04	0.07	0.05
VC0052	0.06	0.06	0.07	0.07
VC0053	0.05	0.06	0.07	0.05
VC0054	0.03	0.05	0.07	0.07
VC0055	0.04	0.06	0.06	0.05
VC0056	0.02	0.05	0.04	0.04
VC0057	0.05	0.05	0.05	0.06
VC0058	0.06	0.07	0.09	0.07
VC0060	0.05	0.06	0.05	0.05
VC0061	0.05	0.05	0.04	0.06
VC0062	0.06	0.09	0.06	0.05
VC0063	0.10	0.13	0.08	0.07
VC0064	0.06	0.08	0.04	0.06
VC0065	0.05	0.05	0.05	0.06
VC0066	0.01	0.03	0.05	0.06
VC0067	0.05	0.05	0.06	0.06
VC0068	0.04	0.05	0.03	0.03
VC0069	0.04	0.05	0.05	0.05
VC0070	0.09	0.10	0.03	0.04
VC0071	0.02	0.03	0.10	0.12
VC0072	0.55	0.53	0.48	0.53
VC0072	0.01	0.04	0.05	0.06
VC0073	0.05	0.04	0.13	0.17
VC0074	0.06	0.06	0.02	0.04
VC0075	0.05	0.06	0.08	0.09
VC0076	0.06	0.06	0.05	0.05
VC0077	0.07	0.10	0.02	0.03
VC0078	0.00	0.00	0.05	0.09
VC0079	0.08	0.08	0.10	0.03
VC0080	0.03	0.03	0.03	0.04
VC0081	0.05	0.06	0.07	0.05

VC0082	0.00	0.02	0.01	0.02
VC0083	0.07	0.07	0.04	0.03
VC0084	0.04	0.03	0.06	-0.01
VC0085	0.04	0.04	0.04	0.04
VC0086	0.05	0.06	0.01	0.02
VC0087	0.04	0.02	0.05	0.06
VC0088	-0.02	0.01	0.05	0.04
VC0089	0.03	0.02	0.04	0.05
VC0090	0.04	0.03	0.05	0.05
VC0091	0.02	0.02	0.06	0.08
VC0092	0.07	0.08	0.03	0.03
VC0093	0.05	0.06	0.06	0.06
VC0094	0.06	0.06	0.05	0.04
VC0095	0.04	0.04	0.05	0.04
VC0096	0.09	0.06	0.06	0.07
VC0097	0.04	0.03	0.13	0.10
VC0098	0.13	0.14	0.05	0.05
VC0099	0.05	0.05	0.02	0.02
VC0100	0.10	0.11	0.06	0.06
VC0101	0.09	0.07	0.04	0.04
VC0102	0.04	0.07	0.06	0.07
VC0103	0.07	0.08	0.06	0.04
VC0104	0.05	0.05	0.04	0.03
VC0105	0.05	0.04	0.05	0.02
VC0106	0.06	0.07	0.09	0.08
VC0107	0.04	0.05	0.05	0.05
VC0108	0.02	0.03	0.03	0.02
VC0109	0.02	0.06	0.04	0.05
VC0110	0.05	0.08	0.04	0.04
VC0111	0.09	0.10	0.06	0.06
VC0112	0.09	0.11	0.07	0.07
VC0113	0.12	0.10	0.09	0.09
VC0114	0.09	0.08	0.07	0.06
VC0115	0.12	0.12	0.07	0.08
VC0116	0.04	0.05	0.02	0.03
VC0117	0.13	0.14	0.06	0.06
VC0118	0.05	0.05	0.03	0.04
VC0119	0.09	0.09	0.02	0.03
VC0120	0.06	0.08	0.00	0.04
VC0121	0.08	0.07	0.04	0.04
VC0123	0.07	0.07	0.04	0.03
VC0124	0.09	0.11	0.05	0.04
VC0125	0.07	0.08	0.05	0.06

VC0126	0.09	0.08	0.03	0.03
VC0127	0.09	0.10	0.04	0.04
VC0128	0.09	0.09	0.04	0.04
VC0129	0.07	0.07	0.06	0.04
VC0130	0.00	0.01	0.01	0.02
VC0131	0.11	0.12	0.07	0.06
VC0132	0.09	0.08	0.06	0.06
VC0133	0.05	0.05	0.04	0.04
VC0133	0.06	0.06	0.04	0.05
VC0134	0.06	0.05	0.04	0.04
VC0135	0.05	0.06	0.07	0.06
VC0136	0.06	0.06	0.03	0.04
VC0137	0.01	0.03	0.00	-0.01
VC0138	0.05	0.06	0.06	0.07
VC0139	0.11	0.11	0.07	0.07
VC0140	0.11	0.12	0.06	0.06
VC0141	0.03	0.06	0.04	0.08
VC0142	0.07	0.05	0.03	0.03
VC0143	0.01	0.02	0.00	0.01
VC0144	0.08	0.08	0.07	0.07
VC0145	0.02	0.04	0.05	0.05
VC0146	0.03	0.03	0.02	0.03
VC0147	0.05	0.06	0.03	0.04
VC0148	0.01	0.02	0.00	0.00
VC0149	0.02	0.03	0.01	0.01
VC0150	0.08	0.06	0.05	0.07
VC0151	0.02	0.02	0.01	0.01
VC0152	0.07	0.06	0.04	0.04
VC0153	0.02	0.02	0.01	0.01
VC0154	0.02	0.00	0.00	0.00
VC0156	0.04	0.05	-0.02	-0.02
VC0157	0.03	0.04	0.05	0.03
VC0158	0.06	0.10	0.06	0.06
VC0159	0.02	0.03	0.01	0.02
VC0160	0.07	0.07	0.04	0.05
VC0161	0.03	0.03	0.04	0.03
VC0162	0.09	0.09	0.05	0.08
VC0163	0.05	0.13	0.06	0.06
VC0164	0.05	0.06	0.04	0.03
VC0165	0.02	0.03	0.00	0.00
VC0166	0.02	0.02	0.01	0.02
VC0167	0.03	0.05	0.03	0.03
VC0168	0.05	0.04	0.07	0.07

VC0169	0.07	0.07	0.02	0.03
VC0170	0.08	0.10	0.04	0.08
VC0171	0.03	0.05	0.03	0.03
VC0172	0.09	0.08	0.02	0.03
VC0173	0.04	0.04	0.00	0.02
VC0174	0.02	0.02	0.01	0.02
VC0175	0.04	0.05	0.04	0.04
VC0176	0.11	0.08	0.03	0.05
VC0177	0.03	0.04	0.02	0.02
VC0178	0.03	0.03	0.01	0.02
VC0179	0.04	0.03	0.04	0.04
VC0180	0.07	0.08	0.07	0.05
VC0181	0.01	0.02	0.01	0.02
VC0182	0.01	0.02	0.02	0.03
VC0183	0.03	0.03	0.05	0.05
VC0184	0.08	0.09	0.05	0.05
VC0185	0.02	0.02	0.01	0.01
VC0186	0.07	0.07	0.05	0.06
VC0187	0.01	0.02	0.01	0.02
VC0188	0.06	0.07	0.03	0.03
VC0189	0.05	0.05	0.03	0.03
VC0189	0.04	0.05	0.03	0.04
VC0190	0.01	0.02	0.08	0.08
VC0191	0.06	0.06	0.04	0.04
VC0192	0.01	0.02	0.01	0.01
VC0193	0.08	0.09	0.05	0.08
VC0194	0.09	0.08	0.08	0.12
VC0195	0.04	0.05	0.01	0.01
VC0196	0.08	0.06	0.08	0.07
VC0197	0.05	0.05	0.01	0.01
VC0198	0.01	0.02	-0.01	0.00
VC0199	0.09	0.09	0.14	0.10
VC0200	0.01	0.01	0.02	0.02
VC0201	0.02	0.06	0.06	0.05
VC0202	0.08	0.10	0.04	0.03
VC0203	0.01	0.03	0.04	0.04
VC0204	0.00	0.00	0.10	0.06
VC0205	0.08	0.07	0.06	0.03
VC0206	0.01	0.02	0.16	0.13
VC0207	0.02	0.04	0.03	0.03
VC0208	0.01	0.03	0.03	0.02
VC0209	0.05	0.05	0.06	0.04
VC0210	0.00	0.02	0.11	0.10

VC0211	0.06	0.06	0.04	0.04
VC0212	0.01	0.00	0.12	0.09
VC0213	0.00	0.00	0.12	0.08
VC0214	0.06	0.05	0.07	0.02
VC0215	0.03	0.03	0.03	0.03
VC0216	0.04	0.06	0.04	0.02
VC0217	0.03	0.06	0.04	0.04
VC0218	0.06	0.04	0.04	0.03
VC0219	0.04	0.05	0.06	0.04
VC0220	0.05	0.05	0.02	0.02
VC0221	0.00	0.00	0.07	0.05
VC0222	0.06	0.06	0.06	0.04
VC0223	0.02	0.03	0.08	0.07
VC0224	0.01	0.02	0.09	0.06
VC0225	0.04	0.07	0.04	0.04
VC0226	0.07	0.10	0.04	0.04
VC0227	0.01	0.00	0.04	0.04
VC0228	0.06	0.06	0.07	0.05
VC0229	-0.01	0.00	0.08	0.06
VC0230	0.02	0.01	0.02	0.03
VC0231	0.06	0.06	0.06	0.06
VC0232	0.04	0.05	0.06	0.02
VC0233	0.00	0.03	0.07	0.06
VC0234	0.00	0.01	0.05	0.04
VC0235	0.05	0.10	0.04	0.04
VC0236	0.00	0.00	0.08	0.05
VC0237	-0.01	0.01	0.12	0.11
VC0238	0.01	0.01	0.09	0.06
VC0239	0.01	0.04	0.06	0.05
VC0240	0.10	0.08	0.02	0.02
VC0241	-0.01	0.00	0.12	0.11
VC0242	0.00	0.01	0.15	0.13
VC0243	0.02	0.04	0.03	0.03
VC0244	0.00	0.02	0.09	0.05
VC0245	0.01	0.03	0.04	0.04
VC0246	0.04	0.05	0.03	0.03
VC0247	0.02	0.02	0.07	0.06
VC0248	0.06	0.06	0.06	0.02
VC0249	0.01	0.02	0.05	0.04
VC0250	0.00	0.03	0.02	0.01
VC0251	0.03	0.04	0.05	0.05
VC0252	0.01	0.01	0.17	0.17
VC0253	0.06	0.06	0.03	0.04

VC0254	0.02	0.02	0.08	0.08
VC0254	0.01	0.02	0.05	0.05
VC0255	0.04	0.04	0.03	0.03
VC0256	0.01	0.01	0.09	0.07
VC0257	0.02	0.02	0.05	0.05
VC0258	0.01	0.00	0.02	0.03
VC0259	0.00	0.01	0.05	0.04
VC0260	0.04	0.06	0.07	0.06
VC0261	0.04	0.03	0.06	0.05
VC0262	0.00	0.02	0.03	0.02
VC0263	0.05	0.07	0.02	0.05
VC0264	0.09	0.08	0.05	0.07
VC0265	0.04	0.03	0.05	0.04
VC0266	0.03	0.06	0.04	0.07
VC0267	0.03	0.05	0.11	0.09
VC0268	0.07	0.04	0.02	0.07
VC0269	0.03	0.03	0.08	0.06
VC0270	0.08	0.04	0.08	0.09
VC0271	0.01	0.04	0.08	0.07
VC0272	0.09	0.07	0.07	0.03
VC0273	0.04	0.04	0.06	0.06
VC0274	0.05	0.04	0.07	0.09
VC0275	0.03	0.03	0.06	0.06
VC0276	0.08	0.09	0.06	0.06
VC0277	0.11	0.11	0.07	0.06
VC0278	0.07	0.04	0.04	0.03
VC0279	0.05	0.04	0.04	0.05
VC0280	0.03	0.03	0.05	0.04
VC0281	0.05	0.02	0.03	0.03
VC0282	0.06	0.06	0.08	0.06
VC0283	0.06	0.07	0.06	0.06
VC0284	0.05	0.04	0.05	0.06
VC0285	0.06	0.06	0.06	0.09
VC0286	0.03	0.03	0.01	0.03
VC0286	0.00	0.00	0.04	0.05
VC0287	0.02	0.02	0.04	0.04
VC0287	0.01	0.05	0.05	0.05
VC0288	0.06	0.07	0.04	0.07
VC0289	0.07	0.07	0.10	0.09
VC0290	0.07	0.07	0.08	0.13
VC0291	0.07	0.05	0.09	0.07
VC0292	0.07	0.06	0.02	0.03
VC0293	0.06	0.06	0.03	0.04

VC0294	0.08	0.10	0.10	0.11
VC0295	0.11	0.09	0.08	0.07
VC0297	0.10	0.08	0.07	0.04
VC0298	-0.01	0.03	0.07	0.06
VC0299	0.05	0.08	0.03	0.04
VC0300	0.07	0.07	0.04	0.04
VC0301	0.06	0.06	0.07	0.05
VC0302	0.06	0.06	0.03	0.05
VC0303	0.05	0.08	0.06	0.08
VC0304	0.03	0.02	0.11	0.07
VC0305	0.03	0.06	0.12	0.12
VC0306	0.05	0.05	0.04	0.06
VC0307	0.08	0.10	0.07	0.07
VC0308	0.02	0.06	0.04	0.03
VC0309	0.01	0.02	0.04	0.04
VC0311	0.03	0.04	0.04	0.05
VC0312	0.05	0.08	0.03	0.03
VC0313	0.05	0.05	0.06	0.06
VC0314	0.06	0.06	0.07	0.06
VC0316	0.05	0.06	0.02	0.03
VC0317	0.06	0.08	0.05	0.11
VC0318	0.03	0.04	0.07	0.07
VC0319	0.07	0.08	0.07	0.05
VC0320	0.04	0.03	0.04	0.04
VC0321	0.02	0.02	0.00	0.00
VC0322	0.05	0.07	0.05	0.04
VC0323	0.11	0.10	0.06	0.06
VC0324	0.03	0.03	0.01	0.02
VC0326	0.01	0.03	0.01	0.01
VC0327	0.10	0.06	0.05	0.07
VC0328	0.03	0.03	0.05	0.06
VC0329	0.06	0.07	0.06	0.04
VC0330	0.02	0.03	0.06	0.07
VC0331	0.04	0.04	0.09	0.08
VC0332	0.03	0.03	0.00	0.01
VC0333	0.04	0.04	0.08	0.07
VC0334	0.04	0.04	0.03	0.03
VC0335	0.08	0.08	0.05	0.05
VC0336	0.06	0.07	0.06	0.05
VC0337	0.05	0.04	0.03	0.01
VC0338	0.06	0.06	0.06	0.06
VC0339	0.02	0.03	0.04	0.04
VC0341	0.08	0.08	0.07	0.07

VC0342	0.06	0.06	0.03	0.04
VC0343	0.06	0.07	0.03	0.02
VC0344	0.06	0.05	0.01	0.02
VC0345	0.08	0.10	0.06	0.05
VC0346	0.04	0.06	0.05	0.05
VC0347	0.12	0.10	0.10	0.09
VC0348	0.02	0.02	0.06	0.07
VC0349	0.04	0.03	0.09	0.08
VC0350	0.05	0.05	0.03	0.03
VC0351	0.02	0.03	0.05	0.05
VC0352	0.06	0.04	0.03	0.03
VC0353	0.03	0.02	0.05	0.06
VC0354	0.01	0.01	0.02	0.02
VC0355	0.06	0.07	0.06	0.06
VC0356	0.07	0.07	0.06	0.05
VC0357	0.05	0.05	0.09	0.07
VC0358	0.04	0.05	0.04	0.07
VC0359	0.07	0.08	0.06	0.07
VC0360	0.06	0.06	0.10	0.10
VC0361	0.06	0.07	0.04	0.05
VC0362	0.01	0.02	0.00	0.00
VC0363	0.07	0.07	0.03	0.04
VC0364	0.07	0.06	0.03	0.03
VC0365	0.06	0.05	0.06	0.05
VC0366	0.07	0.10	0.03	0.04
VC0367	0.03	0.04	0.08	0.05
VC0368	0.06	0.07	0.03	0.03
VC0369	0.04	0.04	0.05	0.04
VC0370	0.03	0.04	0.04	0.04
VC0371	0.03	0.04	0.01	0.01
VC0372	0.03	0.02	0.04	0.04
VC0373	0.06	0.07	0.04	0.03
VC0374	0.05	0.06	0.11	0.10
VC0375	0.05	0.05	0.07	0.06
VC0376	0.07	0.07	0.03	0.04
VC0377	0.02	0.02	0.04	0.04
VC0378	0.06	0.07	0.06	0.04
VC0379	0.04	0.03	0.03	0.03
VC0380	0.09	0.09	0.05	0.03
VC0381	0.02	0.02	0.04	0.04
VC0382	0.05	0.06	0.06	0.06
VC0383	0.08	0.06	0.10	0.04
VC0384	0.04	0.04	0.07	0.03

VC0385	0.01	0.03	0.05	0.05
VC0386	0.07	0.08	0.07	0.05
VC0387	0.07	0.08	0.08	0.07
VC0388	0.07	0.06	0.06	0.08
VC0389	0.02	0.02	0.03	0.05
VC0390	0.04	0.06	0.02	0.02
VC0391	0.03	0.03	0.04	0.07
VC0392	0.03	0.05	0.06	0.07
VC0393	0.05	0.05	0.08	0.07
VC0394	0.08	0.06	0.12	0.09
VC0395	0.03	0.04	0.05	0.08
VC0396	0.06	0.09	0.08	0.08
VC0397	0.03	0.04	0.06	0.06
VC0399	0.00	0.01	0.04	0.03
VC0400	0.05	0.06	0.07	0.06
VC0401	0.01	0.02	0.07	0.06
VC0402	0.01	0.00	0.01	0.02
VC0403	0.03	0.01	0.03	0.02
VC0404	0.02	0.02	0.10	0.08
VC0405	0.17	0.19	0.23	0.24
VC0406	0.00	0.02	0.05	0.04
VC0407	0.03	0.02	0.05	0.05
VC0408	0.00	0.00	0.05	0.05
VC0409	0.05	0.05	0.01	0.03
VC0410	0.01	0.01	0.04	0.04
VC0411	0.01	0.01	0.01	0.03
VC0412	0.00	0.00	0.03	0.03
VC0413	0.05	0.04	0.04	0.04
VC0414	0.24	0.23	0.04	0.04
VC0415	0.01	0.02	0.01	0.01
VC0416	0.00	0.02	0.07	0.08
VC0417	0.06	0.05	0.03	0.02
VC0418	0.02	0.01	0.03	0.04
VC0419	-0.01	0.00	0.06	0.07
VC0420	0.04	0.03	0.01	0.04
VC0420	0.01	0.02	0.04	0.05
VC0421	0.02	0.04	0.05	0.07
VC0422	0.00	0.00	0.05	0.04
VC0423	0.02	0.03	0.06	0.03
VC0424	0.04	0.04	0.08	0.06
VC0425	0.01	0.01	0.04	0.05
VC0426	0.01	0.03	0.04	0.04
VC0427	0.05	0.06	0.03	0.03

VC0428	0.01	0.01	0.07	0.06
VC0429	0.04	0.04	0.05	0.06
VC0430	0.04	0.02	0.04	0.04
VC0431	0.03	0.02	0.05	0.07
VC0432	0.03	0.03	0.05	0.08
VC0433	0.00	0.02	0.10	0.12
VC0435	0.14	0.13	0.01	0.01
VC0436	0.04	0.05	0.03	0.04
VC0437	0.06	0.05	0.05	0.05
VC0439	0.04	0.05	0.07	0.08
VC0440	0.07	0.07	0.08	0.09
VC0441	0.03	0.04	0.05	0.04
VC0442	0.06	0.07	0.04	0.03
VC0443	0.03	0.04	0.07	0.06
VC0444	0.03	0.03	0.05	0.05
VC0445	0.03	0.03	0.07	0.07
VC0446	0.03	0.04	0.04	0.04
VC0447	0.03	0.02	0.02	0.02
VC0448	0.02	0.03	0.04	0.06
VC0449	0.04	0.04	0.06	0.05
VC0450	0.06	0.06	0.07	0.06
VC0451	0.07	0.08	0.06	0.04
VC0452	0.03	0.04	0.11	0.07
VC0453	0.02	0.02	0.12	0.10
VC0454	0.03	0.03	0.04	0.04
VC0455	0.05	0.05	0.06	0.05
VC0456	0.03	0.04	0.13	0.12
VC0457	0.03	0.03	0.09	0.07
VC0458	0.02	0.02	0.05	0.05
VC0458	0.00	0.05	0.04	0.03
VC0459	0.03	0.02	0.14	0.13
VC0460	0.04	0.04	0.05	0.05
VC0461	0.05	0.06	0.07	0.06
VC0462	0.03	0.04	0.08	0.08
VC0463	0.02	0.02	0.05	0.04
VC0464	0.07	0.06	0.06	0.07
VC0465	0.02	0.05	0.07	0.07
VC0466	0.03	0.04	0.07	0.08
VC0467	0.02	0.02	0.03	0.03
VC0468	0.00	0.00	0.07	0.05
VC0469	0.06	0.06	0.08	0.07
VC0470	0.02	0.02	0.03	0.02
VC0471	0.02	0.02	0.07	0.09

VC0472	0.02	0.02	0.01	0.01
VC0473	0.04	0.05	0.03	0.04
VC0474	0.02	0.02	0.06	0.06
VC0475	0.01	0.00	0.02	0.02
VC0476	0.01	0.03	0.03	0.08
VC0477	0.02	0.03	0.04	0.03
VC0477	0.01	0.02	0.03	0.04
VC0478	0.02	0.04	0.05	0.06
VC0479	0.03	0.04	0.04	0.03
VC0480	0.00	0.01	0.01	0.02
VC0481	0.04	0.04	0.03	0.02
VC0482	0.05	0.05	0.05	0.05
VC0483	0.02	0.02	0.04	0.09
VC0484	0.04	0.03	0.05	0.05
VC0485	0.02	0.01	0.05	0.06
VC0486	0.05	0.07	0.07	0.06
VC0487	0.09	0.12	0.03	0.05
VC0488	0.01	0.03	0.05	0.07
VC0489	0.06	0.07	0.02	0.03
VC0490	0.03	0.02	0.05	0.05
VC0491	0.05	0.05	0.03	0.03
VC0492	0.02	0.02	0.04	0.05
VC0492	0.01	0.01	0.04	0.04
VC0493	0.04	0.05	0.04	0.05
VC0494	0.02	0.02	0.08	0.08
VC0495	0.02	0.02	0.06	0.05
VC0496	0.02	0.03	0.05	0.05
VC0497	0.04	0.05	0.04	0.05
VC0498	0.07	0.06	0.05	0.05
VC0499	0.03	0.04	0.07	0.09
VC0500	0.04	0.06	0.04	0.05
VC0501	0.03	0.04	0.06	0.06
VC0502	0.03	0.03	0.09	0.04
VC0503	0.02	0.02	0.02	0.02
VC0504	0.02	0.06	0.03	0.05
VC0505	0.06	0.06	0.06	0.06
VC0506	0.08	0.08	0.06	0.04
VC0507	0.07	0.08	0.01	0.03
VC0508	0.05	0.06	0.09	0.08
VC0509	0.05	0.06	0.08	0.07
VC0510	0.00	0.01	0.07	0.04
VC0511	0.05	0.05	0.05	0.04
VC0512	0.04	0.05	0.08	0.05

VC0513	0.01	0.01	0.09	0.08
VC0514	0.04	0.03	0.05	0.07
VC0515	0.01	0.01	0.02	0.02
VC0516	0.01	0.03	0.06	0.06
VC0517	0.07	0.05	0.04	0.06
VC0518	0.02	0.04	0.04	0.05
VC0519	0.05	0.06	0.08	0.09
VC0520	0.05	0.06	0.04	0.05
VC0521	0.09	0.07	0.06	0.06
VC0522	0.05	0.07	0.04	0.03
VC0523	-0.01	0.00	0.07	0.06
VC0524	0.05	0.06	0.09	0.10
VC0525	0.00	0.01	0.10	0.08
VC0526	0.03	0.03	0.04	0.05
VC0527	0.06	0.05	0.01	0.02
VC0528	-0.01	-0.01	0.12	0.13
VC0529	0.02	0.02	0.06	0.05
VC0530	0.06	0.07	0.06	0.04
VC0531	0.06	0.05	0.04	0.03
VC0532	0.00	0.00	0.09	0.10
VC0533	0.02	0.03	0.02	0.01
VC0534	0.00	0.00	0.08	0.07
VC0535	0.05	0.05	0.07	0.05
VC0536	0.05	0.05	0.06	0.07
VC0537	-0.01	-0.01	0.07	0.06
VC0538	0.06	0.05	0.06	0.05
VC0539	-0.01	0.03	0.04	0.04
VC0540	0.03	0.03	0.03	0.03
VC0541	0.02	0.02	0.03	0.06
VC0542	0.06	0.05	0.07	0.06
VC0543	0.04	0.03	0.07	0.07
VC0544	0.04	0.04	0.06	0.05
VC0545	0.10	0.10	0.06	0.06
VC0546	0.05	0.06	0.03	0.02
VC0547	0.03	0.04	0.05	0.05
VC0548	0.11	0.06	0.06	0.03
VC0549	0.06	0.06	0.02	0.02
VC0550	0.02	0.01	0.06	0.05
VC0551	0.01	0.03	0.02	0.02
VC0552	0.02	0.06	0.03	0.04
VC0553	0.04	0.04	0.07	0.06
VC0554	0.07	0.05	0.08	0.10
VC0555	0.03	0.03	0.02	0.02

VC0556	0.04	0.05	0.07	0.05
VC0557	0.04	0.04	0.06	0.08
VC0558	0.03	0.02	0.04	0.18
VC0559	0.00	0.01	0.03	0.03
VC0560	0.07	0.05	0.07	0.07
VC0561	0.03	0.03	0.02	0.03
VC0562	0.04	0.03	0.06	0.07
VC0563	0.03	0.04	0.07	0.07
VC0564	0.04	0.10	0.04	0.05
VC0565	0.02	0.05	0.04	0.04
VC0567	0.05	0.04	0.05	0.22
VC0568	0.05	0.05	0.05	0.04
VC0569	0.07	0.07	0.04	0.03
VC0570	0.06	0.07	0.07	0.03
VC0571	0.10	0.10	0.05	0.04
VC0572	0.08	0.08	0.01	0.02
VC0573	0.02	0.02	0.03	0.04
VC0574	0.08	0.07	0.03	0.03
VC0575	0.00	0.02	0.05	0.16
VC0576	0.08	0.06	0.04	0.02
VC0577	0.00	0.01	0.01	0.05
VC0578	0.03	0.03	0.01	0.01
VC0579	0.05	0.06	0.03	0.04
VC0580	0.07	0.07	0.08	0.05
VC0581	0.04	0.04	0.05	0.04
VC0582	0.02	0.03	0.03	0.04
VC0583	0.09	0.07	0.02	0.03
VC0584	0.05	0.06	0.05	0.04
VC0585	0.07	0.06	0.05	0.06
VC0586	0.07	0.06	0.01	0.02
VC0587	0.04	0.04	0.03	0.07
VC0588	0.07	0.06	0.04	0.05
VC0589	0.04	0.04	0.04	0.07
VC0590	0.04	0.05	0.03	0.04
VC0591	0.03	0.04	0.03	0.07
VC0592	0.01	0.01	0.08	0.05
VC0593	0.03	0.05	0.08	0.08
VC0596	0.07	0.07	0.08	0.06
VC0597	0.03	0.04	0.05	0.06
VC0598	0.04	0.04	0.03	0.04
VC0599	0.07	0.06	0.04	0.05
VC0600	0.06	0.08	0.03	0.03
VC0601	0.01	0.01	0.05	0.04

VC0602	0.01	0.02	0.04	0.04
VC0603	0.02	0.02	0.04	0.02
VC0604	0.03	0.04	0.05	0.06
VC0605	0.09	0.08	0.05	0.07
VC0606	0.07	0.07	0.05	0.04
VC0607	0.03	0.04	0.00	0.04
VC0607	0.01	0.00	0.07	0.07
VC0609	0.02	0.04	0.02	0.02
VC0610	0.02	0.02	0.04	0.04
VC0611	0.02	0.03	0.02	0.05
VC0611	0.01	0.02	0.05	0.05
VC0612	0.00	0.01	0.03	0.04
VC0613	0.05	0.07	0.03	0.06
VC0614	0.04	0.03	0.12	0.12
VC0615	0.02	0.02	0.04	0.06
VC0615	-0.01	0.07	0.06	0.05
VC0616	0.02	0.01	0.04	0.06
VC0617	0.00	0.03	0.04	0.05
VC0619	0.03	0.04	0.06	0.04
VC0620	0.02	0.03	0.02	0.04
VC0620	0.01	0.04	0.03	0.04
VC0621	0.07	0.08	0.06	0.05
VC0622	0.02	0.03	0.04	0.04
VC0623	0.03	0.03	0.05	0.04
VC0624	0.06	0.05	0.10	0.10
VC0625	0.04	0.07	0.05	0.05
VC0626	0.08	0.06	0.07	0.06
VC0627	0.05	0.06	0.05	0.05
VC0628	0.05	0.09	0.05	0.05
VC0629	0.02	0.04	0.04	0.04
VC0630	0.08	0.06	0.07	0.06
VC0631	0.07	0.05	0.11	0.08
VC0632	0.03	0.03	0.05	0.05
VC0633	0.06	0.05	0.09	0.06
VC0634	0.07	0.16	0.08	0.10
VC0635	0.06	0.05	0.12	0.11
VC0636	0.06	0.07	0.09	0.08
VC0637	0.10	0.08	0.06	0.09
VC0638	0.03	0.04	0.08	0.04
VC0639	0.06	0.06	0.08	0.09
VC0640	0.06	0.05	0.09	0.06
VC0641	0.08	0.09	0.05	0.05
VC0642	0.04	0.05	0.01	0.01

VC0643	0.10	0.10	0.04	0.06
VC0644	0.01	0.02	0.01	0.02
VC0645	0.08	0.06	0.16	0.20
VC0646	0.08	0.06	0.03	0.04
VC0647	0.08	0.08	0.05	0.06
VC0648	0.04	0.04	0.08	0.07
VC0649	0.05	0.04	0.06	0.05
VC0650	0.04	0.05	0.06	0.06
VC0651	0.05	0.04	0.08	0.07
VC0652	0.07	0.06	0.07	0.07
VC0653	-0.02	-0.01	-0.01	-0.01
VC0654	0.04	0.06	0.07	0.06
VC0655	0.03	0.03	0.01	0.02
VC0656	0.03	0.03	0.07	0.05
VC0657	0.06	0.06	0.06	0.05
VC0658	0.60	0.64	0.58	0.54
VC0659	0.06	0.05	0.05	0.06
VC0660	0.04	0.05	0.03	0.03
VC0661	0.07	0.07	0.14	0.13
VC0662	0.04	0.05	0.12	0.15
VC0663	0.07	0.06	0.09	0.10
VC0664	0.04	0.03	0.06	0.06
VC0665	0.12	0.13	0.15	0.13
VC0666	0.04	0.04	0.07	0.09
VC0667	0.03	0.03	0.03	0.04
VC0668	0.06	0.06	0.08	0.08
VC0669	0.06	0.06	0.05	0.04
VC0670	0.08	0.08	0.01	0.03
VC0671	0.04	0.05	0.05	0.05
VC0672	0.02	0.02	0.03	0.04
VC0673	0.04	0.04	0.06	0.07
VC0674	0.05	0.05	0.09	0.09
VC0675	0.01	0.01	0.00	0.01
VC0676	0.08	0.09	0.05	0.08
VC0677	0.06	0.06	0.09	0.08
VC0678	0.02	0.04	0.07	0.07
VC0679	0.03	0.06	0.05	0.04
VC0680	0.04	0.03	0.06	0.05
VC0681	0.01	0.01	0.00	0.01
VC0682	0.11	0.14	0.03	0.08
VC0683	0.05	0.04	0.08	0.07
VC0684	0.04	0.04	0.10	0.09
VC0685	0.04	0.05	0.09	0.07

VC0686	0.07	0.09	0.05	0.06
VC0687	0.04	0.05	0.05	0.06
VC0688	0.05	0.04	0.06	0.06
VC0689	0.08	0.07	0.04	0.03
VC0690	0.08	0.07	0.13	0.08
VC0691	0.05	0.06	0.08	0.07
VC0692	0.03	0.07	0.13	0.09
VC0693	0.09	0.08	0.04	0.04
VC0694	0.05	0.09	0.03	0.05
VC0695	0.06	0.06	0.12	0.09
VC0696	0.07	0.05	0.16	0.14
VC0697	0.04	0.04	0.07	0.06
VC0698	0.07	0.09	0.03	0.09
VC0699	0.09	0.14	0.09	0.07
VC0700	0.06	0.05	0.03	0.05
VC0701	0.08	0.07	0.10	0.11
VC0702	0.03	0.04	0.02	0.03
VC0703	0.07	0.07	0.15	0.14
VC0703	0.00	0.04	0.04	0.03
VC0704	0.02	0.02	0.05	0.05
VC0705	0.04	0.04	0.05	0.05
VC0706	0.03	0.04	0.04	0.04
VC0707	0.06	0.07	0.03	0.03
VC0708	0.04	0.03	0.07	0.05
VC0709	0.07	0.06	0.05	0.06
VC0710	0.05	0.04	0.08	0.06
VC0711	0.05	0.07	0.03	0.03
VC0712	0.05	0.06	0.06	0.06
VC0713	0.03	0.03	0.07	0.05
VC0714	0.02	0.00	0.00	0.02
VC0715	0.06	0.07	0.04	0.06
VC0716	0.02	0.02	0.00	0.01
VC0717	0.04	0.04	0.02	0.02
VC0718	0.04	0.05	0.06	0.06
VC0719	0.09	0.08	0.03	0.05
VC0720	0.02	0.02	0.01	0.02
VC0721	0.02	0.02	0.04	0.01
VC0722	0.07	0.05	0.05	0.04
VC0723	0.03	0.02	0.06	0.06
VC0724	0.01	0.01	0.07	0.07
VC0725	-0.03	0.01	0.09	0.07
VC0726	0.08	0.08	0.06	0.06
VC0727	0.05	0.07	0.06	0.06

VC0728	0.02	0.03	0.04	0.04
VC0729	0.07	0.07	0.04	0.04
VC0729	0.06	0.06	0.04	0.04
VC0730	0.13	0.10	0.06	0.06
VC0731	0.17	0.14	0.02	0.04
VC0732	0.09	0.08	0.04	0.05
VC0733	0.05	0.05	0.03	0.03
VC0734	0.00	0.02	0.06	0.04
VC0735	0.06	0.08	0.07	0.08
VC0736	0.17	0.17	0.04	0.02
VC0737	0.08	0.09	0.05	0.06
VC0738	0.06	0.08	0.08	0.08
VC0738	0.07	0.09	0.06	0.06
VC0739	0.15	0.13	0.03	0.03
VC0740	0.05	0.05	0.06	0.06
VC0741	0.03	0.05	0.05	0.05
VC0742	0.11	0.10	0.07	0.06
VC0743	0.04	0.06	0.03	0.04
VC0744	0.03	0.03	0.01	0.02
VC0745	0.15	0.11	0.01	0.00
VC0746	0.13	0.12	0.02	0.01
VC0747	0.09	0.08	0.01	0.02
VC0748	0.15	0.14	0.02	0.01
VC0749	0.10	0.09	0.00	0.01
VC0750	0.08	0.05	0.06	0.06
VC0751	0.07	0.07	0.03	0.04
VC0752	0.07	0.08	0.05	0.05
VC0753	0.10	0.10	0.01	0.04
VC0754	0.09	0.08	0.05	0.05
VC0755	0.11	0.09	0.04	0.04
VC0756	0.05	0.05	0.02	0.03
VC0757	0.14	0.15	0.03	0.03
VC0758	0.07	0.04	0.03	0.02
VC0759	0.09	0.07	0.00	0.00
VC0760	0.04	0.05	0.09	0.09
VC0761	0.12	0.09	0.01	0.00
VC0762	0.10	0.07	0.05	0.05
VC0763	0.00	0.02	0.01	0.01
VC0764	0.02	0.03	0.04	0.05
VC0765	0.13	0.11	0.03	0.01
VC0766	0.05	0.06	0.08	0.06
VC0767	-0.01	0.02	0.05	0.05
VC0768	0.01	0.01	0.07	0.05

VC0769	0.01	0.02	0.04	0.04
VC0770	0.07	0.10	0.01	0.00
VC0771	0.02	0.02	0.06	0.01
VC0772	-0.03	-0.03	0.07	0.06
VC0773	0.06	0.06	0.07	0.06
VC0774	0.06	0.08	0.03	0.04
VC0775	0.13	0.14	0.00	0.01
VC0776	0.02	0.03	0.07	0.02
VC0777	0.11	0.10	0.01	0.02
VC0778	0.12	0.11	0.02	-0.01
VC0779	0.03	0.04	0.05	0.05
VC0780	0.13	0.11	0.00	0.01
VC0781	0.12	0.11	0.00	0.01
VC0782	0.05	0.05	0.10	0.07
VC0783	0.06	0.05	0.07	0.06
VC0784	0.10	0.11	0.03	0.03
VC0785	0.05	0.05	0.04	0.05
VC0786	0.06	0.06	0.06	0.09
VC0787	0.03	0.04	0.06	0.06
VC0788	0.05	0.04	0.04	0.04
VC0788	0.02	0.01	0.03	0.04
VC0789	0.06	0.05	0.08	0.05
VC0790	0.05	0.03	0.09	0.07
VC0791	0.08	0.09	0.04	0.04
VC0792	0.02	0.03	0.08	0.08
VC0793	0.01	0.14	0.02	0.03
VC0793m	0.04	0.04	0.06	0.06
VC0794	0.10	0.11	0.01	0.02
VC0795	0.03	0.05	0.04	0.05
VC0796	0.05	0.06	0.06	0.07
VC0797	0.07	0.06	0.07	0.06
VC0798	0.05	0.04	0.03	0.04
VC0799	0.02	0.02	0.04	0.04
VC0800	0.08	0.07	0.12	0.12
VC0801	0.05	0.04	0.08	0.06
VC0802	0.05	0.05	0.07	0.06
VC0803	0.03	0.02	0.03	0.04
VC0805	0.02	0.02	0.04	0.05
VC0806	0.06	0.04	0.05	0.04
VC0807	0.02	0.02	0.10	0.06
VC0808	0.03	0.03	0.05	0.06
VC0809	0.04	0.05	0.06	0.04
VC0810	0.05	0.03	0.10	0.08

VC0811	0.05	0.05	0.07	0.07
VC0812	0.03	0.04	0.08	0.08
VC0813	0.06	0.07	0.06	0.06
VC0814	0.02	0.02	0.15	0.11
VC0815	0.22	0.24	0.01	0.01
VC0816	0.09	0.08	0.04	0.03
VC0817	0.03	0.06	0.08	0.07
VC0818	0.07	0.06	0.05	0.05
VC0819	0.02	0.02	0.07	0.06
VC0820	0.03	0.03	0.01	0.01
VC0821	0.02	0.04	0.05	0.04
VC0822	0.01	0.02	0.06	0.07
VC0823	0.02	0.02	0.05	0.05
VC0824	0.02	0.02	0.03	0.04
VC0824	0.01	0.02	0.06	0.05
VC0825	0.05	0.05	0.06	0.06
VC0826	0.04	0.05	0.06	0.05
VC0827	0.02	0.02	0.07	0.07
VC0827	0.01	-0.02	0.05	0.07
VC0828	0.01	0.01	0.04	0.03
VC0829	0.08	0.06	0.03	0.04
VC0830	0.06	0.07	0.07	-0.07
VC0831	0.05	0.05	0.06	0.04
VC0832	0.01	0.02	0.04	0.04
VC0833	0.04	0.05	0.07	0.07
VC0834	0.01	0.02	0.06	0.08
VC0835	0.06	0.06	0.07	0.06
VC0836	0.03	0.04	0.03	0.05
VC0837	0.04	0.06	0.11	0.12
VC0838	0.03	0.04	0.03	0.03
VC0839	0.03	0.04	0.05	0.04
VC0840	0.01	0.04	0.06	0.04
VC0841	0.01	0.01	0.01	0.02
VC0842	0.01	0.02	0.05	0.08
VC0843	0.02	0.03	0.04	0.04
VC0846	0.01	0.02	0.07	0.09
VC0847	0.05	0.05	0.05	0.04
VC0848	0.01	0.01	0.08	0.09
VC0849	-0.01	0.01	0.02	0.04
VC0850	0.07	0.06	0.06	0.10
VC0851	0.02	0.05	0.04	0.06
VC0852	0.06	0.07	0.11	0.13
VC0853	0.05	0.06	0.07	0.07

VC0854	0.08	0.07	0.04	0.05
VC0855	0.04	0.04	0.10	0.08
VC0856	0.03	0.06	0.04	0.04
VC0857	0.06	0.07	0.05	0.04
VC0858	0.03	0.02	0.05	0.04
VC0859	0.03	0.04	0.06	0.05
VC0860	0.04	0.04	0.04	0.04
VC0861	0.04	0.10	0.08	0.07
VC0862	0.05	0.05	0.10	0.06
VC0863	0.03	0.03	0.08	0.05
VC0864	0.05	0.07	0.07	0.08
VC0865	0.06	0.07	0.03	0.03
VC0866	0.00	0.00	0.03	0.02
VC0867	0.05	0.05	0.02	0.02
VC0868	0.04	0.03	0.06	0.06
VC0869	0.07	0.08	0.09	0.07
VC0870	0.01	0.03	0.05	0.04
VC0871	0.10	0.09	0.04	0.03
VC0872	0.08	0.08	0.03	0.03
VC0873	0.02	0.03	0.06	0.08
VC0874	0.03	0.03	0.07	0.04
VC0875	0.06	0.06	0.01	0.03
VC0876	0.02	0.02	0.07	0.10
VC0877	0.10	0.09	0.04	0.05
VC0878	0.07	0.08	0.06	0.08
VC0879	0.07	0.07	0.03	0.03
VC0880	0.04	0.03	0.04	0.05
VC0881	0.03	0.03	0.08	0.06
VC0882	0.06	0.06	0.02	0.03
VC0883	0.04	0.04	0.04	0.04
VC0884	0.01	0.02	0.02	0.04
VC0885	0.05	0.05	0.02	0.02
VC0886	0.04	0.01	0.03	0.05
VC0887	0.04	0.02	0.03	0.04
VC0888	0.09	0.06	0.05	0.07
VC0889	0.03	0.04	0.05	0.04
VC0890	0.03	0.04	0.01	0.01
VC0891	0.05	0.06	0.04	0.05
VC0892	0.03	0.03	0.05	0.03
VC0893	0.04	0.04	0.03	0.04
VC0894	0.01	0.02	0.07	0.06
VC0895	0.02	0.04	0.05	0.05
VC0897	0.03	0.03	0.12	0.09

VC0898	0.07	0.08	0.06	0.07
VC0899	0.00	0.02	0.11	0.08
VC0900	0.04	0.04	0.04	0.04
VC0901	0.06	0.06	0.06	0.08
VC0902	0.05	0.04	0.07	0.05
VC0903	0.14	0.06	0.01	0.03
VC0904	0.02	0.01	0.10	0.08
VC0905	0.04	0.04	0.09	0.09
VC0906	0.02	0.02	0.15	0.14
VC0907	0.06	0.07	0.05	0.05
VC0908	0.04	0.06	0.05	0.03
VC0909	0.03	0.02	0.04	0.06
VC0910	0.02	0.02	0.11	0.16
VC0911	0.08	0.09	0.05	0.04
VC0912	0.02	0.04	0.04	0.05
VC0914	0.04	0.05	0.06	0.05
VC0915	0.05	0.05	0.06	0.04
VC0916	0.05	0.05	0.05	0.05
VC0917	0.04	0.04	0.06	0.06
VC0918	0.05	0.06	0.11	0.12
VC0919	0.07	0.08	0.04	0.03
VC0920	0.03	0.03	0.14	0.12
VC0921	0.04	0.05	0.18	0.16
VC0922	0.02	0.01	0.03	0.08
VC0923	0.04	0.05	0.00	0.04
VC0924	0.04	0.01	0.03	0.04
VC0925	0.05	0.06	0.04	0.04
VC0926	0.01	0.03	0.04	0.03
VC0927	0.02	0.01	0.04	0.03
VC0928	0.03	0.05	0.05	0.03
VC0929	0.04	0.02	0.03	0.03
VC0930	0.06	0.05	0.06	0.06
VC0931	0.01	0.02	0.04	0.04
VC0932	0.01	0.01	0.03	0.04
VC0932	0.01	0.02	0.03	0.02
VC0934	0.03	0.01	0.05	0.05
VC0935	0.02	0.03	0.03	0.05
VC0936	0.03	0.04	0.02	0.03
VC0937	0.02	0.04	0.07	0.06
VC0938	0.01	0.00	0.07	0.05
VC0939	0.06	0.05	0.01	0.02
VC0940	0.06	0.06	0.01	0.01
VC0941	0.05	0.06	0.03	0.03

VC0943	0.02	0.01	0.03	0.02
VC0944	0.04	0.05	0.09	0.09
VC0945	0.04	0.06	0.05	0.05
VC0946	0.05	0.05	0.05	0.05
VC0947	0.00	0.00	0.10	0.08
VC0948	0.03	0.03	0.10	0.09
VC0949	0.02	0.05	0.03	0.03
VC0950	0.03	0.03	0.03	0.03
VC0951	0.04	0.04	0.08	0.07
VC0952	0.03	0.01	0.11	0.10
VC0953	0.00	0.01	0.00	0.01
VC0954	0.03	0.04	0.03	0.03
VC0955	0.02	0.02	0.04	0.06
VC0957	0.07	0.06	0.04	0.07
VC0958	0.00	0.04	0.02	0.02
VC0959	0.03	0.02	0.08	0.06
VC0960	0.04	0.05	0.06	0.07
VC0961	0.05	0.06	0.05	0.05
VC0962	0.04	0.04	0.07	0.06
VC0963	0.04	0.05	0.06	0.06
VC0964	0.07	0.05	0.08	0.06
VC0965	0.07	0.07	0.06	0.05
VC0966	0.04	0.05	0.07	0.05
VC0967	-0.01	0.00	0.09	0.06
VC0968	0.00	0.02	0.06	0.05
VC0969	0.01	0.01	0.08	0.07
VC0970	-0.01	0.04	0.05	0.04
VC0972	-0.01	-0.01	0.09	0.03
VC0973	0.04	0.03	0.05	0.05
VC0974	0.07	0.05	0.05	0.05
VC0975	0.05	0.11	0.05	0.08
VC0976	-0.01	-0.01	0.07	0.04
VC0977	0.00	0.01	0.04	0.04
VC0978	0.05	0.06	0.05	0.04
VC0979	0.04	0.03	0.04	0.04
VC0980	0.00	0.00	0.07	0.07
VC0981	0.00	0.01	0.09	0.09
VC0982	0.05	0.04	0.08	0.06
VC0983	-0.01	-0.01	0.04	0.04
VC0984	0.01	0.03	0.05	0.05
VC0985	0.03	0.04	0.10	0.09
VC0986	0.05	0.07	0.06	0.08
VC0987	0.04	0.04	0.06	0.04

VC0988	0.00	0.00	0.06	0.05
VC0989	0.04	0.05	0.03	0.03
VC0990	0.00	-0.01	0.09	0.06
VC0991	0.01	0.00	0.08	0.08
VC0992	0.00	0.01	0.07	0.07
VC0993	-0.01	-0.01	0.06	0.06
VC0994	-0.02	-0.01	0.07	0.06
VC0995	0.13	0.10	0.05	0.07
VC0996	0.05	0.02	0.02	0.01
VC0997	0.06	0.09	0.04	0.05
VC0998	0.00	0.04	0.03	0.04
VC0999	0.05	0.04	0.06	0.05
VC1000	0.05	0.06	0.04	0.06
VC1001	0.04	0.04	0.06	0.07
VC1002	0.03	0.04	0.04	0.04
VC1003	0.02	0.03	0.06	0.05
VC1004	0.02	0.04	0.04	0.05
VC1005	0.03	0.03	0.05	0.05
VC1006	0.00	0.01	0.00	0.00
VC1007	0.03	0.05	0.06	0.05
VC1008	0.02	0.13	0.05	0.05
VC1009	0.04	0.05	0.05	0.04
VC1010	0.03	0.03	0.05	0.10
VC1011	0.00	0.03	0.06	0.05
VC1012	0.04	0.03	0.05	0.05
VC1013	0.08	0.02	0.02	0.04
VC1014	0.02	0.01	-0.01	-0.01
VC1015	0.05	0.07	-0.03	-0.01
VC1016	0.02	0.02	0.04	0.03
VC1017	0.04	0.03	0.04	0.05
VC1018	0.04	0.04	0.02	0.02
VC1020	0.10	0.07	0.05	0.07
VC1021	0.03	0.02	0.05	0.05
VC1022	-0.02	-0.02	0.06	0.01
VC1023	0.03	0.04	0.09	0.08
VC1024	0.01	0.03	0.02	0.02
VC1025	0.04	0.05	0.02	0.03
VC1026	0.09	0.09	0.02	0.05
VC1027	0.06	0.06	0.04	0.06
VC1028	0.02	0.03	0.04	0.04
VC1029	0.00	-0.01	-0.02	-0.01
VC1030	0.07	0.06	0.04	0.03
VC1031	0.04	0.06	0.03	0.03

VC1032	0.06	0.08	0.05	0.02
VC1033	0.03	0.04	0.03	0.01
VC1034	0.03	0.03	0.04	0.06
VC1035	0.07	0.05	0.04	0.04
VC1037	0.05	0.06	0.06	0.05
VC1038	0.09	0.07	0.07	0.09
VC1039	0.02	0.04	0.01	0.01
VC1040	0.04	0.04	0.06	0.05
VC1041	0.03	0.04	0.02	0.03
VC1043	0.04	0.03	0.04	0.04
VC1044	0.03	0.04	0.04	0.02
VC1045	0.03	0.03	-0.01	-0.01
VC1046	0.07	0.06	0.04	0.05
VC1047	0.03	0.03	0.06	0.04
VC1048	0.04	0.05	0.04	0.03
VC1049	0.06	0.06	0.07	0.07
VC1050	0.02	0.03	0.04	0.06
VC1051	0.08	0.07	0.03	0.05
VC1052	0.05	0.06	0.03	0.03
VC1053	0.09	0.08	0.03	0.05
VC1054	0.08	0.08	0.03	0.06
VC1055	0.13	0.14	0.07	0.09
VC1056	0.04	0.05	0.04	0.03
VC1057	0.05	0.05	0.04	0.05
VC1058	0.06	0.07	0.04	0.07
VC1059	0.05	0.05	0.04	0.03
VC1060	0.02	0.02	0.05	0.06
VC1061	0.01	0.01	0.06	0.06
VC1062	0.05	0.06	0.08	0.04
VC1063	0.08	0.11	0.08	0.07
VC1064	0.08	0.08	0.04	0.04
VC1065	0.10	0.09	0.03	0.02
VC1066	0.08	0.07	0.04	0.04
VC1067	0.02	0.01	-0.02	-0.02
VC1068	0.08	0.07	0.06	0.04
VC1069	0.02	0.04	0.05	0.05
VC1070	0.13	0.09	0.03	0.10
VC1071	0.06	0.08	0.04	0.06
VC1072	0.06	0.06	0.11	0.14
VC1074	0.07	0.05	0.07	0.06
VC1075	0.09	0.08	0.07	0.06
VC1077	0.06	0.09	0.02	0.02
VC1078	0.06	0.06	0.06	0.07

VC1079	0.02	0.02	0.04	0.05
VC1080	0.08	0.08	0.06	0.04
VC1081	0.08	0.09	0.06	0.05
VC1082	0.06	0.06	0.07	0.05
VC1083	0.04	0.06	0.07	0.09
VC1084	0.08	0.09	0.06	0.05
VC1085	0.07	0.07	0.08	0.07
VC1086	0.00	0.00	0.02	0.02
VC1087	0.05	0.05	0.04	0.06
VC1088	0.05	0.05	0.04	0.05
VC1089	0.07	0.07	0.05	0.05
VC1090	0.08	0.08	0.02	0.03
VC1091	0.04	0.05	0.05	0.06
VC1092	0.04	0.05	0.07	0.08
VC1093	0.05	0.07	0.04	0.06
VC1094	0.04	0.05	0.07	0.05
VC1095	0.05	0.04	0.06	0.06
VC1096	0.07	0.06	0.10	0.08
VC1097	0.02	0.02	0.06	0.06
VC1098	0.07	0.08	0.08	0.08
VC1099	0.05	0.04	0.12	0.07
VC1100	0.07	0.06	0.16	0.12
VC1101	0.06	0.05	0.09	0.08
VC1102	0.04	0.04	0.03	0.02
VC1103	0.04	0.05	0.03	0.03
VC1104	0.04	0.04	0.00	0.00
VC1105	0.02	0.03	0.03	0.05
VC1106	0.08	0.07	0.08	0.08
VC1107	0.03	0.04	0.05	0.04
VC1108	0.07	0.05	0.04	0.05
VC1109	0.04	0.06	0.06	0.03
VC1109	0.05	0.07	0.05	0.06
VC1110	0.03	0.05	0.05	0.04
VC1111	0.08	0.09	0.04	0.06
VC1112	0.10	0.07	0.05	0.05
VC1113	0.06	0.05	0.07	0.08
VC1114	0.06	0.05	0.06	0.08
VC1115	0.07	0.06	0.11	0.11
VC1116	0.04	0.04	0.04	0.04
VC1117	0.04	0.05	0.11	0.10
VC1118	0.04	0.04	0.04	0.05
VC1119	0.04	0.06	0.10	0.09
VC1120	0.05	0.04	0.03	0.05

VC1121	0.04	0.04	0.03	0.03
VC1122	0.06	0.06	0.05	0.06
VC1123	0.04	0.04	0.07	0.06
VC1124	0.00	0.04	0.07	0.08
VC1125	0.05	0.05	0.06	0.06
VC1126	0.05	0.08	0.08	0.09
VC1127	0.06	0.06	0.07	0.14
VC1128	0.02	0.02	0.01	0.02
VC1129	0.06	0.07	0.08	0.06
VC1130	0.07	0.07	0.08	0.07
VC1131	0.06	0.07	0.09	0.09
VC1132	0.03	0.04	0.10	0.11
VC1133	0.04	0.04	0.07	0.06
VC1134	0.03	0.03	0.05	0.06
VC1135	0.03	0.03	0.08	0.09
VC1136	0.04	0.03	0.05	0.05
VC1137	0.04	0.05	0.06	0.08
VC1138	0.06	0.05	0.08	0.09
VC1139	0.04	0.03	0.07	0.06
VC1140	0.06	0.06	0.12	0.07
VC1141	0.02	0.02	0.06	0.05
VC1142	0.05	0.05	0.05	0.09
VC1143	0.05	0.05	0.15	0.08
VC1144	0.02	0.04	0.04	0.04
VC1145	0.03	0.06	0.03	0.03
VC1146	0.06	0.05	0.09	0.11
VC1147	0.07	0.07	0.10	0.15
VC1148	0.05	0.06	0.02	0.04
VC1149	0.08	0.07	0.03	0.05
VC1150	0.03	0.03	0.09	0.08
VC1151	0.07	0.06	0.10	0.06
VC1152	0.07	0.06	0.11	0.06
VC1153	0.01	0.01	0.20	0.18
VC1154	0.05	0.05	0.06	0.05
VC1155	0.05	0.05	0.09	0.10
VC1156	0.03	0.03	0.03	0.03
VC1156	0.00	0.05	0.06	0.06
VC1158	0.00	0.00	0.07	0.07
VC1159	0.03	0.02	0.06	0.04
VC1160	0.07	0.05	0.05	0.04
VC1161	0.04	0.05	0.08	0.07
VC1162	0.07	0.07	0.06	0.07
VC1163	0.04	0.04	0.06	0.01

VC1164	0.08	0.09	0.05	0.05
VC1165	0.05	0.06	0.03	0.03
VC1165	0.05	0.04	0.01	0.05
VC1166	0.01	0.04	0.05	0.05
VC1167	0.04	0.04	0.04	0.04
VC1169	0.03	0.03	0.01	0.02
VC1170	0.07	0.07	0.08	0.06
VC1171	0.09	0.08	0.04	0.05
VC1172	0.02	0.02	0.01	0.02
VC1173	0.02	0.03	0.01	0.02
VC1174	0.07	0.07	0.09	0.08
VC1175	0.07	0.05	0.13	0.06
VC1176	0.04	0.04	0.04	0.07
VC1177	0.03	0.03	0.07	0.06
VC1178	0.02	0.03	0.00	0.01
VC1179	0.06	0.06	0.08	0.07
VC1180	0.07	0.07	0.04	0.04
VC1181	0.06	0.07	0.11	0.14
VC1182	0.03	0.03	0.02	0.03
VC1183	0.16	0.15	0.03	0.02
VC1184	0.13	0.11	0.03	0.04
VC1185	0.07	0.06	0.00	0.00
VC1186	0.09	0.10	0.02	0.02
VC1187	0.08	0.07	0.06	0.05
VC1188	0.04	0.03	0.05	0.07
VC1189	0.03	0.03	0.02	0.05
VC1189	0.01	0.01	0.05	0.06
VC1190	0.15	0.12	0.04	0.03
VC1191	0.10	0.10	0.02	0.04
VC1192	0.06	0.06	0.06	0.03
VC1193	0.05	0.10	0.06	0.05
VC1194	0.12	0.11	0.02	0.04
VC1195	0.10	0.11	0.07	0.10
VC1196	0.04	0.05	0.06	0.06
VC1197	0.04	0.05	0.07	0.08
VC1198	0.02	0.01	0.03	0.03
VC1199	0.12	0.11	0.02	0.01
VC1200	0.12	0.09	0.02	0.01
VC1201	0.07	0.07	0.02	0.04
VC1202	0.14	0.17	0.04	0.05
VC1203	0.09	0.06	-0.02	-0.02
VC1204	0.08	0.06	-0.02	-0.02
VC1205	0.09	0.08	0.00	0.00

VC1206	0.15	0.17	0.07	0.06
VC1207	0.07	0.05	0.01	0.00
VC1208	0.08	0.08	0.00	0.00
VC1209	0.12	0.09	0.03	0.02
VC1210	0.15	0.19	0.07	0.06
VC1211	-0.01	-0.01	-0.01	0.15
VC1212	0.05	0.05	0.04	0.05
VC1213	0.11	0.12	0.01	0.01
VC1214	0.05	0.03	0.03	0.05
VC1215	0.06	0.07	0.07	0.08
VC1216	0.13	0.15	0.02	0.03
VC1217	0.16	0.16	0.04	0.05
VC1218	0.06	0.06	0.04	0.04
VC1219	0.13	0.11	0.02	0.01
VC1220	0.07	0.07	0.04	0.04
VC1221	0.14	0.11	0.03	0.04
VC1222	0.11	0.10	0.03	0.05
VC1223	0.02	0.02	0.06	0.04
VC1223	0.04	0.05	0.06	0.06
VC1224	0.04	0.03	0.08	0.08
VC1225	0.08	0.08	0.05	0.06
VC1226	0.13	0.13	0.03	0.04
VC1227	0.10	0.09	0.07	0.07
VC1228	0.10	0.10	0.07	0.09
VC1229	0.07	0.07	0.04	0.05
VC1230	0.05	0.05	0.10	0.10
VC1230	0.07	0.09	0.06	0.04
VC1231	0.09	0.07	0.03	0.04
VC1232	0.03	0.05	0.06	0.06
VC1233	0.05	0.04	0.07	0.07
VC1234	0.04	0.04	0.06	0.05
VC1235	0.04	0.04	0.03	0.04
VC1236	0.06	0.08	0.09	0.10
VC1237	0.04	0.05	0.11	0.08
VC1238	0.08	0.07	0.09	0.08
VC1239	0.10	0.09	0.06	0.06
VC1240	0.10	0.06	0.08	0.06
VC1241	0.05	0.04	0.08	0.05
VC1242	0.08	0.06	0.06	0.08
VC1243	0.05	0.06	0.04	0.07
VC1244	0.03	0.03	0.05	0.05
VC1245	0.14	0.14	0.05	0.04
VC1246	0.11	0.08	0.04	0.05

VC1247	0.06	0.04	0.09	0.08
VC1248	0.05	0.05	0.05	0.04
VC1249	0.06	0.06	0.05	0.05
VC1250	0.12	0.12	0.18	0.06
VC1251	0.06	0.08	0.03	0.04
VC1252	0.08	0.06	0.08	0.09
VC1253	0.05	0.05	0.07	0.06
VC1254	0.05	0.05	0.06	0.05
VC1255	0.02	0.02	0.01	0.01
VC1256	0.09	0.09	0.04	0.04
VC1257	0.07	0.07	0.09	0.08
VC1258	0.02	0.02	0.05	0.05
VC1259	0.06	0.05	0.06	0.05
VC1260	0.03	0.03	0.08	0.07
VC1261	0.05	0.07	0.09	0.06
VC1262	0.06	0.04	0.07	0.06
VC1263	0.03	0.03	0.06	0.06
VC1265	0.06	0.07	0.03	0.06
VC1266	0.01	0.01	0.05	0.03
VC1267	0.00	0.01	0.06	0.05
VC1268	0.01	0.02	0.04	0.03
VC1269	0.04	0.04	0.06	0.05
VC1270	0.00	0.03	0.04	0.03
VC1271	0.01	0.02	0.05	0.04
VC1272	0.02	0.02	0.06	0.06
VC1273	0.04	0.04	0.06	0.09
VC1274	0.04	0.04	0.05	0.04
VC1275	0.02	0.02	0.04	0.03
VC1276	0.03	0.03	0.04	0.03
VC1277	0.04	0.03	0.07	0.06
VC1278	0.02	0.03	0.11	0.10
VC1279	0.04	0.05	0.03	0.03
VC1280	0.02	0.02	0.06	0.05
VC1281	0.06	0.05	0.04	0.03
VC1282	0.01	0.02	0.03	0.03
VC1283	0.09	0.09	0.03	0.03
VC1284	0.03	0.03	0.04	0.03
VC1285	0.03	0.03	0.06	0.05
VC1286	0.01	0.02	0.06	0.04
VC1287	0.02	0.02	0.05	0.05
VC1288	0.03	0.04	0.05	0.05
VC1289	0.01	0.02	0.05	0.05
VC1290	0.03	0.03	0.08	0.07

VC1291	0.03	0.03	0.06	0.05
VC1292	0.07	0.08	0.07	0.07
VC1293	0.05	0.05	0.06	0.06
VC1294	0.05	0.06	0.04	0.06
VC1295	0.46	0.47	0.48	0.54
VC1296	0.04	0.05	0.05	0.06
VC1297	0.01	0.02	0.00	0.01
VC1298	0.09	0.08	0.03	0.03
VC1299	0.05	0.04	0.07	0.07
VC1300	0.03	0.03	0.05	0.06
VC1301	0.13	0.12	0.08	0.08
VC1302	0.07	0.07	0.07	0.06
VC1303	0.04	0.05	0.05	0.08
VC1304	0.08	0.07	0.06	0.06
VC1305	0.03	0.03	0.03	0.04
VC1306	0.01	0.02	0.04	0.07
VC1307	0.02	0.06	0.02	0.03
VC1308	0.18	0.17	0.09	0.09
VC1309	0.10	0.07	0.05	0.08
VC1310	0.06	0.04	0.06	0.07
VC1311	0.03	0.03	0.06	0.05
VC1312	0.04	0.05	0.06	0.10
VC1313	0.09	0.05	0.05	0.05
VC1314	0.05	0.05	0.06	0.05
VC1315	0.04	0.05	0.07	0.05
VC1316	0.09	0.06	0.07	0.08
VC1317	0.05	0.05	0.07	0.06
VC1319	0.00	0.04	0.05	0.06
VC1320	0.03	0.03	0.03	0.03
VC1321	0.02	0.02	0.03	0.05
VC1322	0.07	0.06	0.04	0.03
VC1323	0.04	0.04	0.07	0.06
VC1324	0.07	0.06	0.03	0.03
VC1325	0.05	0.05	0.09	0.08
VC1326	0.12	0.14	0.03	0.03
VC1327	0.02	0.03	0.04	0.04
VC1329	0.03	0.04	0.05	0.04
VC1330	0.10	0.08	0.05	0.04
VC1331	0.01	0.03	0.05	0.04
VC1332	0.02	0.02	0.02	0.05
VC1332	0.01	0.02	0.04	0.04
VC1333	0.03	0.02	0.07	0.11
VC1334	0.03	0.02	0.04	0.05

VC1335	0.02	0.03	0.06	0.07
VC1336	0.07	0.07	0.04	0.05
VC1337	0.06	0.06	0.04	0.04
VC1338	0.04	0.04	0.08	0.04
VC1339	0.04	0.04	0.06	0.05
VC1340	0.05	0.03	0.04	0.03
VC1341	0.05	0.05	0.10	0.08
VC1341	0.01	0.01	0.05	0.05
VC1342	0.02	0.01	0.04	0.04
VC1343	0.03	0.05	0.05	0.05
VC1344	0.08	0.07	0.07	0.07
VC1345	0.08	0.08	0.11	0.15
VC1346	0.05	0.03	0.06	0.06
VC1348	0.45	0.44	0.43	0.41
VC1349	0.10	0.12	0.03	0.03
VC1350	0.06	0.06	0.09	0.08
VC1351	0.04	0.04	0.06	0.05
VC1352	0.03	0.05	0.04	0.05
VC1353	0.01	0.01	0.01	0.00
VC1354	0.05	0.05	0.06	0.05
VC1355	0.06	0.06	0.04	0.05
VC1356	0.05	0.06	0.09	0.12
VC1357	0.07	0.08	0.06	0.02
VC1358	0.07	0.07	0.05	0.04
VC1359	0.04	0.05	0.10	0.07
VC1360	0.05	0.04	0.07	0.08
VC1361	0.04	0.05	0.07	0.07
VC1362	0.07	0.04	0.06	0.07
VC1363	0.02	0.06	0.06	0.07
VC1364	0.06	0.07	0.05	0.04
VC1365	0.07	0.08	0.04	0.06
VC1366	0.08	0.04	0.05	0.05
VC1367	0.02	0.05	0.06	0.07
VC1368	0.04	0.05	0.06	0.06
VC1369	0.03	0.03	0.06	0.04
VC1370	0.40	0.43	0.40	0.39
VC1371	0.05	0.04	0.04	0.05
VC1372	0.04	0.02	0.03	0.04
VC1373	0.07	0.07	0.07	0.06
VC1374	0.08	0.05	0.05	0.04
VC1375	0.06	0.05	0.05	0.07
VC1377	0.04	0.05	0.04	0.04
VC1378	0.04	0.05	0.07	0.07

VC1379	0.07	0.08	0.08	0.07
VC1380	0.05	0.05	0.05	0.05
VC1381	0.08	0.07	0.05	0.05
VC1382	-0.01	0.02	0.03	0.05
VC1382	0.02	0.04	0.05	0.04
VC1383	0.03	0.04	0.08	0.07
VC1384	0.02	0.03	0.03	0.03
VC1385	0.09	0.06	0.10	0.06
VC1386	0.07	0.04	0.06	0.05
VC1387	0.06	0.05	0.04	0.04
VC1388	0.06	0.05	0.08	0.07
VC1389	0.04	0.05	0.07	0.06
VC1390	0.06	0.06	0.05	0.04
VC1391	0.02	0.03	0.08	0.06
VC1392	0.05	0.06	0.04	0.06
VC1393	0.02	0.02	0.04	0.03
VC1394	0.08	0.13	0.06	0.05
VC1395	0.04	0.04	0.07	0.04
VC1396	0.07	0.06	0.04	0.04
VC1397	0.08	0.07	0.06	0.06
VC1398	0.07	0.07	0.03	0.04
VC1399	0.04	0.05	0.04	0.03
VC1400	0.05	0.06	0.05	0.05
VC1401	0.01	0.03	0.05	0.05
VC1402	0.02	0.02	0.04	0.04
VC1403	0.07	0.08	0.06	0.06
VC1404	0.09	0.08	0.02	0.03
VC1405	0.02	0.02	0.13	0.10
VC1406	0.07	0.06	0.14	0.14
VC1407	0.05	0.05	0.02	0.04
VC1408	0.01	0.02	0.04	0.04
VC1409	0.06	0.04	0.03	0.02
VC1410	0.06	0.05	0.05	0.05
VC1411	0.03	0.03	0.03	0.03
VC1412	0.09	0.07	0.06	0.08
VC1413	0.10	0.10	0.00	0.02
VC1414	0.02	0.02	0.05	0.03
VC1415	0.09	0.09	0.06	0.06
VC1416	0.04	0.06	0.04	0.05
VC1417	0.03	0.03	0.07	0.07
VC1418	0.01	0.02	0.01	0.01
VC1419	0.02	0.03	0.05	0.03
VC1420	0.02	0.02	0.02	0.03

VC1421	0.01	0.02	0.00	0.03
VC1422	0.08	0.05	0.06	0.06
VC1423	0.06	0.05	0.05	0.05
VC1424	0.03	0.04	0.05	0.04
VC1425	0.05	0.06	0.05	0.04
VC1426	0.06	0.06	0.06	0.05
VC1427	0.07	0.03	0.04	0.04
VC1428	0.02	0.02	0.04	0.08
VC1429	0.04	0.03	0.05	0.04
VC1430	0.02	0.04	0.03	0.00
VC1431	0.06	0.06	0.06	0.04
VC1432	0.01	0.03	0.06	0.05
VC1433	0.01	0.01	0.03	0.03
VC1434	0.04	0.07	0.05	0.06
VC1435	0.05	0.04	0.03	0.03
VC1436	0.04	0.04	0.04	0.03
VC1437	0.02	0.03	0.06	0.06
VC1438	0.06	0.05	0.06	0.05
VC1439	0.01	0.02	0.04	0.04
VC1440	0.06	0.05	0.06	0.04
VC1441	0.02	0.01	0.06	0.09
VC1442	-0.01	-0.01	0.07	0.05
VC1443	0.02	0.03	0.03	0.04
VC1444	0.03	0.03	0.02	0.04
VC1445	0.03	0.02	0.04	0.05
VC1446	0.09	0.06	0.04	0.05
VC1447	0.01	0.02	0.09	0.07
VC1448	0.03	0.05	0.03	0.05
VC1449	0.03	0.04	0.07	0.06
VC1450	0.03	0.04	0.04	0.03
VC1452	0.07	0.05	0.07	0.08
VC1453	0.03	0.05	0.06	0.05
VC1454	0.05	0.07	0.06	0.04
VC1455	0.04	0.04	0.07	0.06
VC1456	0.04	0.04	0.05	0.04
VC1457	0.07	0.08	0.05	0.05
VC1458	0.03	0.04	0.07	0.04
VC1459	0.06	0.07	0.04	0.03
VC1460	0.06	0.06	0.10	0.08
VC1461	0.09	0.10	0.04	0.04
VC1462	0.05	0.08	0.06	0.05
VC1463	0.07	0.07	0.05	0.05
VC1464	0.06	0.05	0.04	0.05

VC1465	0.03	0.01	0.08	0.08
VC1466	0.03	0.02	0.07	0.07
VC1467	0.04	0.05	0.02	0.02
VC1468	0.10	0.10	0.05	0.04
VC1469	-0.01	0.00	0.04	0.07
VC1470	0.07	0.08	0.09	0.08
VC1471	0.06	0.07	0.06	0.03
VC1472	0.04	0.04	0.07	0.06
VC1473	0.06	0.05	0.07	0.07
VC1474	0.09	0.10	0.00	0.05
VC1475	0.05	0.06	0.04	0.04
VC1476	0.00	0.01	0.03	0.05
VC1477	0.00	0.02	0.04	0.06
VC1478	0.06	0.08	0.05	0.05
VC1479	-0.01	0.00	0.04	0.07
VC1480	0.07	0.07	0.03	0.02
VC1481	0.04	0.02	0.04	0.06
VC1482	0.02	0.02	0.04	0.04
VC1483	0.03	0.03	0.07	0.06
VC1484	0.03	0.06	0.06	0.07
VC1485	0.03	0.05	0.05	0.04
VC1486	0.02	0.01	0.05	0.08
VC1487	0.00	0.02	0.04	0.04
VC1488	0.07	0.07	0.04	0.06
VC1489	0.09	0.09	0.03	0.04
VC1490	0.08	0.09	0.03	0.04
VC1491	0.00	0.04	0.09	0.10
VC1492	0.03	0.03	0.05	0.06
VC1492	0.01	0.03	0.04	0.04
VC1493	0.08	0.07	0.04	0.05
VC1494	0.04	0.06	0.07	0.06
VC1495	0.01	0.01	0.08	0.08
VC1496	0.05	0.04	0.04	0.04
VC1497	0.05	0.06	0.05	0.05
VC1498	0.05	0.04	0.02	0.02
VC1499	0.05	0.07	0.04	0.05
VC1500	0.04	0.06	0.05	0.05
VC1500	0.05	0.04	0.02	0.02
VC1501	0.07	0.06	0.03	0.08
VC1502	0.11	0.15	0.02	0.05
VC1503	0.06	0.04	0.05	0.07
VC1505	0.03	0.03	0.05	0.05
VC1506	0.05	0.07	0.06	0.07

VC1507	0.03	0.04	0.08	0.07
VC1508	0.05	0.06	0.02	0.05
VC1509	0.05	0.04	0.07	0.07
VC1510	0.03	0.05	0.07	0.06
VC1511	0.09	0.09	0.08	0.10
VC1512	0.05	0.05	0.08	0.09
VC1513	0.08	0.05	0.07	0.06
VC1514	0.04	0.04	0.05	0.07
VC1515	0.09	0.08	0.07	0.06
VC1516	0.06	0.05	0.07	0.04
VC1517	0.04	0.05	0.05	0.04
VC1518	0.05	0.12	0.07	0.10
VC1519	0.08	0.06	0.05	0.06
VC1520	0.05	0.05	0.04	0.05
VC1521	0.04	0.04	0.07	0.08
VC1523	0.12	0.08	0.04	0.04
VC1524	0.03	0.06	0.05	0.05
VC1525	0.07	0.07	0.03	0.06
VC1526	0.06	0.05	0.06	0.06
VC1527	0.12	0.12	0.05	0.06
VC1528	0.02	0.02	0.03	0.03
VC1529	0.03	0.03	0.06	0.04
VC1530	0.04	0.04	0.08	0.06
VC1531	0.04	0.06	0.08	0.08
VC1532	0.05	0.06	0.04	0.06
VC1533	0.04	0.05	0.03	0.03
VC1534	0.03	0.02	0.09	0.10
VC1535	0.02	0.01	0.04	0.03
VC1537	0.03	0.03	0.04	0.03
VC1538	0.04	0.06	0.05	0.06
VC1539	0.05	0.04	0.06	0.06
VC1540	0.00	0.04	0.04	0.04
VC1541	0.02	0.04	0.05	0.07
VC1542	0.03	0.02	0.05	0.04
VC1543	0.03	0.04	0.07	0.06
VC1544	0.03	0.03	0.03	0.03
VC1545	0.05	0.06	0.06	0.06
VC1546	0.02	0.04	0.02	0.03
VC1547	0.03	0.03	0.06	0.05
VC1548	0.02	0.02	0.05	0.04
VC1549	0.05	0.05	0.03	0.05
VC1550	0.02	0.03	0.07	0.06
VC1551	0.06	0.06	0.05	0.07

VC1552	0.03	0.05	0.09	0.11
VC1553	0.05	0.05	0.05	0.06
VC1554	0.09	0.09	0.07	0.05
VC1555	0.06	0.04	0.09	0.08
VC1556	0.04	0.03	0.06	0.06
VC1557	0.02	0.03	0.06	0.05
VC1558	0.04	0.05	0.05	0.05
VC1559	0.05	0.05	0.03	0.08
VC1560	0.08	0.06	0.08	0.11
VC1561	0.05	0.07	0.04	0.07
VC1562	0.03	0.04	0.05	0.05
VC1563	0.02	0.08	0.04	0.04
VC1565	0.08	0.11	0.07	0.07
VC1566	0.07	0.07	0.06	0.05
VC1567	0.13	0.14	0.07	0.08
VC1568	0.05	0.08	0.05	0.06
VC1569	0.06	0.06	0.04	0.05
VC1570	0.03	0.08	0.06	0.06
VC1571	0.05	0.05	0.04	0.03
VC1572	0.00	0.03	0.04	0.04
VC1573	0.06	0.08	0.06	0.06
VC1574	0.04	0.07	0.05	0.04
VC1575	0.08	0.11	0.05	0.05
VC1576	0.05	0.08	0.04	0.06
VC1577	0.04	0.06	0.07	0.06
VC1578	0.04	0.04	0.10	0.10
VC1579	0.00	0.02	0.04	0.04
VC1580	0.04	0.07	0.03	0.05
VC1581	0.08	0.07	0.03	0.03
VC1582	0.06	0.05	0.05	0.05
VC1583	0.08	0.09	0.04	0.04
VC1584	0.03	0.04	0.03	0.05
VC1585	0.12	0.12	0.04	0.06
VC1586	0.07	0.08	0.05	0.04
VC1586	0.06	0.07	0.04	0.05
VC1587	0.03	0.07	0.06	0.06
VC1588	0.07	0.08	0.09	0.07
VC1589	0.05	0.07	0.07	0.07
VC1590	0.06	0.08	0.04	0.03
VC1591	0.06	0.07	0.07	0.07
VC1592	0.00	0.01	0.00	0.00
VC1593	0.05	0.06	0.05	0.05
VC1595	0.07	0.07	0.06	0.06

VC1596	0.09	0.09	0.06	0.07
VC1597	0.05	0.04	0.07	0.05
VC1598	0.04	0.06	0.06	0.06
VC1599	-0.03	0.02	-0.01	-0.01
VC1600	0.02	0.08	0.10	0.10
VC1601	0.13	0.11	0.03	0.04
VC1602	0.04	0.05	0.07	0.07
VC1603	0.04	0.07	0.04	0.04
VC1604	0.07	0.06	0.05	0.07
VC1605	0.01	0.01	0.03	0.03
VC1606	0.04	0.05	0.07	0.08
VC1607	0.02	0.03	0.05	0.04
VC1608	0.05	0.04	0.06	0.05
VC1609	0.01	0.01	0.06	0.06
VC1610	0.04	0.05	0.07	0.07
VC1611	0.05	0.04	0.05	0.05
VC1612	0.02	0.02	0.07	0.06
VC1613	0.07	0.07	0.01	0.02
VC1614	0.03	0.03	0.07	0.05
VC1615	0.06	0.07	0.07	0.07
VC1616	0.07	0.08	0.05	0.06
VC1617	0.05	0.07	0.07	0.06
VC1618	0.05	0.04	0.05	0.08
VC1619	0.03	0.03	0.05	0.04
VC1621	0.03	0.01	0.05	0.08
VC1622	0.09	0.09	0.07	0.06
VC1623	0.06	0.05	0.05	0.04
VC1624	0.09	0.07	0.05	0.05
VC1625	0.03	0.06	0.05	0.05
VC1627	0.03	0.02	0.03	0.05
VC1628	0.05	0.04	0.03	0.02
VC1629	0.01	0.01	0.07	0.06
VC1630	0.04	0.04	0.05	0.04
VC1631	0.03	0.06	0.05	0.07
VC1632	0.10	0.09	0.06	0.06
VC1633	0.04	0.06	0.06	0.07
VC1634	0.05	0.05	0.05	0.05
VC1635	0.15	0.05	0.10	0.11
VC1636	0.07	0.09	0.04	0.07
VC1637	0.05	0.03	0.02	0.05
VC1638	0.02	0.03	0.06	0.08
VC1639	0.01	0.03	0.05	0.08
VC1640	0.05	0.06	0.06	0.05

VC1641	0.56	0.56	0.58	0.60
VC1642	0.07	0.05	0.09	0.11
VC1643	0.01	0.01	0.05	0.03
VC1644	0.01	0.02	0.04	0.07
VC1646	0.07	0.08	0.05	0.05
VC1647	0.02	0.01	0.04	0.03
VC1648	0.05	0.09	0.05	0.06
VC1648	0.06	0.06	0.01	0.04
VC1649	0.01	0.03	0.02	0.02
VC1651	0.01	0.01	0.04	0.04
VC1652	0.29	0.28	0.04	0.03
VC1653	0.06	0.06	0.05	0.06
VC1654	0.06	0.06	0.04	0.04
VC1656	0.05	0.06	0.04	0.04
VC1657	0.07	0.09	0.04	0.04
VC1658	0.07	0.11	0.06	0.05
VC1659	0.05	0.08	0.13	0.06
VC1660	0.05	0.06	0.07	0.05
VC1661	0.05	0.06	0.04	0.04
VC1662	0.05	0.06	0.06	0.07
VC1663	0.09	0.11	0.06	0.06
VC1664	0.04	0.12	0.07	0.07
VC1665	0.08	0.08	0.05	0.05
VC1666	0.04	0.05	0.04	0.05
VC1667	0.03	0.02	0.05	0.05
VC1668	0.03	0.04	0.06	0.06
VC1669	0.02	0.02	0.01	0.04
VC1670	0.06	0.06	0.05	0.06
VC1671	0.07	0.06	0.15	0.12
VC1672	0.06	0.05	0.12	0.09
VC1673	0.04	0.05	0.08	0.11
VC1674	0.06	0.06	0.12	0.09
VC1675	0.03	0.03	0.10	0.09
VC1676	0.06	0.07	0.09	0.10
VC1677	0.03	0.04	0.06	0.06
VC1678	0.08	0.07	0.15	0.15
VC1679	0.05	0.05	0.09	0.08
VC1680	0.05	0.04	0.05	0.03
VC1681	0.05	0.06	0.09	0.09
VC1682	0.06	0.06	0.12	0.10
VC1683	0.04	0.05	0.02	0.02
VC1684	0.05	0.06	0.09	0.09
VC1685	0.05	0.05	0.10	0.08

VC1686	0.11	0.12	0.09	0.05
VC1687	0.07	0.08	0.16	0.10
VC1688	0.09	0.05	0.05	0.06
VC1689	0.07	0.06	0.08	0.04
VC1690	0.04	0.04	0.06	0.05
VC1691	0.10	0.05	0.01	0.05
VC1692	0.03	0.04	0.06	0.04
VC1693	0.06	0.03	0.03	0.04
VC1695	0.07	0.06	0.13	0.09
VC1696	0.08	0.08	0.10	0.09
VC1697	0.07	0.08	0.15	0.11
VC1698	0.06	0.05	0.05	0.04
VC1699	0.04	0.05	0.02	0.06
VC1700	0.06	0.05	0.03	0.03
VC1701	0.04	0.05	0.05	0.05
VC1702	0.05	0.05	0.08	0.08
VC1703	0.03	0.03	0.09	0.06
VC1704	0.04	0.05	0.05	0.06
VC1705	0.03	0.03	0.04	0.03
VC1706	0.06	0.07	0.10	0.08
VC1707	0.07	0.07	0.12	0.12
VC1708	0.04	0.06	0.07	-0.05
VC1709	0.02	0.02	0.02	0.07
VC1710	0.34	0.30	0.30	0.26
VC1711	0.04	0.05	0.04	0.05
VC1712	0.02	0.02	0.06	0.04
VC1713	0.10	0.10	0.10	0.08
VC1714	0.01	0.02	0.06	0.05
VC1714	0.02	0.03	0.04	0.04
VC1715	0.07	0.06	0.05	0.03
VC1716	0.09	0.11	0.05	0.05
VC1717	0.06	0.07	0.07	0.13
VC1718	0.08	0.09	0.10	0.08
VC1719	0.01	0.03	0.05	0.04
VC1720	0.04	0.06	0.05	0.04
VC1721	0.05	0.06	0.07	0.07
VC1722	0.05	0.05	0.04	0.04
VC1724	0.06	0.06	0.06	0.05
VC1725	0.07	0.08	0.10	0.10
VC1726	0.02	0.02	0.02	0.02
VC1727	0.01	0.01	0.05	0.04
VC1728	0.04	0.06	0.02	0.04
VC1729	0.07	0.07	0.05	0.08

VC1730	0.02	0.02	0.03	0.04
VC1730	0.00	0.04	0.05	0.05
VC1731	0.03	0.04	0.06	0.05
VC1732	0.06	0.07	0.07	0.09
VC1733	0.05	0.08	0.07	0.06
VC1734	0.06	0.06	0.04	0.03
VC1735	0.07	0.08	0.05	0.04
VC1736	0.04	0.06	0.05	0.04
VC1737	0.05	0.06	0.07	0.07
VC1738	0.02	0.02	0.05	0.07
VC1740	0.05	0.04	0.04	0.03
VC1741	0.08	0.07	0.06	0.06
VC1742	0.04	0.05	0.09	0.06
VC1743	0.06	0.06	0.06	0.05
VC1744	0.06	0.09	0.07	0.06
VC1745	0.04	0.05	0.04	0.04
VC1746	0.05	0.07	0.05	0.08
VC1747	0.04	0.07	0.05	0.05
VC1748	0.06	0.07	0.05	0.04
VC1749	0.11	0.08	0.06	0.06
VC1750	0.15	0.05	0.02	0.02
VC1751	0.06	0.07	0.07	0.06
VC1752	0.06	0.07	0.04	0.03
VC1753	0.03	0.04	0.05	0.05
VC1754	0.06	0.07	0.06	0.06
VC1755	0.07	0.07	0.06	0.06
VC1756	0.04	0.04	0.07	0.05
VC1757	0.02	0.03	0.05	0.04
VC1758	0.05	0.08	0.06	0.06
VC1759	0.07	0.06	0.05	0.06
VC1760	0.02	0.04	0.04	0.04
VC1761	0.03	0.05	0.04	0.03
VC1762	0.06	0.09	0.06	0.05
VC1763	0.02	0.03	0.03	0.03
VC1764	0.02	0.03	0.02	0.03
VC1765	0.04	0.04	0.02	0.02
VC1766	0.08	0.10	0.04	0.05
VC1767	0.04	0.05	0.06	0.03
VC1768	0.06	0.07	0.07	0.08
VC1769	0.02	0.02	0.05	0.05
VC1770	0.03	0.03	0.05	0.04
VC1771	0.02	0.03	0.06	0.07
VC1772	0.05	0.06	0.07	0.07

VC1773	0.09	0.07	0.04	0.06
VC1774	0.06	0.07	0.09	0.10
VC1775	0.06	0.06	0.05	0.07
VC1776	0.04	0.05	0.06	0.06
VC1777	0.06	0.08	0.07	0.06
VC1778	0.05	0.05	0.06	0.06
VC1779	0.06	0.07	0.06	0.04
VC1780	0.04	0.06	0.08	0.08
VC1781	0.08	0.09	0.08	0.06
VC1782	0.06	0.08	0.07	0.07
VC1783	0.05	0.06	0.07	0.06
VC1784	0.04	0.06	0.05	0.03
VC1785	0.05	0.06	0.06	0.08
VC1786	0.06	0.08	0.06	0.07
VC1787	0.06	0.07	0.07	0.07
VC1788	0.07	0.07	0.04	0.05
VC1789	0.05	0.05	0.03	0.04
VC1790	0.11	0.08	0.04	0.06
VC1791	0.06	0.07	0.04	0.05
VC1792	0.09	0.08	0.07	0.05
VC1793	0.10	0.08	0.04	0.05
VC1794	0.08	0.08	0.10	0.11
VC1795	0.10	0.08	0.06	0.07
VC1796	0.08	0.06	0.04	0.06
VC1797	0.08	0.07	0.04	0.04
VC1798	0.01	0.01	-0.01	0.01
VC1799	0.10	0.12	0.05	0.09
VC1800	0.02	0.02	0.04	0.04
VC1801	0.01	0.01	0.09	0.08
VC1802	0.13	0.11	0.07	0.09
VC1803	0.10	0.09	0.04	0.05
VC1804	0.10	0.09	0.05	0.04
VC1805	0.09	0.07	0.05	0.07
VC1806	0.07	0.07	0.05	0.05
VC1807	0.01	0.04	0.05	0.03
VC1807	0.00	0.03	0.05	0.04
VC1808	0.09	0.08	0.05	0.06
VC1809	0.06	0.07	0.04	0.05
VC1810	0.08	0.07	0.05	0.05
VC1811	0.04	0.07	0.04	0.04
VC1812	0.06	0.08	0.06	0.07
VC1813	0.04	0.05	0.06	0.09
VC1814	0.06	0.07	0.09	0.07

VC1815	0.07	0.06	0.07	0.06
VC1816	0.03	0.05	0.07	0.08
VC1817	0.06	0.06	0.05	0.06
VC1818	0.02	0.04	0.06	0.05
VC1819	0.05	0.06	0.03	0.05
VC1820	0.11	0.08	0.08	0.10
VC1821	0.05	0.06	0.02	0.04
VC1822	0.08	0.06	0.08	0.05
VC1823	0.11	0.12	0.04	0.06
VC1824	0.10	0.11	0.07	0.05
VC1825	0.14	0.15	0.05	0.06
VC1826	0.04	0.04	0.04	0.06
VC1827	0.06	0.04	0.07	0.07
VC1828	0.09	0.09	0.07	0.05
VC1829	0.04	0.06	0.09	0.10
VC1830	0.05	0.05	0.06	0.04
VC1830	0.06	0.07	0.09	0.07
VC1831	0.03	0.04	0.05	0.06
VC1831	0.02	0.01	0.03	0.02
VC1832	0.03	0.02	0.08	0.05
VC1833	0.10	0.11	0.04	0.04
VC1834	0.08	0.07	0.05	0.04
VC1835	0.03	0.02	0.06	0.06
VC1836	0.06	0.06	0.06	0.06
VC1837	0.03	0.06	0.09	0.09
VC1838	0.09	0.08	0.07	0.07
VC1839	0.06	0.06	0.07	0.08
VC1840	0.06	0.04	0.05	0.05
VC1841	0.05	0.07	0.05	0.06
VC1842	0.04	0.05	0.04	0.03
VC1842	0.05	0.06	0.06	0.07
VC1843	0.01	0.01	0.00	0.00
VC1844	0.09	0.09	0.04	0.06
VC1845	0.09	0.08	0.08	0.07
VC1846	0.18	0.14	0.08	0.05
VC1847	0.04	0.03	0.06	0.06
VC1848	0.03	0.05	0.08	0.06
VC1849	0.05	0.05	0.10	0.09
VC1851	0.00	-0.02	0.00	-0.01
VC1852	0.10	0.09	0.11	0.09
VC1853	0.07	0.16	0.04	0.04
VC1854	0.15	0.13	0.08	0.09
VC1855	0.01	0.03	0.04	0.04

VC1856	0.11	0.12	0.06	0.04
VC1857	0.05	0.04	0.09	0.07
VC1858	0.05	0.05	0.05	0.06
VC1859	0.03	0.04	0.05	0.04
VC1860	0.12	0.12	0.05	0.06
VC1861	0.04	0.04	0.06	0.05
VC1862	0.09	0.10	0.05	0.06
VC1863	0.12	0.11	0.04	0.05
VC1864	0.05	0.06	0.05	0.06
VC1865	0.10	0.11	0.05	0.04
VC1866	0.04	0.04	0.04	0.05
VC1868	0.07	0.08	0.03	0.07
VC1869	0.03	0.05	0.05	0.06
VC1870	0.03	0.04	0.05	0.05
VC1871	0.04	0.08	0.03	0.03
VC1872	0.04	0.02	0.08	0.06
VC1873	0.06	0.05	0.06	0.06
VC1874	0.08	0.10	0.06	0.04
VC1875	0.07	0.08	0.07	0.07
VC1876	0.05	0.04	0.06	0.05
VC1877	0.01	0.01	0.01	0.01
VC1878	0.06	0.06	0.05	0.06
VC1879	0.04	0.03	0.04	0.06
VC1879	0.02	0.01	0.04	0.04
VC1880	0.09	0.10	0.04	0.05
VC1881	0.07	0.08	0.04	0.04
VC1882	0.02	0.02	0.00	0.01
VC1883	0.11	0.10	0.05	0.05
VC1884	0.12	0.11	0.05	0.04
VC1885	0.10	0.11	0.05	0.07
VC1886	0.01	0.01	0.07	0.06
VC1887	0.10	0.10	0.04	0.03
VC1888	0.02	0.01	0.04	0.05
VC1889	0.13	0.13	0.05	0.04
VC1890	0.13	0.13	0.07	0.07
VC1891	0.04	0.04	0.04	0.05
VC1891	0.07	0.07	0.04	0.03
VC1892	0.11	0.10	-0.02	0.02
VC1893	0.15	0.14	0.02	0.04
VC1894	0.07	0.05	0.03	0.03
VC1895	0.01	0.02	0.03	0.00
VC1896	0.08	0.08	0.03	0.05
VC1897	0.10	0.09	0.00	0.00

VC1898	0.04	0.05	0.06	0.06
VC1899	0.12	0.10	-0.01	0.03
VC1900	0.03	0.03	0.07	0.03
VC1901	0.09	0.08	0.03	0.03
VC1902	0.16	0.13	0.02	0.02
VC1903	0.06	0.05	0.06	0.03
VC1904	0.10	0.09	0.00	0.01
VC1905	0.13	0.12	0.00	0.01
VC1906	0.14	0.12	0.00	0.02
VC1907	0.05	0.05	0.00	0.01
VC1908	0.06	0.07	0.08	0.06
VC1909	0.04	0.07	0.04	0.04
VC1910	0.04	0.07	0.04	0.03
VC1911	0.05	0.04	0.03	0.03
VC1912	0.06	0.03	0.06	0.09
VC1913	0.06	0.05	0.04	0.04
VC1914	0.04	0.05	0.03	0.03
VC1915	0.06	0.09	0.05	0.05
VC1916	0.04	0.04	0.08	0.07
VC1917	0.04	0.03	0.06	0.04
VC1918	0.06	0.07	0.04	0.03
VC1919	0.09	0.09	0.07	0.07
VC1920	0.05	0.06	0.06	0.07
VC1921	0.01	0.02	0.02	0.04
VC1922	0.06	0.05	0.05	0.05
VC1923	0.06	0.06	0.08	0.08
VC1924	0.06	0.05	0.05	0.02
VC1925	0.07	0.07	0.07	0.05
VC1926	0.04	0.04	0.10	0.09
VC1927	0.05	0.06	0.04	0.04
VC1928	0.03	0.03	0.08	0.06
VC1929	0.09	0.07	0.07	0.09
VC1930	0.05	0.06	0.04	0.04
VC1931	0.06	0.05	0.04	0.05
VC1932	0.02	0.02	0.02	0.04
VC1933	0.11	0.12	0.06	0.05
VC1934	0.10	0.11	0.01	0.02
VC1935	0.01	0.01	0.05	0.06
VC1936	0.02	0.02	0.04	0.05
VC1937	0.05	0.05	0.06	0.06
VC1938	0.03	0.04	0.04	0.07
VC1938	0.02	0.02	0.05	0.04
VC1939	0.03	0.04	0.05	0.05

VC1940	0.05	0.05	0.05	0.04
VC1941	0.06	0.05	0.04	0.05
VC1942	0.00	0.02	0.10	0.11
VC1943	0.04	0.05	0.04	0.05
VC1943	0.06	0.06	0.01	0.03
VC1944	0.06	0.07	0.08	0.08
VC1945	0.06	0.06	0.03	0.04
VC1946	0.05	0.08	0.03	0.05
VC1947	0.03	0.03	0.06	0.06
VC1948	0.05	0.06	0.04	0.05
VC1949	0.08	0.04	0.05	0.05
VC1950	0.03	0.03	0.06	0.05
VC1951	0.05	0.05	0.03	0.03
VC1952	0.04	0.06	0.04	0.04
VC1953	0.08	0.04	0.09	0.14
VC1954	0.02	0.02	0.03	0.04
VC1955	0.02	0.03	0.09	0.04
VC1956	0.04	0.03	0.04	0.03
VC1957	0.08	0.06	0.07	0.07
VC1958	0.06	0.06	0.03	0.03
VC1959	0.03	0.02	0.02	0.06
VC1960	0.04	0.06	0.04	0.04
VC1961	0.05	0.05	0.05	0.07
VC1962	0.01	0.02	0.08	0.07
VC1963	0.07	0.09	0.05	0.04
VC1964	0.08	0.08	0.06	0.04
VC1965	0.06	0.07	0.06	0.07
VC1966	0.04	0.03	0.01	0.02
VC1967	0.01	0.02	0.06	0.06
VC1968	0.06	0.07	0.06	0.06
VC1969	0.05	0.06	0.04	0.05
VC1970	0.02	0.02	0.02	0.02
VC1971	0.03	0.06	0.06	0.05
VC1972	0.08	0.07	0.04	0.04
VC1973	0.06	0.06	0.06	0.06
VC1974	0.06	0.07	0.06	0.05
VC1975	0.07	0.06	0.03	0.02
VC1976	0.01	0.03	0.03	0.04
VC1977	0.02	0.01	0.05	0.05
VC1978	0.02	0.02	0.05	0.06
VC1979	0.02	0.03	0.11	0.11
VC1980	0.03	0.02	0.06	0.06
VC1981	0.03	0.02	0.04	0.06

VC1982	0.06	0.08	0.03	0.03
VC1983	0.08	0.09	0.04	0.04
VC1984	0.03	0.04	0.04	0.02
VC1985	0.09	0.10	0.04	0.05
VC1986	0.03	0.05	0.08	0.05
VC1987	0.02	0.02	0.05	0.05
VC1988	0.05	0.05	0.06	0.04
VC1989	0.03	0.02	0.09	0.07
VC1990	0.05	0.05	0.05	0.04
VC1991	0.03	0.05	0.04	0.04
VC1992	0.04	0.04	0.07	0.06
VC1993	0.03	0.03	0.04	0.06
VC1994	0.05	0.04	0.04	0.04
VC1995	0.03	0.02	0.11	0.11
VC1996	0.05	0.05	0.04	0.03
VC1997	0.06	0.07	0.03	0.03
VC1998	0.07	0.07	0.03	0.02
VC1999	0.06	0.07	0.05	0.03
VC2000	0.03	0.05	0.06	0.06
VC2001	0.05	0.05	0.07	0.05
VC2002	0.01	0.01	0.05	0.05
VC2003	0.07	0.07	0.03	0.04
VC2004	0.06	0.07	0.05	0.04
VC2005	0.09	0.10	0.03	0.04
VC2006	0.06	0.03	0.05	0.04
VC2007	0.06	0.07	0.09	0.08
VC2008	0.05	0.07	0.05	0.04
VC2009	0.07	0.06	0.05	0.05
VC2010	0.06	0.06	0.05	0.06
VC2011	0.01	0.01	0.01	0.01
VC2012	0.03	0.04	0.05	0.03
VC2013	0.03	0.03	0.06	0.04
VC2014	0.09	0.10	0.02	0.03
VC2015	0.06	0.06	0.04	0.03
VC2016	0.04	0.04	0.02	0.02
VC2017	0.06	0.07	0.04	0.04
VC2018	0.08	0.07	0.02	0.02
VC2019	0.06	0.06	0.07	0.04
VC2021	0.06	0.09	0.06	0.05
VC2022	0.06	0.07	0.07	0.05
VC2023	0.04	0.04	0.05	0.05
VC2024	0.00	0.03	0.02	0.03
VC2025	0.04	0.04	0.06	0.06

VC2026	0.04	0.04	0.05	0.05
VC2027	0.04	0.04	0.05	0.07
VC2028	0.05	0.06	0.05	0.06
VC2029	0.05	0.05	0.03	0.04
VC2030	0.01	0.02	0.00	0.01
VC2031	0.00	0.02	0.05	0.04
VC2032	0.03	0.03	0.04	0.04
VC2033	0.05	0.07	0.08	0.09
VC2034	0.07	0.07	0.06	0.07
VC2035	0.04	0.05	0.07	0.06
VC2036	0.03	0.03	0.06	0.03
VC2037	0.09	0.09	0.05	0.07
VC2039	0.06	0.07	0.05	0.05
VC2040	0.06	0.05	0.06	0.06
VC2041	0.00	0.01	0.04	0.07
VC2042	0.10	0.07	0.04	0.06
VC2043	0.04	0.06	0.04	0.05
VC2044	0.05	0.06	0.06	0.07
VC2045	0.02	0.05	0.08	0.06
VC2046	0.04	0.05	0.02	0.04
VC2047	0.06	0.05	0.07	0.09
VC2048	0.03	0.03	0.02	0.02
VC2049	-0.01	0.01	0.04	0.03
VC2051	0.06	0.07	0.04	0.06
VC2052	0.01	0.02	0.05	0.05
VC2053	0.06	0.06	0.03	0.03
VC2054	0.00	0.02	0.03	0.05
VC2055	0.00	0.03	0.03	0.03
VC2056	0.01	0.03	0.01	0.05
VC2057	0.04	0.04	0.04	0.04
VC2058	0.05	0.03	0.04	0.06
VC2059	0.03	0.03	0.07	0.06
VC2060	0.03	0.02	0.05	0.04
VC2061	0.01	0.01	0.05	0.04
VC2062	0.03	0.04	0.04	0.05
VC2063	0.04	0.06	0.06	0.05
VC2064	0.03	0.02	0.05	0.05
VC2065	0.10	0.09	0.04	0.04
VC2066	0.04	0.04	0.21	0.22
VC2067	0.05	0.03	0.04	0.05
VC2068	0.00	0.01	0.03	0.03
VC2069	0.06	0.06	0.05	0.06
VC2070	0.02	0.01	0.05	0.05

VC2071	0.05	0.05	0.04	0.05
VC2072	0.07	0.05	0.05	0.04
VC2073	0.03	0.04	0.03	0.07
VC2074	0.06	0.05	0.02	0.01
VC2075	0.07	0.08	0.03	0.03
VC2076	0.08	0.06	0.08	0.06
VC2077	0.03	0.02	0.05	0.06
VC2078	0.11	0.12	0.04	0.03
VC2079	0.08	0.08	0.02	0.05
VC2080	0.03	0.05	0.04	0.04
VC2081	0.05	0.05	0.05	0.04
VC2082	0.06	0.08	0.04	0.03
VC2083	0.02	0.01	0.09	0.07
VC2084	0.02	0.02	0.03	0.03
VC2085	0.02	0.03	0.01	0.01
VC2086	0.04	0.04	0.01	0.03
VC2087	0.01	0.01	0.06	0.05
VC2088	0.03	0.03	0.07	0.05
VC2089	0.01	0.02	0.15	0.13
VC2090	-0.01	0.02	0.03	0.03
VC2091	0.04	0.04	0.05	0.04
VC2092	0.06	0.06	0.07	0.07
VC2093	0.05	0.06	0.03	0.04
VC2094	0.04	0.04	0.11	0.10
VC2095	0.04	0.05	0.16	0.14
VC2096	0.02	0.03	0.24	0.20
VC2097	0.04	0.04	0.12	0.11
VC2098	0.02	0.03	0.02	0.00
VC2099	0.03	0.02	0.00	0.00
VC2100	0.03	0.04	0.11	0.12
VC2101	0.06	0.06	0.03	0.04
VC2102	0.05	0.07	0.02	0.04
VC2103	0.02	0.01	0.06	0.05
VC2105	0.07	0.08	0.04	0.04
VC2106	0.03	0.03	0.01	0.02
VC2107	0.03	0.02	0.05	0.05
VC2108	0.01	0.03	0.05	0.06
VC2109	0.02	0.02	0.07	0.10
VC2110	0.08	0.05	0.04	0.04
VC2111	0.05	0.06	0.03	0.02
VC2112	0.02	0.03	0.06	0.06
VC2112	0.01	0.01	0.05	0.05
VC2113	0.06	0.06	0.03	0.03

VC2114	0.05	0.06	0.01	0.02
VC2115	0.07	0.05	0.04	0.06
VC2116	0.03	0.03	0.06	0.08
VC2117	0.04	0.05	0.03	0.05
VC2118	0.02	0.03	0.05	0.06
VC2119	0.04	0.03	0.05	0.03
VC2120	0.02	0.03	0.04	0.03
VC2121	0.04	0.04	0.08	0.06
VC2122	0.06	0.07	0.02	0.04
VC2123	0.00	0.00	0.04	0.05
VC2124	0.03	0.05	0.03	0.04
VC2125	0.04	0.04	0.02	0.04
VC2126	0.01	0.01	0.05	0.05
VC2127	0.03	0.03	0.04	0.04
VC2128	0.02	0.02	0.04	0.04
VC2129	0.07	0.08	0.03	0.03
VC2130	-0.01	0.04	0.05	0.04
VC2131	0.06	0.08	0.06	0.08
VC2132	0.06	0.05	0.05	0.04
VC2133	0.01	0.02	0.04	0.04
VC2134	0.02	0.02	0.07	0.05
VC2134	-0.02	0.00	0.04	0.04
VC2135	0.05	0.04	0.10	0.10
VC2136	0.02	0.02	0.04	0.05
VC2137	0.02	0.04	0.15	0.15
VC2138	0.05	0.07	0.06	0.07
VC2139	0.05	0.05	0.08	0.08
VC2140	0.01	0.02	0.04	0.04
VC2141	0.03	0.04	0.06	0.06
VC2142	0.05	0.03	0.03	0.14
VC2143	0.04	0.02	0.07	0.06
VC2144	0.02	0.02	0.09	0.09
VC2145	0.05	0.04	0.11	0.09
VC2146	0.00	0.03	0.00	0.01
VC2147	0.03	0.03	0.03	0.02
VC2148	0.03	0.04	0.04	0.03
VC2149	0.03	0.03	0.06	0.04
VC2150	0.02	0.01	0.06	0.07
VC2151	0.04	0.05	0.04	0.04
VC2152	0.05	0.02	0.08	0.07
VC2153	0.02	0.03	0.04	0.07
VC2154	0.05	0.06	0.03	0.03
VC2155	0.02	0.02	0.05	0.06

VC2156	0.03	0.04	0.08	0.06
VC2157	0.04	0.04	0.03	0.03
VC2158	0.05	0.06	0.06	0.06
VC2159	0.00	0.01	0.10	0.09
VC2160	0.04	0.07	0.08	0.10
VC2161	-0.01	0.00	0.07	0.09
VC2162	0.05	0.05	0.06	0.05
VC2163	0.05	0.06	0.05	0.05
VC2164	0.09	0.09	0.05	0.07
VC2165	0.06	0.06	0.09	0.08
VC2166	0.02	0.03	0.07	0.07
VC2167	0.04	0.04	0.06	0.06
VC2168	0.07	0.07	0.03	0.02
VC2169	0.04	0.05	0.05	0.05
VC2170	0.05	0.05	0.04	0.04
VC2171	0.05	0.05	0.04	0.05
VC2172	0.01	0.01	0.06	0.05
VC2174	0.07	0.07	0.03	0.03
VC2175	0.05	0.05	0.04	0.02
VC2176	0.04	0.05	0.04	0.04
VC2177	-0.01	-0.01	0.06	0.04
VC2178	0.06	0.06	0.04	0.02
VC2179	0.02	0.03	0.02	0.02
VC2180	0.02	0.02	0.08	0.04
VC2181	0.03	0.03	0.03	0.06
VC2182	0.03	0.05	0.04	0.04
VC2183	0.01	0.02	0.01	0.02
VC2184	0.15	0.08	0.02	0.02
VC2185	0.04	0.04	0.03	0.00
VC2186	0.08	0.08	0.06	0.02
VC2187	0.04	0.04	0.05	0.06
VC2188	0.03	0.03	0.06	0.09
VC2189	0.01	0.05	0.05	0.05
VC2190	0.07	0.04	0.05	0.07
VC2191	0.04	0.03	0.05	0.05
VC2192	0.03	0.03	0.04	0.05
VC2193	0.03	0.04	0.03	0.03
VC2194	0.03	0.03	0.04	0.03
VC2195	0.01	0.11	0.03	0.04
VC2196	0.03	0.04	0.04	0.03
VC2197	0.03	0.03	0.07	0.06
VC2198	0.05	0.05	0.05	0.05
VC2199	0.03	0.03	0.03	0.04

VC2200	0.02	0.03	0.05	0.06
VC2201	0.02	0.04	0.03	0.04
VC2202	0.03	0.07	0.05	0.04
VC2203	0.02	0.03	0.02	0.03
VC2204	0.06	0.07	0.04	0.03
VC2205	0.04	0.07	0.07	0.06
VC2206	0.03	0.10	0.05	0.04
VC2207	0.08	0.09	0.02	0.03
VC2208	0.02	0.02	0.01	0.01
VC2209	0.00	0.01	0.03	0.05
VC2210	0.03	0.04	0.04	0.04
VC2211	0.02	0.02	0.04	0.13
VC2212	0.01	0.02	0.07	0.09
VC2213	0.02	0.03	0.01	0.01
VC2214	0.01	0.01	0.00	0.01
VC2215	0.01	0.02	0.04	0.04
VC2216	0.06	0.06	0.04	0.03
VC2217	0.01	0.01	0.02	0.02
VC2218	0.09	0.08	0.02	0.03
VC2219	0.08	0.06	0.01	0.01
VC2220	0.02	0.03	0.00	0.01
VC2221	0.09	0.07	0.05	0.05
VC2222	0.07	0.07	0.07	0.08
VC2223	0.04	0.05	0.03	0.04
VC2224	0.02	0.02	0.01	0.02
VC2225	0.09	0.08	0.03	0.04
VC2226	0.05	0.06	0.06	0.04
VC2227	0.06	0.05	0.07	0.07
VC2228	0.09	0.08	0.02	0.02
VC2229	0.05	0.07	0.04	0.03
VC2230	0.06	0.06	0.06	0.07
VC2231	0.04	0.05	0.05	0.05
VC2232	0.05	0.08	0.06	0.06
VC2233	0.03	0.02	0.01	0.01
VC2234	0.05	0.06	0.08	0.05
VC2235	0.05	0.07	0.04	0.05
VC2236	0.14	0.13	0.08	0.08
VC2237	0.03	0.03	0.05	0.01
VC2238	0.08	0.08	0.09	0.07
VC2239	0.08	0.09	0.08	0.09
VC2240	0.05	0.06	0.05	0.04
VC2241	0.05	0.06	0.05	0.06
VC2242	0.03	0.03	0.09	0.08

VC2243	0.04	0.06	0.05	0.05
VC2244	0.05	0.06	0.05	0.06
VC2245	0.07	0.07	0.08	0.05
VC2246	0.05	0.06	0.03	0.04
VC2247	0.05	0.05	0.05	0.04
VC2248	0.07	0.07	0.09	0.10
VC2249	0.06	0.08	0.06	0.05
VC2250	0.04	0.04	0.04	0.04
VC2252	0.01	0.02	0.04	0.05
VC2253	0.01	0.02	0.05	0.04
VC2254	0.02	0.03	0.05	0.05
VC2255	0.01	0.02	0.01	0.01
VC2256	0.05	0.06	0.06	0.07
VC2258	0.02	0.02	0.05	0.06
VC2258	0.04	0.05	0.09	0.06
VC2259	0.09	0.09	0.05	0.04
VC2260	0.03	0.03	0.01	0.02
VC2261	0.06	0.07	0.07	0.07
VC2262	0.06	0.06	0.05	0.04
VC2263	0.04	0.09	0.05	0.03
VC2263	0.05	0.06	0.04	0.05
VC2264	0.05	0.05	0.09	0.05
VC2265	0.11	0.13	0.06	0.07
VC2266	0.03	0.05	0.05	0.06
VC2267	0.11	0.09	0.05	0.09
VC2268	0.08	0.07	0.11	0.07
VC2269	0.06	0.04	0.12	0.08
VC2270	0.05	0.05	0.08	0.07
VC2271	0.01	0.01	0.01	0.01
VC2272	0.08	0.06	0.05	0.05
VC2273	0.08	0.06	0.04	0.04
VC2274	0.04	0.04	0.07	0.06
VC2275	0.07	0.07	0.09	0.09
VC2276	0.10	0.11	0.06	0.08
VC2277	0.06	0.08	0.09	0.09
VC2278	0.03	0.05	0.01	0.03
VC2279	0.05	0.05	0.06	0.05
VC2280	0.08	0.07	0.13	0.12
VC2281	0.05	0.05	0.06	0.06
VC2282	0.00	0.02	0.00	0.04
VC2282	0.01	0.05	0.05	0.06
VC2283	0.12	0.15	0.07	0.07
VC2284	0.05	0.04	0.05	0.06

VC2285	-0.02	0.00	-0.01	-0.01
VC2286	0.03	0.05	0.07	0.08
VC2287	0.06	0.03	0.04	0.06
VC2288	0.06	0.06	0.00	0.03
VC2289	0.03	0.03	0.04	0.03
VC2290	0.09	0.07	0.06	0.06
VC2291	0.04	0.05	0.02	0.03
VC2292	0.04	0.05	0.06	0.06
VC2293	0.05	0.05	0.08	0.08
VC2294	0.03	0.03	0.05	0.05
VC2295	0.08	0.07	0.05	0.06
VC2296	0.05	0.06	0.09	0.09
VC2297	0.06	0.06	0.05	0.05
VC2298	0.09	0.07	0.08	0.10
VC2299	0.04	0.05	0.03	0.04
VC2300	0.05	0.06	0.06	0.05
VC2301	0.07	0.08	0.00	0.02
VC2302	0.05	0.06	0.09	0.06
VC2303	0.05	0.05	0.04	0.04
VC2304	0.03	0.03	0.05	0.04
VC2305	0.08	0.07	0.04	0.05
VC2306	0.04	0.05	0.04	0.04
VC2307	0.09	0.08	0.09	0.12
VC2308	0.04	0.04	0.04	0.06
VC2309	0.06	0.06	0.09	0.09
VC2310	0.04	0.04	0.05	0.06
VC2311	0.09	0.09	0.11	0.10
VC2312	0.06	0.06	0.08	0.10
VC2313	0.06	0.07	0.09	0.07
VC2314	0.05	0.04	0.06	0.05
VC2315	0.07	0.09	0.02	0.03
VC2316	0.04	0.04	0.08	0.07
VC2317	0.07	0.08	0.03	0.03
VC2317	0.05	0.05	0.02	0.03
VC2318	0.07	0.08	0.11	0.11
VC2319	-0.01	-0.02	0.04	0.04
VC2320	0.03	0.07	0.12	0.10
VC2321	0.05	0.05	0.05	0.05
VC2322	0.01	0.01	0.04	0.06
VC2323	0.09	0.07	0.05	0.05
VC2324	0.07	0.06	0.07	0.06
VC2325	0.05	0.04	0.05	0.07
VC2326	0.05	0.04	0.08	0.08

VC2327	0.06	0.06	0.06	0.08
VC2328	0.03	0.07	0.07	0.07
VC2329	0.03	0.03	0.08	0.03
VC2330	0.04	0.04	0.08	0.08
VC2331	0.06	0.05	0.03	0.02
VC2331	0.05	0.05	0.05	0.05
VC2332	0.04	0.04	0.08	0.07
VC2333	0.10	0.11	0.05	0.04
VC2334	0.06	0.06	0.04	0.04
VC2335	0.05	0.06	0.10	0.08
VC2336	0.04	0.04	0.06	0.10
VC2337	0.05	0.04	0.08	0.07
VC2338	0.01	0.01	0.05	0.05
VC2339	0.12	0.10	0.07	0.07
VC2340	0.09	0.07	0.08	0.07
VC2341	0.06	0.05	0.03	0.04
VC2342	0.03	0.05	0.04	0.05
VC2343	0.03	0.03	0.12	0.10
VC2344	0.62	0.65	0.58	0.55
VC2345	0.10	0.11	0.05	0.05
VC2346	0.09	0.09	0.08	0.09
VC2347	0.06	0.03	0.05	0.04
VC2348	0.06	0.05	0.08	0.07
VC2349	0.17	0.15	0.07	0.06
VC2350	0.11	0.12	0.08	0.06
VC2351	0.04	0.05	0.05	0.06
VC2352	0.05	0.04	0.07	0.07
VC2353	0.11	0.11	0.05	0.05
VC2354	0.05	0.06	0.12	0.12
VC2355	0.12	0.13	0.05	0.05
VC2356	0.02	0.05	0.01	0.01
VC2356	-0.01	0.03	0.05	0.04
VC2357	0.06	0.07	0.07	0.06
VC2358	0.01	0.01	0.00	0.00
VC2359	0.07	0.07	0.08	0.09
VC2360	0.05	0.07	0.04	0.05
VC2361	0.10	0.10	0.03	0.04
VC2362	0.08	0.09	0.06	0.05
VC2363	0.06	0.05	0.03	0.03
VC2364	0.01	0.01	0.04	0.04
VC2364.1	0.03	0.04	0.04	0.04
VC2364.1	0.06	0.05	0.04	0.04
VC2365	0.14	0.17	0.05	0.07

VC2366	0.13	0.13	0.03	0.03
VC2367	0.08	0.08	0.07	0.08
VC2368	0.12	0.10	0.08	0.08
VC2369	0.13	0.14	0.04	0.05
VC2370	0.23	0.27	0.23	0.24
VC2371	0.15	0.14	0.01	0.03
VC2372	0.11	0.11	0.03	0.02
VC2373	0.02	0.02	0.00	0.04
VC2373	0.01	0.01	0.05	0.06
VC2374	0.12	0.08	0.05	0.04
VC2376	0.03	0.02	0.02	0.02
VC2376	0.02	0.02	0.03	0.05
VC2377	0.04	0.04	0.08	0.07
VC2378	0.12	0.13	0.05	0.02
VC2379	0.10	0.10	0.01	0.00
VC2380	0.08	0.05	0.02	0.00
VC2381	0.04	0.04	0.02	0.01
VC2382	0.07	0.06	0.01	0.01
VC2383	0.06	0.06	0.07	0.06
VC2384	0.09	0.08	0.02	0.01
VC2385	0.04	0.04	0.08	0.06
VC2386	0.11	0.10	0.05	0.05
VC2387	0.10	0.12	0.01	0.01
VC2388	0.11	0.09	0.02	0.01
VC2389	0.03	0.03	0.01	0.03
VC2390	0.02	0.03	0.02	0.03
VC2391	0.10	0.09	0.01	0.00
VC2392	0.11	0.12	0.05	0.02
VC2393	0.05	0.06	0.04	0.05
VC2394	0.03	0.04	0.08	0.06
VC2395	0.20	0.18	-0.02	0.00
VC2396	0.09	0.08	0.00	0.02
VC2397	0.03	0.03	0.03	0.01
VC2398	0.03	0.03	0.03	0.04
VC2399	0.12	0.11	0.02	0.02
VC2400	0.00	0.01	0.01	0.02
VC2401	0.02	0.03	0.06	0.05
VC2402	0.04	0.03	0.04	0.03
VC2403	0.16	0.15	0.04	0.04
VC2404	0.02	0.04	0.05	0.04
VC2405	0.10	0.09	0.06	0.07
VC2406	0.08	0.08	0.05	0.05
VC2408	0.06	0.08	0.05	0.05

VC2409	0.10	0.09	-0.01	0.00
VC2410	0.05	0.06	0.05	0.04
VC2410	0.06	0.08	0.05	0.04
VC2411	0.07	0.06	0.04	0.04
VC2411	0.07	0.09	0.05	0.05
VC2412	0.05	0.04	0.02	0.03
VC2414	0.00	0.01	0.04	0.04
VC2415	0.11	0.10	0.02	0.02
VC2416	0.08	0.08	0.05	0.05
VC2417	0.05	0.04	0.02	0.03
VC2419	0.08	0.09	0.05	0.04
VC2420	0.08	0.05	0.05	0.04
VC2421	0.08	0.08	0.08	0.07
VC2422	0.06	0.05	0.08	0.07
VC2423	0.06	0.04	0.08	0.06
VC2424	0.05	0.04	0.01	0.02
VC2425	0.02	0.02	0.05	0.06
VC2426	0.03	0.03	0.03	0.03
VC2427	0.06	0.05	0.05	0.03
VC2428	0.05	0.06	0.10	0.10
VC2429	0.04	0.05	0.06	0.06
VC2430	0.16	0.20	0.04	0.05
VC2431	0.06	0.07	0.06	0.04
VC2432	0.10	0.09	0.13	0.10
VC2433	0.08	0.08	0.10	0.13
VC2434	0.10	0.07	0.09	0.05
VC2435	0.08	0.07	0.08	0.08
VC2436	0.08	0.05	0.08	0.05
VC2437	0.01	0.02	0.04	0.05
VC2438	0.04	0.02	0.03	0.03
VC2439	0.03	0.06	0.06	0.07
VC2440	0.02	0.02	0.05	0.06
VC2440	0.02	0.03	0.04	0.03
VC2441	0.07	0.05	0.06	0.06
VC2442	0.08	0.07	0.08	0.05
VC2443	0.07	0.07	0.08	0.06
VC2444	0.01	0.02	0.03	0.02
VC2445	0.00	0.02	0.02	0.04
VC2445	0.01	0.03	0.05	0.04
VC2446	0.06	0.06	0.08	0.07
VC2447	0.08	0.06	0.07	0.07
VC2448	0.03	0.03	0.00	0.01
VC2449	0.06	0.06	0.02	0.02

VC2450	0.04	0.04	0.03	0.05
VC2451	0.03	0.04	0.05	0.06
VC2452	0.10	0.11	0.05	0.05
VC2453	0.05	0.06	0.03	0.03
VC2454	-0.01	0.00	-0.01	0.00
VC2455	0.11	0.10	0.05	0.06
VC2456	0.06	0.07	0.06	0.06
VC2457	0.03	0.04	0.06	0.05
VC2458	0.05	0.06	0.12	0.09
VC2459	0.01	0.01	0.10	0.08
VC2460	0.07	0.06	0.05	0.04
VC2461	0.08	0.08	0.10	0.08
VC2462	0.05	0.06	0.05	0.05
VC2463	0.04	0.03	0.06	0.04
VC2464	0.01	0.01	0.10	0.09
VC2465	0.00	0.00	0.02	0.01
VC2466	0.02	0.02	0.07	0.05
VC2467	0.06	0.06	0.05	0.05
VC2468	0.04	0.03	0.07	0.04
VC2469	0.04	0.05	0.07	0.05
VC2470	0.03	0.03	0.03	0.04
VC2471	0.07	0.06	0.03	0.03
VC2472	0.02	0.04	0.05	0.07
VC2473	0.07	0.08	0.03	0.03
VC2474	0.03	0.04	0.06	0.05
VC2475	0.05	0.04	0.03	0.05
VC2476	0.06	0.05	0.02	0.04
VC2478	0.04	0.06	0.05	0.07
VC2479	0.05	0.04	0.06	0.04
VC2480	0.05	0.05	0.09	0.11
VC2481	0.05	0.03	0.03	0.05
VC2482	0.04	0.03	0.06	0.07
VC2483	0.06	0.08	0.06	0.05
VC2484	0.06	0.07	0.05	0.05
VC2485	0.05	0.04	0.05	0.04
VC2486	0.04	0.04	0.08	0.08
VC2487	0.05	0.06	0.03	0.03
VC2488	0.09	0.08	0.03	0.09
VC2489	0.03	0.04	0.03	0.04
VC2490	0.04	0.04	0.05	0.07
VC2491	0.04	0.03	0.06	0.04
VC2492	0.09	0.07	0.06	0.07
VC2493	0.16	0.04	0.05	0.05

VC2494	0.03	0.03	0.03	0.03
VC2495	0.05	0.05	0.07	0.08
VC2496	0.04	0.05	0.07	0.07
VC2497	0.03	0.03	0.08	0.07
VC2498	0.01	0.02	0.03	0.03
VC2499	0.03	0.02	0.06	0.03
VC2500	0.02	0.03	0.01	0.00
VC2501	0.03	0.04	0.09	0.09
VC2502	0.04	0.05	0.07	0.07
VC2503	0.05	0.06	0.05	0.05
VC2504	0.05	0.05	0.09	0.08
VC2505	0.07	0.06	0.05	0.03
VC2506	0.02	0.02	0.06	0.07
VC2507	0.02	0.01	0.04	0.04
VC2508	0.07	0.07	0.07	0.06
VC2509	0.06	0.06	0.04	0.03
VC2511	0.08	0.09	0.06	0.06
VC2512	0.06	0.07	0.07	0.06
VC2513	0.12	0.11	0.06	0.07
VC2514	0.09	0.09	0.12	0.12
VC2515	0.07	0.08	0.05	0.05
VC2516	0.05	0.06	0.04	0.03
VC2517	0.05	0.05	0.09	0.08
VC2518	0.06	0.05	0.03	0.03
VC2519	0.03	0.01	0.04	0.05
VC2520	0.07	0.07	0.05	0.06
VC2521	0.03	0.03	0.04	0.05
VC2522	0.05	0.05	0.06	0.07
VC2523	0.07	0.05	0.02	0.03
VC2524	0.04	0.04	0.03	0.03
VC2525	0.04	0.08	0.03	0.05
VC2527	0.02	0.00	0.05	0.04
VC2528	0.06	0.06	0.04	0.03
VC2529	0.14	0.11	0.21	0.12
VC2530	0.09	0.09	0.06	0.03
VC2531	0.07	0.04	0.05	0.06
VC2532	0.06	0.09	0.06	0.06
VC2534	0.04	0.02	0.05	0.05
VC2535	0.03	0.03	0.05	0.05
VC2536	0.07	0.08	0.04	0.03
VC2537	0.06	0.04	0.05	0.12
VC2538	0.04	0.05	0.09	0.05
VC2539	0.07	0.05	0.08	0.09

VC2540	0.05	0.09	0.05	0.06
VC2541	0.05	0.05	0.05	0.06
VC2542	0.05	0.05	0.05	0.05
VC2543	0.05	0.06	0.07	0.06
VC2544	0.07	0.05	0.08	0.07
VC2545	0.07	0.05	0.08	0.09
VC2546	0.07	0.07	0.05	0.03
VC2547	0.05	0.05	0.04	0.04
VC2547	0.01	0.04	0.04	0.03
VC2548	0.03	0.02	0.05	0.06
VC2549	0.04	0.03	0.08	0.09
VC2551	0.07	0.06	0.04	0.03
VC2552	0.08	0.09	0.05	0.07
VC2553	0.09	0.10	0.07	0.05
VC2554	0.03	0.03	0.06	0.07
VC2555	0.02	0.02	0.03	0.04
VC2557	0.06	0.05	0.03	0.03
VC2558	0.04	0.05	0.05	0.04
VC2559	0.04	0.04	0.02	0.02
VC2560	0.02	0.03	0.04	0.04
VC2561	0.04	0.04	0.03	0.04
VC2562	0.02	0.03	0.03	0.04
VC2563	0.03	0.04	0.06	0.06
VC2564	0.05	0.05	0.04	0.04
VC2565	0.05	0.06	0.06	0.05
VC2566	0.02	0.02	0.05	0.03
VC2567	0.03	0.05	0.04	0.04
VC2568	0.06	0.05	0.08	0.09
VC2569	0.06	0.06	0.05	0.05
VC2570	0.02	0.02	0.07	0.13
VC2571	0.04	0.05	0.06	0.04
VC2572	0.03	0.04	0.02	0.02
VC2573	0.02	0.03	0.02	0.02
VC2574	0.07	0.06	0.07	0.06
VC2575	0.06	0.07	0.06	0.08
VC2576	0.04	0.05	0.04	0.04
VC2578	0.08	0.08	0.06	0.07
VC2579	0.03	0.03	0.08	0.05
VC2580	0.07	0.07	0.05	0.05
VC2581	0.02	0.03	0.07	0.07
VC2582	0.03	0.03	0.04	0.04
VC2583	0.05	0.06	0.09	0.09
VC2584	0.03	0.04	0.03	0.03

VC2585	0.02	0.03	0.01	0.02
VC2586	0.02	0.02	0.07	0.05
VC2587	0.08	0.07	0.07	0.07
VC2588	0.09	0.06	0.04	0.04
VC2589	0.11	0.12	0.05	0.07
VC2590	0.05	0.06	0.03	0.04
VC2591	0.03	0.05	0.04	0.06
VC2592	0.04	0.04	0.05	0.06
VC2593	0.00	0.02	0.03	0.04
VC2594	0.03	0.02	0.04	0.07
VC2595	0.04	0.05	0.02	0.04
VC2596	0.02	0.02	0.06	0.01
VC2597	0.04	0.03	0.01	0.01
VC2598	0.00	0.02	0.04	0.08
VC2599	0.05	0.05	0.08	0.07
VC2600	0.06	0.05	0.02	0.03
VC2601	0.05	0.06	0.03	0.05
VC2602	0.04	0.06	0.04	0.04
VC2603	0.01	0.01	0.06	0.05
VC2604	-0.01	0.01	0.04	0.09
VC2605	0.08	0.09	0.08	0.07
VC2606	0.04	0.09	0.08	0.08
VC2607	0.06	0.07	0.05	0.05
VC2608	0.05	0.03	0.03	0.02
VC2609	0.07	0.05	0.04	0.07
VC2610	0.05	0.05	0.05	0.03
VC2611	0.05	0.05	0.05	0.05
VC2612	0.07	0.07	0.07	0.06
VC2613	0.05	0.06	0.05	0.04
VC2614	0.07	0.06	0.05	0.04
VC2615	0.08	0.06	0.07	0.07
VC2616	0.07	0.06	0.07	0.06
VC2617	0.04	0.04	0.06	0.05
VC2618	0.03	0.03	0.09	0.10
VC2619	0.05	0.05	0.05	0.05
VC2620	0.02	0.03	0.04	0.04
VC2621	0.01	0.02	0.06	0.06
VC2622	0.04	0.05	0.03	0.04
VC2623	0.05	0.05	0.06	0.02
VC2624	0.06	0.05	0.05	0.04
VC2625	0.04	0.03	0.09	0.08
VC2626	0.09	0.08	0.07	0.05
VC2627	0.04	0.03	0.02	0.02

VC2628	0.06	0.05	0.05	0.06
VC2629	0.03	0.03	0.06	0.05
VC2631	0.08	0.06	0.10	0.09
VC2632	0.02	0.01	0.05	0.04
VC2633	0.03	0.03	0.03	0.04
VC2634	0.03	0.05	0.05	0.05
VC2635	0.04	0.05	0.04	0.04
VC2636	0.01	0.00	0.01	0.04
VC2637	0.01	0.04	0.04	0.07
VC2638	0.05	0.05	0.12	0.13
VC2639	0.11	0.08	0.04	0.02
VC2640	0.07	0.07	0.04	0.04
VC2641	0.03	0.04	0.04	0.06
VC2642	0.05	0.04	0.16	0.17
VC2643	0.01	0.01	0.04	0.03
VC2644	0.06	0.06	0.05	0.05
VC2645	0.04	0.04	0.06	0.05
VC2646	0.04	0.05	0.05	0.05
VC2647	0.03	0.05	0.06	0.03
VC2648	0.10	0.07	0.01	0.04
VC2649	0.10	0.08	0.02	0.02
VC2650	0.05	0.05	0.10	0.10
VC2651	0.03	0.04	0.07	0.05
VC2652	0.08	0.06	0.06	0.04
VC2653	0.04	0.03	0.11	0.14
VC2654	0.00	0.00	0.08	0.05
VC2655	0.03	0.04	0.05	0.03
VC2656	0.02	0.00	0.06	0.07
VC2657	0.06	0.05	0.06	0.07
VC2658	0.03	0.02	0.04	0.07
VC2659	0.05	0.05	0.05	0.04
VC2660	0.05	0.05	0.09	0.05
VC2661	0.01	0.01	0.05	0.06
VC2662	0.01	0.01	0.05	0.07
VC2663	0.08	0.05	0.04	0.05
VC2664	0.06	0.06	0.06	0.06
VC2665	0.07	0.08	0.05	0.07
VC2666	0.05	0.05	0.07	0.03
VC2667	0.03	0.04	0.04	0.03
VC2668	0.03	0.04	0.05	0.04
VC2669	0.05	0.04	0.07	0.06
VC2670	0.04	0.04	0.01	0.01
VC2671	0.04	0.05	0.06	0.05

VC2672	0.05	0.05	0.08	0.07
VC2673	0.00	0.01	0.05	0.05
VC2674	0.05	0.06	0.06	0.05
VC2675	0.02	0.02	0.07	0.08
VC2676	0.02	0.02	0.04	0.04
VC2677	0.06	0.06	0.09	0.07
VC2678	0.10	0.11	0.05	0.04
VC2679	0.03	0.03	0.05	0.05
VC2680	0.05	0.05	0.07	0.03
VC2680	0.06	0.07	0.03	0.04
VC2681	0.04	0.05	0.05	0.04
VC2682	0.04	0.04	0.07	0.09
VC2683	0.04	0.05	0.04	0.05
VC2684	0.03	0.06	0.03	0.06
VC2685	0.06	0.07	0.06	0.06
VC2686	0.04	0.04	0.09	0.07
VC2687	0.06	0.08	0.05	0.05
VC2688	0.04	0.04	0.06	0.03
VC2689	0.06	0.07	0.03	0.06
VC2690	0.10	0.11	0.05	0.05
VC2691	0.05	0.04	0.07	0.07
VC2692	0.05	0.07	0.07	0.08
VC2693	0.00	0.03	0.06	0.06
VC2694	0.04	0.05	0.06	0.05
VC2695	0.05	0.06	0.09	0.08
VC2696	0.05	0.03	0.03	0.03
VC2697	0.00	0.01	0.01	0.00
VC2698	0.05	0.05	0.04	0.06
VC2699	0.04	0.03	0.03	0.04
VC2700	0.05	0.05	0.04	0.04
VC2701	0.04	0.05	0.04	0.05
VC2702	0.05	0.04	0.11	0.08
VC2703	0.02	0.01	0.04	0.05
VC2704	0.06	0.06	0.02	0.04
VC2705	-0.01	-0.01	0.05	0.04
VC2706	0.01	0.00	0.07	0.05
VC2708	0.02	0.01	0.04	0.06
VC2709	0.06	0.06	0.06	0.05
VC2710	0.05	0.05	0.06	0.07
VC2711	0.10	0.06	0.06	0.07
VC2712	0.01	0.03	0.04	0.05
VC2713	0.04	0.04	0.08	0.05
VC2714	0.02	0.05	0.06	0.04

VC2715	0.05	0.03	0.05	0.05
VC2716	0.09	0.07	0.07	0.06
VC2717	0.01	0.02	0.06	0.06
VC2718	0.08	0.08	0.02	0.01
VC2719	0.01	0.01	0.10	0.10
VC2720	0.05	0.06	0.06	0.05
VC2721	0.03	0.03	0.04	0.05
VC2722	0.02	0.06	0.06	0.05
VC2723	0.02	0.05	0.05	0.06
VC2724	0.02	0.03	0.00	0.00
VC2725	0.08	0.09	0.04	0.06
VC2726	0.11	0.08	0.04	0.05
VC2727	0.03	0.03	0.05	0.05
VC2728	0.05	0.06	0.06	0.07
VC2729	0.04	0.05	0.04	0.04
VC2730	0.04	0.04	0.06	0.03
VC2731	0.04	0.05	0.04	0.04
VC2732	0.05	0.06	0.04	0.03
VC2733	0.03	0.03	0.03	0.02
VC2734	0.02	0.03	0.04	0.04
VC2735	0.04	0.04	0.07	0.07
VC2736	0.03	0.04	0.05	0.04
VC2737	0.08	0.08	0.01	0.05
VC2738	0.05	0.06	0.04	0.05
VC2739	0.03	0.03	0.03	0.07
VC2740	0.08	0.09	0.06	0.08
VC2741	0.05	0.05	0.03	0.03
VC2742	0.03	0.04	0.05	0.05
VC2743	0.04	0.09	0.06	0.07
VC2744	0.07	0.08	0.06	0.04
VC2745	0.05	0.04	0.04	0.04
VC2746	0.06	0.06	0.04	0.04
VC2747	0.05	0.05	0.04	0.04
VC2748	0.00	0.03	0.05	0.08
VC2749	0.06	0.07	0.07	0.05
VC2750	-0.01	0.00	0.03	0.03
VC2751	0.04	0.03	0.04	0.05
VC2752	0.06	0.05	0.06	0.06
VC2754	0.06	0.07	0.06	-0.04
VC2755	0.05	0.05	0.07	0.07
VC2756	0.07	0.06	0.00	0.01
VC2757	0.08	0.06	0.01	0.01
VC2758	0.08	0.07	0.08	0.07

VC2759	0.02	0.02	0.00	0.02
VC2760	0.03	0.03	0.07	0.06
VC2761	0.02	0.02	0.04	0.05
VC2762	0.10	0.09	0.06	0.08
VC2763	0.03	0.03	0.08	0.06
VC2764	0.03	0.01	0.00	-0.01
VC2765	0.02	0.03	0.06	0.05
VC2767	0.04	0.04	0.03	0.02
VC2768	0.04	0.03	0.00	0.01
VC2769	0.09	0.03	0.07	0.07
VC2770	0.08	0.04	0.04	0.03
VC2771	0.05	0.05	0.05	0.05
VC2772	0.05	0.07	0.10	0.10
VC2773	0.02	0.03	0.04	0.08
VC2774	0.03	0.03	0.05	0.06
VC2775	0.04	0.03	0.06	0.07
VCA0001	0.10	0.05	0.05	0.04
VCA0002	0.02	0.02	0.04	0.04
VCA0003	0.05	0.03	0.07	0.08
VCA0004	0.03	0.04	0.06	0.07
VCA0005	0.02	0.03	0.07	0.07
VCA0006	0.03	0.03	0.02	0.02
VCA0007	0.03	0.04	0.11	0.08
VCA0008	0.04	0.04	0.02	0.02
VCA0009	0.03	0.03	0.03	0.04
VCA0010	0.04	0.03	0.03	0.02
VCA0011	0.02	0.04	0.05	0.05
VCA0012	0.06	0.06	0.05	0.05
VCA0013	0.01	0.04	0.06	0.06
VCA0014	0.04	0.04	0.06	0.06
VCA0016	0.11	0.05	0.04	0.05
VCA0017	0.05	0.06	0.06	0.07
VCA0018	0.03	0.04	0.05	0.04
VCA0019	0.00	0.02	0.06	0.08
VCA0020	0.05	0.05	0.05	0.04
VCA0021	0.03	0.02	0.04	0.03
VCA0022	0.03	0.01	0.03	0.04
VCA0023	0.03	0.03	0.13	0.11
VCA0024	0.03	0.01	0.03	0.03
VCA0025	0.02	0.02	0.02	0.03
VCA0026	0.02	0.03	0.07	0.06
VCA0027	0.07	0.05	0.07	0.06
VCA0028	0.04	0.09	0.05	0.05

VCA0029	0.04	0.06	0.08	0.09
VCA0030	0.06	0.07	0.02	0.07
VCA0031	0.08	0.09	0.03	0.03
VCA0032	0.05	0.07	0.07	0.06
VCA0033	0.04	0.06	0.05	0.04
VCA0034	0.03	0.05	0.04	0.06
VCA0035	0.06	0.09	0.06	0.06
VCA0036	0.15	0.15	0.09	0.05
VCA0037	0.09	0.12	0.06	0.07
VCA0038	0.04	0.05	0.05	0.04
VCA0039	0.03	0.08	0.04	0.06
VCA0040	0.06	0.08	0.06	0.06
VCA0041	0.06	0.06	0.05	0.05
VCA0042	0.58	0.62	0.49	0.55
VCA0043	0.05	0.06	0.08	0.08
VCA0044	0.03	0.07	0.04	0.05
VCA0046	0.03	0.04	0.06	0.06
VCA0047	0.08	0.08	0.07	0.05
VCA0048	0.06	0.12	0.04	0.04
VCA0049	0.04	0.04	0.02	0.00
VCA0050	0.11	0.08	0.04	0.04
VCA0051	0.02	0.01	0.06	0.08
VCA0052	0.04	0.04	0.04	0.05
VCA0053	0.04	0.07	0.06	0.07
VCA0054	0.03	0.05	0.08	0.07
VCA0055	0.05	0.06	0.08	0.06
VCA0056	0.05	0.08	0.06	0.05
VCA0057	0.10	0.10	0.11	0.11
VCA0058	0.07	0.08	0.07	0.05
VCA0060	0.06	0.09	0.04	0.04
VCA0061	0.04	0.04	0.06	0.06
VCA0062	0.04	0.05	0.04	0.03
VCA0062	0.04	0.04	0.06	0.06
VCA0063	0.06	0.09	0.03	0.03
VCA0064	0.03	0.03	0.05	0.06
VCA0065	0.06	0.05	0.05	0.04
VCA0066	0.05	0.04	0.04	0.04
VCA0067	0.04	0.05	0.07	0.05
VCA0068	0.03	0.03	0.06	0.05
VCA0069	0.06	0.07	0.02	0.03
VCA0069	0.05	0.06	0.05	0.05
VCA0070	0.04	0.05	0.09	0.07
VCA0071	0.15	0.13	0.19	0.22

VCA0072	0.07	0.07	0.11	0.09
VCA0073	0.06	0.05	0.09	0.04
VCA0074	0.00	0.02	0.06	0.01
VCA0075	0.02	0.02	0.05	0.05
VCA0076	0.03	0.03	0.02	0.05
VCA0077	0.04	0.03	0.03	0.03
VCA0078	0.04	0.05	0.02	0.01
VCA0079	0.06	0.06	0.07	-0.04
VCA0080	0.13	0.14	-0.01	-0.01
VCA0081	0.06	0.06	0.04	0.04
VCA0082	0.09	0.08	0.09	0.08
VCA0083	0.06	0.05	0.09	0.06
VCA0084	0.07	0.06	0.11	0.08
VCA0085	0.07	0.06	0.11	0.05
VCA0086	0.06	0.06	0.12	0.10
VCA0087	0.07	0.06	0.05	0.04
VCA0089	0.05	0.05	0.03	0.04
VCA0090	0.03	0.03	0.04	0.04
VCA0091	0.07	0.07	0.12	0.09
VCA0092	0.07	0.08	0.09	0.09
VCA0093	0.06	0.10	0.07	0.06
VCA0093	0.06	0.07	0.04	0.05
VCA0094	0.07	0.07	0.10	0.12
VCA0095	0.06	0.06	0.08	0.06
VCA0096	0.02	0.01	0.09	0.09
VCA0097	0.06	0.10	0.06	0.05
VCA0098	0.04	0.04	0.03	0.03
VCA0099	0.07	0.06	0.12	0.09
VCA0100	0.05	0.06	0.09	0.08
VCA0101	0.07	0.05	0.04	0.08
VCA0102	0.05	0.06	0.06	0.05
VCA0103	0.04	0.03	0.02	0.05
VCA0104	0.07	0.06	0.04	0.08
VCA0105	0.05	0.07	0.10	0.08
VCA0106	0.03	0.03	0.05	0.04
VCA0107	0.06	0.06	0.12	0.08
VCA0108	0.02	0.06	0.05	0.04
VCA0109	0.07	0.06	0.09	0.09
VCA0110	0.04	0.03	0.03	0.05
VCA0111	0.03	0.05	0.09	0.06
VCA0112	0.04	0.05	0.06	0.06
VCA0113	0.06	0.05	0.05	0.10
VCA0114	0.06	0.06	0.16	0.09

VCA0115	0.03	0.04	0.01	0.02
VCA0116	0.13	0.09	0.05	0.06
VCA0117	0.08	0.08	0.04	0.08
VCA0118	0.04	0.05	0.09	0.06
VCA0119	0.03	0.04	0.07	0.07
VCA0120	0.03	0.03	0.06	0.06
VCA0121	0.05	0.06	0.12	0.06
VCA0122	0.06	0.05	0.12	0.10
VCA0123	0.05	0.04	0.04	0.04
VCA0124	0.05	0.06	0.15	0.13
VCA0125	0.07	0.07	0.14	0.09
VCA0126	0.07	0.07	0.09	0.07
VCA0127	0.04	0.04	0.04	0.05
VCA0128	0.03	0.03	0.06	0.04
VCA0129	0.03	0.03	0.06	0.06
VCA0130	0.06	0.09	0.07	0.07
VCA0131	0.05	0.05	0.06	0.06
VCA0132	0.06	0.08	0.08	0.07
VCA0133	0.03	0.03	0.03	0.04
VCA0134	0.01	0.02	0.04	0.04
VCA0135	0.08	0.09	0.05	0.05
VCA0136	0.03	0.08	0.05	0.03
VCA0137	0.01	0.02	0.06	0.01
VCA0138	0.08	0.09	0.03	0.03
VCA0139	0.06	0.06	0.10	0.09
VCA0140	0.04	0.07	0.08	0.07
VCA0141	0.01	0.01	0.05	0.05
VCA0142	0.06	0.06	0.04	0.05
VCA0143	0.07	0.07	0.05	0.04
VCA0144	0.03	0.05	0.06	0.05
VCA0145	0.05	0.05	0.04	0.05
VCA0146	0.01	0.00	0.03	0.04
VCA0147	0.05	0.07	0.07	0.09
VCA0148	0.08	0.08	0.07	0.04
VCA0149	0.07	0.09	0.04	0.04
VCA0150	0.06	0.07	0.07	0.07
VCA0151	0.04	0.04	0.05	0.05
VCA0152	0.05	0.06	0.09	0.08
VCA0153	0.04	0.06	0.06	0.04
VCA0154	0.06	0.08	0.05	0.05
VCA0155	0.05	0.07	0.07	0.09
VCA0156	0.05	0.07	0.10	0.10
VCA0157	0.04	0.04	0.04	0.06

VCA0158	0.06	0.07	0.05	0.05
VCA0159	0.06	0.06	0.06	0.08
VCA0160	0.04	0.05	0.05	0.05
VCA0161	0.03	0.05	0.06	0.04
VCA0162	0.07	0.07	0.07	0.06
VCA0163	0.08	0.08	0.06	0.05
VCA0164	0.06	0.08	0.06	0.05
VCA0165	-0.04	-0.02	-0.01	0.01
VCA0166	0.06	0.07	0.06	0.04
VCA0167	0.05	0.05	0.03	0.04
VCA0168	0.04	0.05	0.04	0.06
VCA0171	0.07	0.04	0.04	0.04
VCA0172	0.06	0.08	0.09	0.10
VCA0173	0.07	0.07	0.08	0.06
VCA0174	0.05	0.05	0.09	0.08
VCA0175	0.05	0.06	0.07	0.06
VCA0176	0.12	0.12	0.05	0.06
VCA0177	0.06	0.07	0.09	0.06
VCA0178	0.10	0.09	0.05	0.05
VCA0179	0.04	0.05	0.09	0.09
VCA0180	0.06	0.06	0.05	0.05
VCA0181	0.05	0.08	0.06	0.07
VCA0182	0.08	0.09	0.06	0.05
VCA0183	0.07	0.06	0.12	0.07
VCA0184	0.05	0.06	0.06	0.06
VCA0185	0.06	0.07	0.05	0.05
VCA0186	0.05	0.05	0.04	0.03
VCA0187	0.06	0.07	0.06	0.04
VCA0188	0.03	0.05	0.04	0.03
VCA0189	0.04	0.04	0.06	0.03
VCA0190	0.05	0.04	0.03	0.05
VCA0191	0.01	0.04	0.07	0.06
VCA0192	0.00	-0.01	0.05	0.07
VCA0193	0.01	0.01	0.05	0.05
VCA0194	0.00	0.01	0.07	0.06
VCA0195	0.06	0.07	0.02	0.02
VCA0196	0.05	0.05	0.03	0.02
VCA0197	0.04	0.04	0.07	0.05
VCA0199	0.06	0.06	0.07	0.05
VCA0200	0.01	0.01	0.03	0.03
VCA0201	0.01	0.01	0.08	0.08
VCA0202	0.05	0.05	0.05	0.06
VCA0203	0.04	0.05	0.04	-0.09

VCA0204	0.06	0.06	0.08	0.08
VCA0205	0.03	0.05	0.05	0.04
VCA0206	0.10	0.04	0.10	0.07
VCA0207	0.04	0.04	0.09	0.09
VCA0208	0.03	0.03	0.04	0.05
VCA0209	0.05	0.02	0.09	0.07
VCA0210	0.11	0.09	0.27	0.23
VCA0211	0.00	0.01	0.05	0.06
VCA0212	0.00	0.01	0.03	0.04
VCA0213	0.03	0.02	0.06	0.05
VCA0214	0.00	0.01	0.04	0.04
VCA0214	-0.01	0.00	0.04	0.05
VCA0215	0.02	0.10	0.04	0.05
VCA0216	0.01	0.02	0.00	0.05
VCA0216	0.01	0.03	0.04	0.03
VCA0217	0.03	0.04	0.05	0.04
VCA0218	0.02	0.02	0.06	0.06
VCA0219	0.04	0.04	0.06	0.04
VCA0220	0.00	0.01	0.06	0.05
VCA0221	0.03	0.03	0.02	0.02
VCA0222	0.01	0.01	0.06	0.04
VCA0224	0.03	0.04	0.05	0.03
VCA0225	0.07	0.07	0.07	0.09
VCA0226	0.04	0.04	0.06	0.05
VCA0227	0.02	0.03	0.04	0.04
VCA0228	0.01	0.02	0.05	0.05
VCA0229	0.04	0.06	0.04	0.04
VCA0230	0.04	0.06	0.10	0.10
VCA0231	0.03	0.03	0.05	0.04
VCA0232	0.01	0.02	0.05	0.02
VCA0233	-0.02	0.01	0.05	0.05
VCA0233	0.01	0.01	0.06	0.05
VCA0234	0.03	0.04	0.03	0.04
VCA0235	0.05	0.05	0.06	0.06
VCA0236	0.03	0.03	0.04	0.02
VCA0237	0.05	0.01	0.06	0.05
VCA0238	0.03	0.01	0.05	0.05
VCA0239	0.05	0.05	0.10	0.10
VCA0240	0.03	0.03	0.08	0.07
VCA0241	0.02	0.03	0.07	0.07
VCA0242	0.04	0.05	0.13	0.10
VCA0243	0.03	0.04	0.05	0.05
VCA0244	0.05	0.05	0.06	0.06

VCA0245	0.04	0.05	0.04	0.04
VCA0246	0.09	0.09	0.04	0.04
VCA0247	0.00	0.00	0.07	0.06
VCA0248	-0.02	-0.01	0.04	0.02
VCA0249	-0.01	-0.01	0.06	0.04
VCA0250	0.02	0.02	0.06	0.04
VCA0251	0.04	0.06	0.02	0.02
VCA0252	0.05	0.07	0.05	0.06
VCA0253	0.05	0.06	0.04	0.04
VCA0255	0.01	0.02	0.05	0.05
VCA0256	0.07	0.06	0.06	0.06
VCA0258	0.05	0.05	0.05	0.07
VCA0259	0.08	0.06	0.03	0.02
VCA0260	0.07	0.07	0.08	0.04
VCA0262	0.05	0.10	0.04	0.04
VCA0263	0.07	0.05	0.08	0.09
VCA0264	0.02	0.03	0.05	0.05
VCA0265	0.03	0.03	0.05	0.05
VCA0266	0.03	0.13	0.04	0.04
VCA0267	0.02	0.03	0.04	0.03
VCA0268	0.02	0.03	0.04	0.02
VCA0269	0.05	0.06	0.04	0.04
VCA0270	0.03	0.03	0.02	0.08
VCA0271	0.04	0.04	0.05	0.05
VCA0272	0.05	0.05	0.05	0.03
VCA0273	0.06	0.07	0.07	0.02
VCA0274	0.04	0.03	0.04	0.04
VCA0275	0.07	0.08	0.04	0.03
VCA0276	0.05	0.04	0.04	0.04
VCA0277	0.04	0.04	0.06	0.05
VCA0278	0.03	0.05	0.04	0.04
VCA0279	0.05	0.06	0.07	0.07
VCA0280	0.03	0.03	0.03	0.04
VCA0281	0.01	0.02	0.04	0.04
VCA0282	0.02	0.03	0.01	0.03
VCA0283	0.05	0.06	0.04	0.06
VCA0284	0.07	0.07	0.04	0.07
VCA0285	0.07	0.08	0.04	0.03
VCA0286	0.07	0.06	0.04	0.02
VCA0287	0.03	0.02	0.09	0.08
VCA0288	0.08	0.07	0.05	0.06
VCA0289	0.08	0.07	0.04	0.04
VCA0290	0.04	0.05	0.07	0.07

VCA0291	0.06	0.07	0.05	0.05
VCA0292	0.07	0.05	0.04	0.04
VCA0294	0.07	0.07	0.05	0.04
VCA0295	0.07	0.06	0.02	0.05
VCA0296	0.10	0.10	0.03	0.05
VCA0297	0.07	0.06	0.02	0.03
VCA0298	0.07	0.07	0.08	0.08
VCA0299	0.10	0.09	0.07	0.06
VCA0300	0.09	0.07	0.05	0.02
VCA0301	0.09	0.07	0.10	0.09
VCA0303	0.07	0.08	0.05	0.04
VCA0304	0.08	0.07	0.14	0.16
VCA0305	0.01	0.03	0.06	0.06
VCA0306	0.07	0.07	0.06	0.06
VCA0307	0.03	0.02	0.05	0.06
VCA0307	-0.01	0.00	0.08	0.07
VCA0308	0.03	0.02	0.09	0.07
VCA0308	0.01	0.00	0.05	0.05
VCA0309	0.10	0.09	0.09	0.10
VCA0310	0.07	0.07	0.04	0.04
VCA0312	0.08	0.06	0.05	0.04
VCA0313	0.11	0.09	0.06	0.03
VCA0314	0.10	0.08	0.06	0.06
VCA0315	0.07	0.06	0.07	0.05
VCA0316	0.07	0.06	0.06	0.06
VCA0317	0.08	0.07	0.03	0.04
VCA0319	0.07	0.07	0.04	0.07
VCA0320	0.06	0.08	0.06	0.08
VCA0321	0.09	0.08	0.04	0.07
VCA0322	0.13	0.10	0.05	0.04
VCA0323	0.04	0.04	0.00	0.01
VCA0324	0.01	0.01	0.07	0.07
VCA0326	0.06	0.08	0.03	0.04
VCA0327	0.02	0.02	0.08	0.07
VCA0328	0.00	0.01	0.14	0.12
VCA0329	0.04	0.06	0.08	0.07
VCA0330	0.01	0.01	0.07	0.06
VCA0331	0.03	0.03	0.04	0.04
VCA0333	0.07	0.06	0.07	0.05
VCA0334	0.02	0.02	0.06	0.04
VCA0335	0.06	0.06	0.01	0.02
VCA0336	0.05	0.06	0.04	0.04
VCA0337	0.02	0.01	0.13	0.08

VCA0338	0.02	0.02	0.03	0.05
VCA0338	0.01	0.02	0.04	0.06
VCA0339	0.04	0.06	0.03	0.04
VCA0340	-0.01	-0.01	0.18	0.14
VCA0341	0.02	0.02	0.04	0.04
VCA0341	-0.02	-0.01	0.05	0.06
VCA0342	0.02	0.01	0.11	0.09
VCA0343	0.06	0.05	0.05	0.03
VCA0344	0.04	0.04	0.04	0.02
VCA0345	0.00	0.00	0.10	0.07
VCA0346	0.13	0.01	0.02	0.02
VCA0347	0.03	0.04	0.05	0.06
VCA0349	0.01	0.02	0.03	0.05
VCA0349	0.01	0.03	0.04	0.05
VCA0350	0.05	0.07	0.05	0.02
VCA0351	0.00	0.00	0.08	0.08
VCA0352	0.02	0.03	0.00	0.03
VCA0353	0.05	0.06	0.06	0.05
VCA0354	0.03	0.04	0.03	0.02
VCA0355	0.11	0.04	0.06	0.14
VCA0356	0.02	0.02	0.08	0.08
VCA0357	0.05	0.05	0.05	0.02
VCA0358	0.02	0.01	0.06	0.07
VCA0359	0.08	0.05	0.04	0.03
VCA0360	0.09	0.10	0.04	0.03
VCA0361	0.11	0.10	0.03	0.04
VCA0362	0.06	0.07	0.03	0.03
VCA0363	0.06	0.05	0.05	0.07
VCA0364	0.07	0.07	0.03	0.03
VCA0366	0.07	0.07	0.08	0.07
VCA0367	0.06	0.05	0.04	0.09
VCA0368	0.06	0.06	0.03	0.07
VCA0369	0.07	0.07	0.07	0.05
VCA0370	0.06	0.06	0.09	0.12
VCA0371	0.03	0.02	0.09	0.08
VCA0372	0.13	0.12	0.06	0.06
VCA0373	0.06	0.07	0.05	0.05
VCA0374	0.01	0.03	0.05	0.04
VCA0376	0.07	0.08	0.02	0.02
VCA0379	0.07	0.09	0.07	0.10
VCA0379	0.02	0.03	0.04	0.05
VCA0380	0.03	0.03	0.04	0.05
VCA0381	0.04	0.07	0.03	0.02

VCA0382	0.06	0.05	0.10	0.08
VCA0383	0.06	0.07	0.06	0.06
VCA0384	0.06	0.06	0.03	0.02
VCA0386	0.03	0.04	0.10	0.13
VCA0387	0.03	0.03	0.01	0.02
VCA0388	0.04	0.07	0.03	0.09
VCA0389	0.02	0.03	0.04	0.03
VCA0390	0.06	0.11	0.02	0.02
VCA0391	0.03	0.03	0.06	0.07
VCA0392	0.04	0.04	0.13	0.09
VCA0393	0.04	0.03	0.04	0.02
VCA0394	0.08	0.09	0.02	0.02
VCA0395	0.03	0.02	0.04	0.04
VCA0396	0.04	0.01	0.03	0.08
VCA0397	0.05	0.05	0.05	0.05
VCA0398	0.05	0.10	0.06	0.06
VCA0400	0.01	0.02	0.10	0.06
VCA0401	0.03	0.03	0.05	0.09
VCA0401	0.01	0.04	0.04	0.04
VCA0402	0.17	0.19	0.03	0.03
VCA0403	0.03	0.04	0.05	0.03
VCA0404	0.10	0.08	0.05	0.07
VCA0405	0.03	0.06	0.06	0.07
VCA0406	0.03	0.04	0.05	0.06
VCA0407	0.05	0.06	0.04	0.03
VCA0408	0.06	0.05	0.15	0.11
VCA0409	0.10	0.09	0.03	0.03
VCA0410	0.12	0.07	0.07	0.09
VCA0411	0.06	0.06	0.06	0.03
VCA0412	0.04	0.05	0.05	0.05
VCA0413	0.04	0.06	0.07	0.07
VCA0414	0.02	0.02	0.05	0.04
VCA0415	0.06	0.08	0.08	0.08
VCA0417	0.07	0.06	0.09	0.08
VCA0418	0.04	0.05	0.06	0.05
VCA0419	0.04	0.05	0.04	0.03
VCA0420	0.04	0.02	0.05	0.04
VCA0421	0.06	0.05	0.04	0.04
VCA0422	0.10	0.06	0.06	0.05
VCA0423	0.03	0.04	0.05	0.06
VCA0424	0.02	0.02	0.02	0.03
VCA0425	0.04	0.03	0.06	0.04
VCA0426	0.05	0.05	0.05	0.05

VCA0427	0.05	0.06	0.06	0.04
VCA0428	0.05	0.07	0.07	0.06
VCA0429	0.05	0.06	0.05	0.06
VCA0430	0.04	0.06	0.06	0.05
VCA0431	0.03	0.04	0.05	0.04
VCA0432	0.05	0.05	0.10	0.09
VCA0433	0.15	0.03	0.03	0.02
VCA0434	0.05	0.05	0.07	0.06
VCA0435	0.05	0.04	0.06	0.06
VCA0436	0.03	0.03	0.07	0.08
VCA0437	0.04	0.05	0.04	0.02
VCA0437	0.05	0.05	0.06	0.08
VCA0438	0.05	0.07	0.02	0.02
VCA0439	0.04	0.04	0.06	0.07
VCA0440	0.04	0.04	0.04	0.02
VCA0441	0.03	0.04	0.08	0.08
VCA0442	0.03	0.03	0.04	0.03
VCA0443	0.01	0.03	0.07	0.06
VCA0445	0.04	0.04	0.05	0.03
VCA0446	0.06	0.06	0.05	0.05
VCA0447	0.02	0.04	0.04	0.04
VCA0448	0.02	0.03	0.06	0.02
VCA0449	0.08	0.09	0.05	0.04
VCA0450	0.03	0.03	0.06	0.04
VCA0451	0.02	0.04	0.07	0.03
VCA0453	0.08	0.07	0.09	0.06
VCA0454	0.04	0.04	0.13	0.13
VCA0455	0.02	0.02	0.04	0.05
VCA0456	0.07	0.07	0.03	0.03
VCA0457	0.02	0.03	0.04	0.03
VCA0458	0.08	0.06	0.03	0.03
VCA0459	0.04	0.04	0.02	0.02
VCA0460	0.03	0.03	0.05	0.09
VCA0461	0.07	0.08	0.05	0.06
VCA0462	0.06	0.07	0.04	0.03
VCA0463	0.04	0.05	0.07	0.06
VCA0464	0.03	0.06	0.04	0.04
VCA0465	0.02	0.03	0.05	0.04
VCA0466	0.06	0.06	0.07	0.06
VCA0467	0.06	0.06	0.06	0.05
VCA0468	0.06	0.06	0.06	0.05
VCA0469	0.05	0.08	0.03	0.04
VCA0470	0.05	0.06	0.06	0.05

VCA0471	0.06	0.07	0.05	0.04
VCA0472	0.03	0.03	0.10	0.09
VCA0473	0.06	0.09	0.07	0.07
VCA0474	0.06	0.07	0.07	0.08
VCA0476	0.05	0.06	0.07	0.08
VCA0477	0.02	0.04	0.04	0.05
VCA0479	0.04	0.05	0.06	0.04
VCA0481	0.01	0.03	-0.02	-0.03
VCA0482	0.02	0.02	0.06	0.07
VCA0483	0.04	0.06	0.06	0.05
VCA0484	0.07	0.07	0.06	0.06
VCA0485	0.03	0.03	0.05	0.06
VCA0487	0.04	0.05	0.04	0.04
VCA0488	0.03	0.06	0.06	0.06
VCA0490	0.02	0.05	0.04	0.04
VCA0491	0.03	0.05	0.04	0.04
VCA0492	0.05	0.05	0.07	0.05
VCA0493	0.04	0.04	0.06	0.05
VCA0494	0.07	0.06	0.09	0.06
VCA0495	0.05	0.06	0.05	0.05
VCA0496	0.09	0.07	0.05	0.09
VCA0498	0.05	0.09	0.06	0.06
VCA0499	0.06	0.06	0.05	0.06
VCA0500	0.07	0.08	0.09	0.07
VCA0501	0.01	0.02	0.05	0.05
VCA0502	0.04	0.06	0.05	0.06
VCA0505	0.03	0.06	0.06	0.04
VCA0506	0.03	0.03	0.07	0.05
VCA0507	-0.01	0.00	0.03	0.05
VCA0508	0.01	0.03	0.05	0.05
VCA0509	0.02	0.03	0.05	0.04
VCA0510	0.03	0.03	0.07	0.05
VCA0511	0.04	0.04	0.07	0.06
VCA0512	0.04	0.05	0.04	0.06
VCA0513	0.03	0.03	0.05	0.07
VCA0514	0.06	0.08	0.03	0.02
VCA0515	0.08	0.10	0.03	0.03
VCA0516	0.02	0.01	0.06	0.08
VCA0517	-0.01	-0.01	0.04	0.06
VCA0518	0.03	0.03	0.07	0.08
VCA0519	0.00	0.01	0.05	0.04
VCA0520	0.03	0.01	0.02	0.04
VCA0521	0.09	0.05	0.07	0.08

VCA0522	0.09	0.08	0.07	0.06
VCA0523	0.03	0.03	0.06	0.06
VCA0524	0.07	0.06	0.03	0.03
VCA0525	0.09	0.09	0.00	0.01
VCA0526	0.03	0.05	0.02	0.02
VCA0527	0.03	0.02	0.08	0.08
VCA0528	0.03	0.03	0.06	0.09
VCA0529	0.00	0.01	0.06	0.05
VCA0530	0.03	0.04	0.04	0.05
VCA0531	0.05	0.04	0.05	0.04
VCA0532	0.04	0.05	0.04	0.03
VCA0533	0.02	0.02	0.04	0.06
VCA0534	0.02	0.05	0.05	0.05
VCA0535	0.04	0.04	0.06	0.06
VCA0538	0.03	0.03	0.07	0.07
VCA0539	0.03	0.04	0.17	0.12
VCA0540	0.00	0.00	0.05	0.05
VCA0541	0.09	0.08	0.03	0.03
VCA0542	0.03	0.02	0.06	0.05
VCA0543	0.02	0.03	0.09	0.08
VCA0546	0.04	0.05	0.15	0.14
VCA0547	0.09	0.08	0.04	0.04
VCA0548	0.07	0.08	0.01	0.05
VCA0549	0.05	0.05	0.15	0.14
VCA0550	0.01	0.01	0.15	0.14
VCA0551	0.04	0.02	0.03	0.04
VCA0552	0.05	0.05	0.07	0.07
VCA0553	0.04	0.03	0.05	0.04
VCA0554	0.02	0.00	0.04	0.04
VCA0555	0.04	0.06	0.01	0.02
VCA0556	0.04	0.02	0.04	0.03
VCA0557	-0.01	-0.01	-0.02	-0.01
VCA0558	0.03	0.05	0.03	0.03
VCA0559	-0.01	-0.01	0.03	0.03
VCA0560	0.01	0.00	0.06	0.06
VCA0561	0.07	0.10	0.05	0.05
VCA0562	0.05	0.06	0.04	0.05
VCA0563	0.02	0.03	0.04	0.04
VCA0564	0.02	0.02	0.04	0.05
VCA0565	0.02	0.03	0.06	0.06
VCA0566	0.06	0.07	0.04	0.02
VCA0567	0.04	0.04	0.08	0.12
VCA0568	0.06	0.07	0.05	0.05

VCA0569	0.08	0.07	0.04	0.05
VCA0570	0.08	0.08	0.07	0.04
VCA0571	0.04	0.04	0.02	0.09
VCA0572	0.05	0.06	0.06	0.05
VCA0573	0.07	0.07	0.06	0.05
VCA0574	0.04	0.04	0.05	0.05
VCA0575	0.11	0.11	0.08	0.07
VCA0576	0.02	0.03	0.04	0.05
VCA0577	0.09	0.08	0.03	0.02
VCA0578	0.04	0.05	0.03	0.04
VCA0579	0.07	0.05	0.06	0.03
VCA0580	0.03	0.03	0.02	0.04
VCA0581	0.03	0.03	0.03	0.06
VCA0582	0.04	0.06	0.06	0.06
VCA0583	0.04	0.03	0.04	0.02
VCA0584	0.08	0.06	0.05	0.05
VCA0585	0.04	0.05	0.02	0.04
VCA0586	0.07	0.05	0.08	0.11
VCA0587	0.08	0.08	0.07	0.06
VCA0588	0.06	0.05	0.05	0.04
VCA0589	0.02	0.02	0.00	0.01
VCA0590	0.02	0.03	0.04	0.03
VCA0591	0.04	0.06	0.04	0.06
VCA0592	0.06	0.07	0.04	0.04
VCA0593	0.56	0.55	0.58	0.52
VCA0594	0.04	0.05	0.05	0.06
VCA0595	0.06	0.07	0.08	0.05
VCA0596	0.06	0.06	0.06	0.05
VCA0597	0.04	0.05	0.02	0.02
VCA0598	0.05	0.06	0.06	0.04
VCA0599	0.05	0.07	0.07	0.07
VCA0600	0.10	0.08	0.06	0.06
VCA0601	0.03	0.03	0.05	0.08
VCA0602	0.00	0.03	0.04	0.03
VCA0603	0.06	0.09	0.11	0.07
VCA0604	0.04	0.05	0.03	0.04
VCA0605	0.02	0.01	0.06	0.06
VCA0606	0.13	0.06	0.08	0.09
VCA0607	0.09	0.09	0.07	0.06
VCA0608	0.03	0.04	0.04	0.04
VCA0609	0.05	0.06	0.04	0.05
VCA0610	0.04	0.07	0.05	0.05
VCA0611	0.06	0.07	0.02	0.04

VCA0612	0.05	0.07	0.03	0.03
VCA0613	0.07	0.07	0.05	0.04
VCA0614	0.05	0.06	0.06	0.09
VCA0615	0.03	0.06	0.04	0.06
VCA0616	0.04	0.04	0.04	0.05
VCA0617	0.06	0.07	0.05	0.05
VCA0618	0.08	0.04	0.05	0.05
VCA0619	0.04	0.02	0.08	0.05
VCA0620	0.06	0.06	0.11	0.09
VCA0621	0.10	0.11	0.05	0.11
VCA0622	0.07	0.06	0.05	0.05
VCA0623	0.09	0.08	0.08	0.06
VCA0624	0.04	0.04	0.05	0.16
VCA0626	0.06	0.06	0.09	0.06
VCA0627	0.05	0.05	0.05	0.05
VCA0628	0.01	0.02	0.03	0.06
VCA0629	0.05	0.05	0.04	0.04
VCA0630	0.09	0.07	0.13	0.11
VCA0631	0.08	0.10	0.10	0.08
VCA0632	0.07	0.08	0.03	0.04
VCA0633	0.03	0.03	0.08	0.06
VCA0634	0.10	0.08	0.07	0.09
VCA0635	0.07	0.08	0.09	0.07
VCA0636	0.05	0.06	0.07	0.08
VCA0637	0.05	0.06	0.06	0.07
VCA0638	0.02	0.03	0.04	0.04
VCA0639	0.08	0.05	0.07	0.04
VCA0640	0.07	0.10	0.09	0.09
VCA0641	0.08	0.06	0.10	0.13
VCA0642	0.08	0.07	0.09	0.08
VCA0644	0.06	0.06	0.11	0.05
VCA0645	0.06	0.10	0.08	0.07
VCA0646	0.05	0.06	0.04	0.05
VCA0647	0.07	0.07	0.10	0.09
VCA0648	0.05	0.05	0.04	0.03
VCA0649	0.08	0.08	0.11	0.08
VCA0650	0.05	0.06	0.04	0.05
VCA0651	0.05	0.04	0.06	0.04
VCA0652	0.07	0.06	0.06	0.07
VCA0653	0.05	0.04	0.07	0.05
VCA0654	0.04	0.07	0.13	0.10
VCA0655	0.07	0.07	0.05	0.05
VCA0656	0.06	0.05	0.07	0.08

VCA0657	0.10	0.10	0.03	0.03
VCA0658	0.05	0.04	0.05	0.05
VCA0659	0.06	0.05	0.06	0.08
VCA0660	0.05	0.06	0.06	0.05
VCA0661	0.06	0.05	0.06	0.07
VCA0662	0.06	0.05	0.06	0.06
VCA0663	0.04	0.03	0.04	0.03
VCA0664	0.05	0.04	0.04	0.07
VCA0665	0.06	0.07	0.07	0.07
VCA0666	0.05	0.06	0.04	0.02
VCA0667	0.05	0.05	0.05	0.04
VCA0668	0.04	0.04	0.03	0.03
VCA0669	0.05	0.06	0.07	0.07
VCA0670	0.08	0.09	0.14	0.16
VCA0671	0.03	0.03	0.10	0.10
VCA0672	0.05	0.06	0.02	0.03
VCA0673	0.05	0.04	0.06	0.06
VCA0674	0.02	0.02	0.07	0.06
VCA0675	0.04	0.04	0.03	0.03
VCA0676	0.04	0.06	0.05	0.05
VCA0677	0.05	0.03	0.09	0.09
VCA0678	0.02	0.02	0.04	0.06
VCA0678	0.01	0.01	0.05	0.04
VCA0679	0.06	0.07	0.08	0.07
VCA0680	0.06	0.05	0.07	0.06
VCA0681	0.54	0.62	0.61	0.62
VCA0682	0.06	0.06	0.04	0.05
VCA0683	0.06	0.06	0.07	0.05
VCA0684	0.03	0.04	0.07	0.07
VCA0685	0.07	0.06	0.08	0.12
VCA0686	0.05	0.06	0.05	0.05
VCA0687	0.04	0.05	0.09	0.08
VCA0688	0.07	0.06	0.03	0.03
VCA0689	0.04	0.04	0.11	0.10
VCA0690	0.04	0.04	0.08	0.07
VCA0691	0.05	0.05	0.07	0.14
VCA0692	0.01	0.04	0.02	0.04
VCA0693	0.00	0.01	0.02	0.02
VCA0694	0.06	0.05	0.08	0.06
VCA0695	0.04	0.03	0.07	0.11
VCA0696	0.07	0.04	0.03	0.03
VCA0696	0.03	0.03	0.02	0.03
VCA0697	0.01	0.01	0.10	0.09

VCA0698	0.07	0.06	0.03	0.03
VCA0699	0.05	0.05	0.08	0.06
VCA0700	0.05	0.05	0.05	0.05
VCA0701	0.05	0.06	0.06	0.06
VCA0702	0.09	0.10	0.11	0.09
VCA0703	0.05	0.04	0.06	0.04
VCA0704	0.05	0.04	0.08	0.07
VCA0705	0.03	0.04	0.03	0.04
VCA0706	0.03	0.03	0.05	0.05
VCA0707	0.03	0.03	0.13	0.12
VCA0708	0.05	0.04	0.06	0.04
VCA0709	0.02	0.02	0.03	0.03
VCA0710	0.08	0.07	0.05	0.06
VCA0711	0.08	0.05	0.04	0.07
VCA0712	0.10	0.09	0.07	0.06
VCA0713	0.06	0.05	0.07	0.07
VCA0714	0.08	0.07	0.05	0.05
VCA0715	0.04	0.05	0.06	0.07
VCA0716	0.04	0.02	0.07	0.05
VCA0717	0.03	0.05	0.04	0.14
VCA0718	0.07	0.08	0.03	0.03
VCA0719	0.12	0.13	0.05	0.05
VCA0720	0.01	0.01	0.14	0.12
VCA0721	0.06	0.07	0.04	0.06
VCA0722	0.12	0.14	0.06	0.05
VCA0723	0.04	0.02	0.06	0.05
VCA0724	0.03	0.04	0.02	0.02
VCA0724	0.01	0.05	0.07	0.04
VCA0725	0.05	0.06	0.06	0.06
VCA0726	0.04	0.08	0.04	0.03
VCA0727	0.04	0.06	0.04	0.05
VCA0728	0.05	0.06	0.06	0.05
VCA0729	0.10	0.07	0.06	0.06
VCA0730	0.04	0.04	0.05	0.05
VCA0730	0.01	-0.02	0.08	0.04
VCA0731	0.14	0.12	0.03	0.03
VCA0732	0.03	0.03	0.07	0.04
VCA0733	0.09	0.09	0.06	0.07
VCA0734	0.08	0.08	0.05	0.06
VCA0735	0.12	0.13	0.23	0.24
VCA0736	0.01	0.02	0.04	0.05
VCA0737	0.14	0.11	0.02	0.02
VCA0738	0.05	0.06	0.02	0.01

VCA0739	0.15	0.16	0.05	0.04
VCA0740	0.15	0.13	-0.01	0.01
VCA0741	0.09	0.08	0.05	0.04
VCA0742	0.06	0.06	0.03	0.05
VCA0743	0.13	0.12	0.02	0.01
VCA0744	0.11	0.12	0.12	0.13
VCA0745	0.02	0.03	0.04	0.03
VCA0746	0.12	0.11	0.04	0.01
VCA0747	0.07	0.05	0.04	0.03
VCA0748	0.12	0.12	0.02	0.04
VCA0749	0.12	0.09	0.02	0.01
VCA0750	0.07	0.07	0.05	0.05
VCA0750	0.07	0.07	0.03	0.05
VCA0751	0.09	0.09	-0.01	0.01
VCA0752	0.13	0.12	-0.01	0.00
VCA0753	0.08	0.06	0.04	0.04
VCA0754	0.08	0.07	0.00	0.01
VCA0755	0.10	0.09	0.03	0.02
VCA0756	0.10	0.10	0.00	0.02
VCA0757	0.12	0.12	0.01	0.01
VCA0758	0.12	0.09	0.00	0.01
VCA0759	0.13	0.11	0.01	0.00
VCA0760	0.15	0.12	0.01	0.01
VCA0761	0.12	0.09	0.02	0.02
VCA0762	0.05	0.06	0.05	0.06
VCA0763	0.10	0.10	0.07	0.07
VCA0764	0.06	0.05	0.05	0.05
VCA0765	0.13	0.10	0.03	0.01
VCA0766	0.15	0.13	-0.01	-0.01
VCA0767	0.08	0.09	0.03	0.02
VCA0768	0.13	0.13	0.02	0.02
VCA0769	0.12	0.11	0.00	0.00
VCA0770	0.06	0.05	0.06	0.05
VCA0770	0.06	0.06	0.04	0.05
VCA0771	0.06	0.07	0.05	0.04
VCA0772	0.10	0.07	0.02	0.02
VCA0773	0.09	0.10	0.08	0.08
VCA0774	0.05	0.04	0.04	0.04
VCA0775	0.03	0.03	0.06	0.07
VCA0776	0.02	0.02	0.05	0.05
VCA0777	0.06	0.06	0.06	0.04
VCA0778	0.09	0.07	0.08	0.05
VCA0779	0.04	0.03	0.01	0.01

VCA0780	0.08	0.09	0.03	0.04
VCA0781	0.05	0.06	0.03	0.03
VCA0782	0.04	0.05	0.08	0.06
VCA0783	0.05	0.04	0.04	0.03
VCA0784	0.09	0.06	0.06	0.06
VCA0785	0.04	0.05	0.00	-0.01
VCA0786	0.07	0.06	0.02	0.02
VCA0787	0.06	0.06	0.03	0.04
VCA0788	0.07	0.08	0.06	0.06
VCA0789	0.10	0.09	0.05	0.04
VCA0790	0.06	0.07	0.02	0.03
VCA0791	0.06	0.05	0.06	0.06
VCA0792	0.05	0.03	0.04	0.05
VCA0793	0.05	0.09	0.10	0.08
VCA0794	0.04	0.07	0.07	0.05
VCA0795	0.06	0.06	0.07	0.05
VCA0796	0.09	0.10	0.04	0.05
VCA0796	0.06	0.07	0.08	0.07
VCA0797	0.06	0.11	0.08	0.07
VCA0798	0.04	0.05	0.11	0.10
VCA0799	0.07	0.06	0.07	0.04
VCA0800	0.07	0.06	0.11	0.08
VCA0801	0.08	0.08	0.05	0.04
VCA0802	0.03	0.04	0.02	0.04
VCA0803	0.02	0.02	0.04	0.05
VCA0804	0.04	0.04	0.05	0.01
VCA0805	0.04	0.04	-0.02	-0.02
VCA0806	0.07	0.05	0.06	0.04
VCA0807	0.06	0.07	0.06	0.06
VCA0808	0.06	0.05	0.03	0.02
VCA0809	0.04	0.04	0.02	0.02
VCA0810	0.05	0.05	0.04	0.05
VCA0810	0.05	0.06	0.04	0.05
VCA0811	0.06	0.07	0.03	0.05
VCA0812	0.16	0.17	0.06	0.06
VCA0813	0.08	0.08	0.06	0.06
VCA0814	0.06	0.07	0.08	0.08
VCA0815	0.05	0.06	0.03	0.03
VCA0815	-0.03	0.00	0.02	0.04
VCA0816	0.05	0.08	0.04	0.05
VCA0816	0.05	0.07	0.04	0.05
VCA0817	0.05	0.06	0.06	0.07
VCA0819	0.09	0.09	0.06	0.05

VCA0820	0.02	0.02	0.09	0.08
VCA0821	0.05	0.05	0.04	0.05
VCA0822	0.09	0.10	0.01	0.05
VCA0823	0.07	0.06	0.05	0.05
VCA0824	0.01	0.03	0.04	0.05
VCA0824	0.01	0.03	0.04	0.04
VCA0825	0.06	0.07	0.05	0.08
VCA0826	0.06	0.07	0.05	0.04
VCA0826	0.06	0.06	0.04	0.04
VCA0827	0.12	0.11	0.05	0.05
VCA0828	0.05	0.06	0.08	0.09
VCA0830	0.01	0.01	0.04	0.04
VCA0831	0.03	0.04	0.06	0.06
VCA0832	0.06	0.03	0.06	0.07
VCA0833	0.05	0.04	0.05	0.06
VCA0834	0.05	0.03	0.08	0.06
VCA0835	0.03	0.05	0.03	0.05
VCA0836	0.03	0.02	0.04	0.06
VCA0836	0.01	0.01	0.05	0.05
VCA0837	0.05	0.05	0.09	0.13
VCA0838	0.06	0.05	0.08	0.07
VCA0839	0.02	0.02	0.06	0.07
VCA0840	0.10	0.10	0.06	0.08
VCA0841	0.05	0.06	0.09	0.09
VCA0842	0.05	0.05	0.03	0.03
VCA0842	0.05	0.06	0.05	0.05
VCA0843	0.11	0.10	0.09	0.09
VCA0844	0.05	0.06	0.03	0.03
VCA0845	0.04	0.05	0.05	0.05
VCA0846	0.08	0.06	0.06	0.08
VCA0847	0.01	0.02	0.04	0.04
VCA0848	0.01	0.01	-0.01	0.02
VCA0850	0.04	0.04	0.06	0.11
VCA0851	0.08	0.07	0.05	0.08
VCA0852	0.03	0.06	0.04	0.07
VCA0853	0.11	0.09	0.04	0.04
VCA0854	0.05	0.04	0.07	0.07
VCA0855	0.08	0.09	0.06	0.03
VCA0855	0.06	0.06	0.07	0.05
VCA0856	0.05	0.07	0.06	0.08
VCA0857	0.05	0.06	0.08	0.07
VCA0858	0.04	0.05	0.05	0.05
VCA0861	0.06	0.06	0.03	0.04

VCA0862	0.06	0.06	0.04	0.05
VCA0863	0.07	0.06	0.05	0.05
VCA0864	0.04	0.01	0.03	0.04
VCA0865	0.03	0.04	0.08	0.06
VCA0866	0.03	0.03	0.04	0.04
VCA0867	0.02	0.03	0.02	0.02
VCA0868	0.06	0.07	0.02	0.03
VCA0869	0.05	0.06	0.05	0.05
VCA0870	0.05	0.05	0.08	0.08
VCA0871	0.05	0.03	0.02	0.02
VCA0872	0.02	0.01	0.03	0.05
VCA0873	0.05	0.07	0.03	0.03
VCA0874	0.05	0.06	0.10	0.11
VCA0875	0.05	0.05	0.07	0.06
VCA0876	0.03	0.04	0.10	0.10
VCA0878	0.07	0.09	0.04	0.04
VCA0879	0.04	0.07	0.03	0.04
VCA0879	0.07	0.07	0.06	0.05
VCA0880	0.08	0.06	0.04	0.03
VCA0882	0.04	0.05	0.05	0.04
VCA0883	0.12	0.13	0.05	0.08
VCA0884	0.03	0.03	0.04	0.05
VCA0885	0.07	0.05	0.07	0.07
VCA0886	0.07	0.07	0.08	0.08
VCA0887	0.06	0.06	0.09	0.06
VCA0888	0.07	0.05	0.06	0.04
VCA0889	0.05	0.09	0.11	0.09
VCA0890	0.08	0.06	0.09	0.11
VCA0891	0.05	0.04	0.02	0.03
VCA0892	0.08	0.09	0.04	0.03
VCA0893	0.09	0.09	0.04	0.05
VCA0894	0.06	0.08	0.08	0.07
VCA0895	0.06	0.05	0.24	0.27
VCA0896	0.04	0.05	0.05	0.05
VCA0897	0.08	0.08	0.03	0.05
VCA0898	0.05	0.05	0.05	0.07
VCA0899	0.06	0.06	0.04	0.04
VCA0900	0.06	0.05	-0.03	-0.02
VCA0901	0.03	0.03	0.06	0.06
VCA0902	0.04	0.03	0.08	0.06
VCA0903	0.07	0.05	0.09	0.08
VCA0904	0.06	0.03	0.10	0.09
VCA0905	0.05	0.04	0.04	0.04

VCA0906	0.04	0.04	0.05	0.04
VCA0907	0.06	0.04	0.10	0.10
VCA0908	0.02	0.04	0.04	0.04
VCA0909	0.04	0.04	0.05	0.06
VCA0910	0.03	0.06	0.07	0.06
VCA0911	0.08	0.08	0.03	0.03
VCA0912	0.03	0.04	0.04	0.05
VCA0913	0.04	0.09	0.07	0.06
VCA0914	0.04	0.04	0.04	0.04
VCA0915	0.05	0.04	0.04	0.05
VCA0916	0.03	0.05	0.06	0.05
VCA0917	0.11	0.07	0.07	0.08
VCA0918	0.03	0.03	0.03	0.03
VCA0918	0.03	0.06	0.05	0.06
VCA0919	0.06	0.05	0.11	0.09
VCA0920	0.03	0.04	0.04	0.09
VCA0921	0.09	0.08	0.01	0.01
VCA0922	0.05	0.06	0.07	0.05
VCA0923	-0.01	-0.01	0.06	0.05
VCA0924	-0.01	-0.01	0.01	0.00
VCA0925	0.07	0.07	0.08	0.06
VCA0926	0.04	0.07	0.05	0.03
VCA0927	0.00	0.01	0.08	0.06
VCA0928	0.00	0.00	0.07	0.04
VCA0929	0.04	0.07	0.00	0.01
VCA0930	0.02	0.02	0.05	0.04
VCA0931	0.47	0.48	0.35	0.30
VCA0932	0.06	0.06	0.07	0.06
VCA0933	0.04	0.05	0.08	0.08
VCA0934	0.05	0.06	0.04	0.04
VCA0935	0.04	0.05	0.05	0.04
VCA0936	0.03	0.03	0.09	0.08
VCA0937	0.05	0.05	0.06	0.06
VCA0938	0.03	0.03	0.05	0.04
VCA0939	0.03	0.04	-0.01	-0.02
VCA0940	-0.01	0.02	0.04	0.07
VCA0941	0.07	0.08	0.06	0.08
VCA0942	0.07	0.06	0.03	0.03
VCA0943	0.03	0.03	0.04	0.04
VCA0944	0.04	0.03	0.04	0.05
VCA0945	0.02	0.02	0.02	0.19
VCA0946	0.05	0.05	0.03	0.04
VCA0947	0.01	0.01	0.06	0.23

VCA0948	0.08	0.07	0.03	0.04
VCA0949	0.05	0.06	0.02	0.02
VCA0950	0.08	0.07	0.02	0.04
VCA0951	0.02	0.01	0.03	0.04
VCA0952	0.07	0.07	0.10	0.08
VCA0953	0.04	0.06	0.02	0.04
VCA0954	0.05	0.06	0.05	0.05
VCA0955	0.00	0.00	0.03	0.07
VCA0956	0.00	-0.02	-0.01	0.00
VCA0957	0.06	0.06	0.04	0.03
VCA0958	0.04	0.04	0.05	0.05
VCA0959	0.12	0.07	0.06	0.05
VCA0960	0.05	0.03	0.06	0.08
VCA0961	0.04	0.05	0.03	0.05
VCA0962	0.05	0.04	0.05	0.06
VCA0963	0.01	0.01	0.16	0.04
VCA0964	0.01	0.02	0.04	0.03
VCA0965	0.00	0.01	0.11	0.14
VCA0966	0.03	0.02	0.05	0.06
VCA0967	0.06	0.07	0.03	0.04
VCA0968	0.07	0.07	0.07	0.06
VCA0969	0.07	0.08	0.05	0.07
VCA0970	0.03	0.03	0.04	0.07
VCA0971	0.03	0.03	0.03	0.05
VCA0972	0.05	0.06	0.07	0.09
VCA0973	0.07	0.08	0.07	0.05
VCA0974	0.02	0.03	0.04	0.03
VCA0975	0.03	0.03	0.05	0.05
VCA0976	0.01	0.02	0.04	0.04
VCA0977	0.04	0.05	0.06	0.05
VCA0979	0.02	0.04	0.06	0.04
VCA0980	0.02	0.00	0.04	0.05
VCA0981	0.04	0.04	0.05	0.04
VCA0982	0.01	0.01	0.07	0.05
VCA0983	0.03	0.03	0.04	0.05
VCA0984	0.03	0.07	0.06	0.04
VCA0985	0.02	0.02	0.04	0.04
VCA0986	0.02	0.02	0.06	0.05
VCA0987	0.03	0.05	0.04	0.06
VCA0988	0.03	0.03	0.04	0.05
VCA0989	0.03	0.04	0.04	0.04
VCA0990	0.03	0.03	0.07	0.07
VCA0991	0.07	0.11	0.05	0.06

VCA0992	0.04	0.07	0.09	0.10
VCA0993	0.06	0.06	0.09	0.09
VCA0994	0.05	0.07	0.07	0.06
VCA0995	0.05	0.08	0.07	0.06
VCA0996	0.04	0.04	0.04	0.03
VCA0997	0.04	0.04	0.06	0.05
VCA0998	0.06	0.05	0.09	0.07
VCA0999	0.05	0.07	0.10	0.09
VCA1000	0.06	0.07	0.03	0.02
VCA1001	0.08	0.11	0.06	0.05
VCA1002	0.03	0.04	0.02	0.02
VCA1003	0.05	0.07	0.08	0.06
VCA1004	0.03	0.04	0.07	0.06
VCA1005	0.02	0.05	0.06	0.05
VCA1006	0.04	0.04	0.09	0.07
VCA1007	0.05	0.07	0.06	0.07
VCA1008	0.02	0.03	0.06	0.07
VCA1009	0.05	0.06	0.07	0.04
VCA1009	0.06	0.06	0.04	0.04
VCA1010	0.12	0.13	0.06	0.07
VCA1011	0.03	0.07	0.06	0.05
VCA1012	0.07	0.12	0.06	0.04
VCA1013	0.08	0.16	0.13	0.10
VCA1014	0.04	0.13	0.03	0.03
VCA1014	0.06	0.07	0.04	0.05
VCA1015	0.03	0.02	0.09	0.09
VCA1016	0.04	0.06	0.03	0.02
VCA1017	0.03	0.04	0.06	0.06
VCA1018	0.04	0.04	0.06	0.05
VCA1019	0.05	0.03	0.04	0.03
VCA1019	0.05	0.05	0.04	0.03
VCA1021	0.04	0.05	0.07	0.08
VCA1024	0.06	0.06	0.07	0.05
VCA1025	0.08	0.10	0.07	0.08
VCA1026	0.05	0.06	0.05	0.05
VCA1027	0.05	0.11	0.06	0.05
VCA1028	0.04	0.04	0.06	0.02
VCA1029	0.07	0.03	0.02	0.03
VCA1030	0.06	0.05	0.05	0.05
VCA1031	0.01	0.05	0.02	0.03
VCA1032	0.08	0.10	0.08	0.10
VCA1033	0.08	0.05	0.06	0.10
VCA1034	0.06	0.06	0.06	0.04

VCA1035	0.03	0.05	0.06	0.06
VCA1037	0.01	0.06	0.04	0.05
VCA1038	0.03	0.04	0.06	0.06
VCA1039	0.02	0.02	0.04	0.03
VCA1040	0.03	0.03	0.04	0.06
VCA1041	0.06	0.07	0.06	0.05
VCA1042	0.00	0.01	0.08	0.08
VCA1043	0.03	0.03	0.04	0.05
VCA1044	0.08	0.08	0.05	0.09
VCA1045	0.07	0.07	0.05	0.04
VCA1046	0.08	0.08	0.07	0.10
VCA1047	0.07	0.08	0.08	0.10
VCA1048	0.08	0.07	0.04	0.05
VCA1049	0.03	0.03	0.04	0.04
VCA1050	0.09	0.07	0.18	0.13
VCA1051	0.05	0.08	0.05	0.06
VCA1052	0.03	0.04	0.07	0.07
VCA1053	0.04	0.04	0.04	0.06
VCA1054	0.05	0.06	0.06	0.09
VCA1055	0.03	0.02	0.04	0.06
VCA1056	0.05	0.05	-0.02	-0.01
VCA1057	0.05	0.05	0.05	0.06
VCA1058	0.04	0.03	0.08	0.10
VCA1059	0.06	0.06	0.04	0.04
VCA1060	0.07	0.08	0.06	0.09
VCA1061	0.05	0.05	0.03	0.04
VCA1062	0.04	0.05	0.06	0.08
VCA1063	0.05	0.05	0.06	0.04
VCA1064	0.05	0.04	0.04	0.05
VCA1065	0.07	0.08	0.05	0.06
VCA1065	0.08	0.05	0.05	0.05
VCA1066	0.07	0.05	0.05	0.05
VCA1067	0.07	0.07	0.03	0.02
VCA1068	-0.01	-0.02	0.08	0.07
VCA1069	0.06	0.05	0.06	0.04
VCA1070	0.03	0.03	0.09	0.08
VCA1071	0.03	0.04	0.06	0.06
VCA1072	0.06	0.05	0.01	0.03
VCA1073	0.06	0.05	0.05	0.04
VCA1074	0.06	0.07	0.14	0.16
VCA1075	0.02	0.02	0.04	0.04
VCA1076	0.04	0.04	0.06	0.06
VCA1077	0.06	0.07	0.03	0.04

VCA1078	0.03	0.03	0.04	0.03
VCA1079	0.08	0.08	0.02	0.04
VCA1080	0.03	0.03	0.06	0.05
VCA1081	0.08	0.10	0.04	0.03
VCA1083	0.61	0.61	0.54	0.58
VCA1084	0.04	0.03	0.04	0.06
VCA1085	0.06	0.06	0.05	0.04
VCA1086	0.08	0.09	0.06	0.06
VCA1087	0.08	0.09	0.06	0.07
VCA1088	0.04	0.03	0.04	0.04
VCA1089	0.02	0.05	0.05	0.05
VCA1090	0.06	0.07	0.04	0.04
VCA1091	0.04	0.06	0.05	0.06
VCA1092	0.04	0.03	0.12	0.09
VCA1093	0.06	0.05	0.05	0.06
VCA1094	0.04	0.05	0.00	-0.01
VCA1095	0.04	0.04	0.02	0.02
VCA1096	0.04	0.03	0.07	0.06
VCA1097	0.03	0.03	0.03	0.08
VCA1098	0.05	0.03	0.01	0.02
VCA1099	0.01	0.04	0.07	0.08
VCA1100	0.03	0.03	0.04	0.08
VCA1101	0.02	0.02	0.06	0.04
VCA1102	0.16	0.15	0.03	0.03
VCA1103	0.07	0.07	0.03	0.04
VCA1104	0.00	0.03	0.04	0.04
VCA1105	0.00	0.02	0.06	0.04
VCA1106	0.01	0.02	0.04	0.05
VCA1106	0.00	0.02	0.05	0.04
VCA1107	0.02	0.03	0.05	0.04
VCA1108	0.01	0.03	0.05	0.06
VCA1109	0.03	0.05	0.05	0.06
VCA1110	0.01	0.00	0.17	0.15
VCA1111	0.04	0.04	0.01	0.00
VCA1112	0.04	0.05	0.04	0.04
VCA1113	0.05	0.04	0.08	0.05
VCA1114	0.08	0.08	0.03	0.03
VCA1115	0.05	0.05	0.06	0.05
