# Supplementary material for: Systematic Identification of Cyclic-di-GMP Binding Proteins in Vibrio cholerae Reveals a Novel Class of Cyclic-di-GMP-Binding ATPases Associated with Type II Secretion Systems
Source: PLoS Pathog. 2015 Oct 27;11(10):e1005232. doi: 10.1371/journal.ppat.1005232 (PMC4624772; doi:10.1371/journal.ppat.1005232)
Supplement: S2 Table — (PDF) [file ppat.1005232.s008.pdf]

S2 Table. Strains

Strain	Description	Source
NEB T7 Express I^q	MiniF *lacI^q*(Cam^R) / *fhuA2 lacZ::T7 gene1 [lon] ompT gal sulA11 R(mcr-73::miniTn10*--Tet^S*)2 [dcm] R(zgb-210::Tn10*--Tet^S*) endA1 Δ(mcrC-mrr)114::IS10*	NEB
FY_VC_9573	WT mTn*7*-gfp, Rif^r Gm^r	This work
FY_VC_9575	Δ*mshA* mTn7-gfp, Rif^r Gm^r	This work
FY_VC_9586	Δ*mshE* mTn7-gfp, Rif^r Gm^r	This work
