# Supplementary material for: Systematic Identification of Cyclic-di-GMP Binding Proteins in Vibrio cholerae Reveals a Novel Class of Cyclic-di-GMP-Binding ATPases Associated with Type II Secretion Systems
Source: PLoS Pathog. 2015 Oct 27;11(10):e1005232. doi: 10.1371/journal.ppat.1005232 (PMC4624772; doi:10.1371/journal.ppat.1005232)
Supplement: S3 Table — (PDF) [file ppat.1005232.s009.pdf]

S3 Table. Plasmids

Plasmid	Description	Source
pVL-Blunt	pCR-Blunt derivative with restriction sites removed	{Lee, 2007 #2089}
pVL791	pET-19b derivative	{Lee, 2007 #2089}
pVL847	pET-19b derivative with an in-frame *mbp* between the His tag and the MCS	{Lee, 2007 #2089}
pVL-Blunt-MshE (VC0405)	MshE amplified with KR221 and KR228 and cloned into pVL-Blunt	This work
pVL-Blunt-PA14_29490	PA14_29490 amplified with KR203 and KR204 and cloned into pVL-Blunt	This work
pVL-Blunt-PA14_58750	PA14_58750 amplified with KR205 and KR206 and cloned into pVL-Blunt	This work
pVL-Blunt-PA14_55440	PA14_55440 amplified with KR207 and KR208 and cloned into pVL-Blunt	This work
pVL-Blunt-PA14_23990	PA14_23990 amplified with KR209 and KR210 and cloned into pVL-Blunt	This work
pVL-Blunt-PA14_68820	PA14_68820 amplified with KR211 and KR212 and cloned into pVL-Blunt	This work
pVL-Blunt-PA14_59290	PA14_59290 amplified with KR213 and KR214 and cloned into pVL-Blunt	This work
pVL-Blunt-PA14_05180	PA14_05180 amplified with KR215 and KR216 and cloned into pVL-Blunt	This work
pVL-Blunt-PA14_05190	PA14_05190 amplified with KR217 and KR218 and cloned into pVL-Blunt	This work
pVL-Blunt-PA14_59340	PA14_59340 amplified with KR219 and KR220 and cloned into pVL-Blunt	This work
pVL-Blunt-MshE-F1	MshE Fragment 1 amplified with KR221 and KR222 and cloned into pVL-Blunt	This work
pVL-Blunt-MshE-F2	MshE Fragment 2 amplified with KR221 and KR223 and cloned into pVL-Blunt	This work
pVL-Blunt-MshE-F3	MshE Fragment 3 amplified with KR221 and KR224 and cloned into pVL-Blunt	This work
pVL-Blunt-MshE-F4	MshE Fragment 4 amplified with KR225 and KR228 and cloned into pVL-Blunt	This work
pVL-Blunt-MshE-F5	MshE Fragment 5 amplified with KR226 and KR228 and cloned into pVL-Blunt	This work
pVL-Blunt-MshE-F6	MshE Fragment 6 amplified with KR227 and KR228 and cloned into pVL-Blunt	This work
pVL791-MshE $_{R9A, D12A}$	MshE$_{R9AD12A}$ is introduced into pVL791-MshE using SH60 and SH61	This work
pVL791-MshE $_{R88A, R89A}$	MshE$_{R88AR89A}$ is introduced into pVL791-MshE using SH76 and SH77	This work
pVL-Blunt-MshE $_{D108A, D111A}$	MshE$_{D108AD111A}$ is introduced into pVL-Blunt-MshE using SH64 and SH65	This work
pVL-Blunt-MshE $_{Y145A, R146A, R147A}$	MshE$_{Y145AR146AR147A}$ is introduced into pVL-Blunt-MshE using SH66 and SH67	This work
pVL-791-MshE $_{Y145A, R146A, R147A}$	MshE$_{Y145AR146AR147A}$ excised from pVL-Blunt-MshE$_{Y145AR146AR147A}$ with NdeI and BamHI and ligated into pVL791 digested with NdeI and BamHI	This work
pVL791-MshE $_{R146A, R147A}$	MshE$_{R146AR147A}$ is introduced into pVL791-MshE$_{Y145AR146AR147A}$ using SH78 and SH79	This work
pVL-Blunt-MshE $_{E191A, D192A}$	MshE$_{E191AD192A}$ is introduced into pVL-Blunt-MshE using SH68 and	This work

	SH69	
pVL791-MshE $_{R9A}$	R9A is introduced into pVL791-MshE using SH72 and SH73	This work
pVL791-MshE $_{D12A}$	D12A is introduced into pVL791-MshE$_{R9AD12A}$ using SH74 and SH75	This work
pVL-Blunt-MshE $_{Q32A}$	Q32A is introduced into pVL-Blunt-MshE using KR229 and KR230	This work
pVL-Blunt-MshE $_{E51A}$	E51A is introduced into pVL-Blunt-MshE using KR231 and KR232	This work
pVL791-MshE $_{R88A}$	R88A is introduced into pVL791-MshE$_{R88AR89A}$ using SH85 and SH86	This work
pVL791-MshE $_{R89A}$	R89A is introduced into pVL791-MshE$_{R88AR89A}$ using SH87 and SH88	This work
pVL-Blunt-MshE $_{D108A}$	D108A is introduced into pVL-Blunt-MshE using KR233 and KR234	This work
pVL791-MshE $_{D111A}$	D111A is introduced into pVL791-MshE using SH89 and SH90	This work
pVL-Blunt-MshE $_{D142A}$	D142A is introduced into pVL-Blunt-MshE using KR235 and KR236	This work
pVL-Blunt-PA14_29490-F1	PA14_29490 Fragment 1 was amplified with KR203 and SH54 and cloned into pVL-Blunt	This work
pVL-Blunt-PA14_29490-F2	PA14_29490 Fragment 2 was amplified with KR203 and SH55 and cloned into pVL-Blunt	This work
pVL-Blunt-PA14_29490-F3	PA14_29490 Fragment 3 was amplified with KR203 and SH56 and cloned into pVL-Blunt	This work
pVL-Blunt-PA14_29490-F4	PA14_29490 Fragment 4 was amplified with SH57 and KR204 and cloned into pVL-Blunt	This work
pVL-Blunt-PA14_29490-F5	PA14_29490 Fragment 5 was amplified with SH58 and KR204 and cloned into pVL-Blunt	This work
pVL-Blunt-PA14_29490-F6	PA14_29490 Fragment 6 was amplified with SH59 and KR204 and cloned into pVL-Blunt	This work
pVL791-MshE (VC0405)	MshE wild-type excised from pVL-Blunt-MshE with NdeI and BamHI and ligated into pVL791 digested with NdeI and BamHI	This work
pVL791-VC0462	VC0462 isolated from His-ORF library	This work
pVL791-VC0463	VC0463 isolated from His-ORF library	This work
pVL791-VC2424	VC2424 isolated from His-ORF library	This work
pVL791-VC0835	VC0835 isolated from His-ORF library	This work
pVL791-VC2732	VC2732 isolated from His-ORF library	This work
pVL791-PA14_29490	PA14_29490 excised from pVL-Blunt-PA14_29490 with NdeI and BamHI and ligated into pVL791 digested with NdeI and BamHI	This work
pVL791-PA14_58750	PA14_58750 excised from pVL-Blunt-PA14_58750 with AseI and BglII and ligated into pVL791 digested with NdeI and BamHI	This work
pVL791-PA14_55440	PA14_55440 excised from pVL-Blunt-PA14_55440 with NdeI and BamHI and ligated into pVL791 digested with NdeI and BamHI	This work
pVL791-PA14_23990	PA14_23990 excised from pVL-Blunt-PA14_29490 with NdeI and BamHI and ligated into pVL791 digested with NdeI and BamHI	This work
pVL791-PA14_68820	PA14_68820 excised from pVL-Blunt-PA14_68820 with NdeI and BamHI and ligated into pVL791 digested with NdeI and BamHI	This work
pVL791-PA14_59290	PA14_59290 excised from pVL-Blunt-PA14_59290 with NdeI and BglII and ligated into pVL791 digested with NdeI and BamHI	This work
pVL791-PA14_05180	PA14_05180 excised from pVL-Blunt-PA14_05180 with NdeI and BamHI and ligated into pVL791 digested with NdeI and BamHI	This work
pVL791-PA14_05190	PA14_05190 excised from pVL-Blunt-PA14_05190 with NdeI and BamHI and ligated into pVL791 digested with NdeI and BamHI	This work
pVL791-PA14_59340	PA14_59340 excised from pVL-Blunt-PA14_59340 with NdeI and XhoI and ligated into pVL791 digested with NdeI and XhoI	This work
pVL847-MshE	MshE wild-type excised from pVL-Blunt-MshE with NdeI and BamHI and ligated into pBL847 digested with NdeI and BamHI	This work
pVL847-MshE-F1	MshE Fragment 1 excised from pVL-Blunt-MshE-F1 with NdeI and BamHI and ligated into pBL847 digested with NdeI and BamHI	This work
pVL847-MshE-F2	MshE Fragment 2 excised from pVL-Blunt-MshE-F2 with NdeI and	This work

	BamHI and ligated into pBL847 digested with NdeI and BamHI	
pVL847-MshE-F3	MshE Fragment 3 excised from pVL-Blunt-MshE-F3 with NdeI and BamHI and ligated into pBL847 digested with NdeI and BamHI	This work
pVL847-MshE-F4	MshE Fragment 4 excised from pVL-Blunt-MshE-F4 with NdeI and BamHI and ligated into pBL847 digested with NdeI and BamHI	This work
pVL847-MshE-F5	MshE Fragment 5 excised from pVL-Blunt-MshE-F5 with NdeI and BamHI and ligated into pBL847 digested with NdeI and BamHI	This work
pVL847-MshE-F6	MshE Fragment 6 excised from pVL-Blunt-MshE-F6 with NdeI and BamHI and ligated into pBL847 digested with NdeI and BamHI	This work
pVL847-$MshE_{R9AD12A}$	$MshE_{R9AD12A}$ excised from pVL791-$MshE_{R9AD12A}$ with NdeI and BamHI and ligated into pVL847 digested with NdeI and BamHI	This work
pVL847-$MshE_{R88AR89A}$	$MshE_{R88AR89A}$ excised from pVL791-$MshE_{R88AR89A}$ with NdeI and BamHI and ligated into pVL847 digested with NdeI and BamHI	This work
pVL847-$MshE_{D108AD111A}$	$MshE_{D108AD111A}$ excised from pVL-Blunt-$MshE_{D108AD111A}$ with NdeI and BamHI and ligated into pVL847 digested with NdeI and BamHI	This work
pVL847-$MshE_{R146AR147A}$	$MshE_{R146AR147A}$ excised from pVL791-MshE with NdeI and BamHI and ligated into pVL847 digested with NdeI and BamHI	This work
pVL847-$MshE_{E191AD192A}$	$MshE_{E191AD192A}$ excised from pVL-Blunt-$MshE_{E191AD192A}$ with NdeI and BamHI and ligated into pVL847 digested with NdeI and BamHI	This work
pVL847-$MshE_{R9A}$	$MshE_{R9A}$ excised from pVL791-$MshE_{R9A}$ with NdeI and BamHI and ligated into pVL847 digested with NdeI and BamHI	This work
pVL847-$MshE_{D12A}$	$MshE_{D12A}$ excised from pVL791-$MshE_{D12A}$ with NdeI and BamHI and ligated into pVL847 digested with NdeI and BamHI	This work
pVL847-$MshE_{Q32A}$	$MshE_{Q32A}$ excised from pVL-Blunt-$MshE_{Q32A}$ with NdeI and BamHI and ligated into pVL847 digested with NdeI and BamHI	This work
pVL847-$MshE_{E51A}$	$MshE_{E51A}$ excised from pVL-Blunt-$MshE_{E51A}$ with NdeI and BamHI and ligated into pVL847 digested with NdeI and BamHI	This work
pVL847-$MshE_{R88A}$	$MshE_{R88A}$ excised from pVL791-$MshE_{R88A}$ with NdeI and BamHI and ligated into pVL847 digested with NdeI and BamHI	This work
pVL847-$MshE_{R89A}$	$MshE_{R89A}$ excised from pVL791-$MshE_{R89A}$ with NdeI and BamHI and ligated into pVL847 digested with NdeI and BamHI	This work
pVL847-$MshE_{D108A}$	$MshE_{D108A}$ excised from pVL-Blunt-$MshE_{D108A}$ with NdeI and BamHI and ligated into pVL847 digested with NdeI and BamHI	This work
pVL847-$MshE_{D111A}$	$MshE_{D111A}$ excised from pVL791-$MshE_{D111A}$ with NdeI and BamHI and ligated into pVL847 digested with NdeI and BamHI	This work
pVL847-$MshE_{D142A}$	$MshE_{D142A}$ excised from pVL-Blunt-$MshE_{D142A}$ with NdeI and BamHI and ligated into pVL847 digested with NdeI and BamHI	This work
pVL847-PA14_29490	PA14_29490 excised from pVL-Blunt-PA14_29490 with NdeI and BamHI and ligated into pVL847 digested with NdeI and BamHI	This work
pVL847-PA14_29490-F1	PA14_29490 Fragment 1 excised from pVL-Blunt-PA14_29490-F1 with NdeI and BamHI and ligated into pVL847 digested with NdeI and BamHI	This work
pVL847-PA14_29490-F2	PA14_29490 Fragment 2 excised from pVL-Blunt-PA14_29490-F2 with NdeI and BamHI and ligated into pVL847 digested with NdeI and BamHI	This work
pVL847-PA14_29490-F3	PA14_29490 Fragment 3 excised from pVL-Blunt-PA14_29490-F3 with NdeI and BamHI and ligated into pVL847 digested with NdeI and BamHI	This work
pVL847-PA14_29490-F4	PA14_29490 Fragment 4 excised from pVL-Blunt-PA14_29490-F4 with NdeI and BamHI and ligated into pVL847 digested with NdeI and BamHI	This work
pVL847-PA14_29490-F5	PA14_29490 Fragment 5 excised from pVL-Blunt-PA14_29490-F5 with NdeI and BamHI and ligated into pVL847 digested with NdeI and BamHI	This work
pVL847-PA14_29490-F6	PA14_29490 Fragment 6 excised from pVL-Blunt-PA14_29490-F6 with NdeI and BamHI and ligated into pVL847 digested with NdeI and BamHI	This work

pVL393	pMMB derivative with a NdeI site added to the polylinker	{Lee, 2007 #2089}
pVL393-MshE	MshE excised from pVL791-MshE with NdeI and BamHI and ligated into pVL393 digested with NdeI and BamHI	This work
pVL393-$MshE_{R9A}$	$MshE_{R9A}$ excised from pVL791-$MshE_{R9A}$ with NdeI and BamHI and ligated into pVL393 digested with NdeI and BamHI	This work
pVL393-$MshE_{D12A}$	$MshE_{D12A}$ excised from pVL791-$MshE_{D12A}$ with NdeI and BamHI and ligated into pVL393 digested with NdeI and BamHI	This work
pVL393-$MshE_{R88A, R89A}$	$MshE_{R88A, R89A}$ excised from pVL791-$MshE_{R88A, R89A}$ with NdeI and BamHI and ligated into pVL393 digested with NdeI and BamHI	This work
