# Supplementary material for: Systematic Identification of Cyclic-di-GMP Binding Proteins in Vibrio cholerae Reveals a Novel Class of Cyclic-di-GMP-Binding ATPases Associated with Type II Secretion Systems
Source: PLoS Pathog. 2015 Oct 27;11(10):e1005232. doi: 10.1371/journal.ppat.1005232 (PMC4624772; doi:10.1371/journal.ppat.1005232)
Supplement: S4 Table — (PDF) [file ppat.1005232.s010.pdf]

S4 Table. Primers

Name	Sequence (5'-3')	Use
SH54	AAGGATCCCAGGTCCAGGGAATGCTCCAA	PA14_29490 Fragment 1
SH55	AAGGATCCGGAGATCAGCTTGATCCGCGA	PA14_29490 Fragment 2
SH56	AAGGATCCCATCCCGAGCCGGTCCAGG	PA14_29490 Fragment 3
SH57	AACATATGCTCCCGGACCACTTGCG	PA14_29490 Fragment 4
SH58	AACATATGTCCGCCATCGACATCGC	PA14_29490 Fragment 5
SH59	AACATATGCTGGCCCGGCAAGGT	PA14_29490 Fragment 6
KR222	AAGGATCCAATTTCTTTGGTGCGGCGATAG	MshE Fragment 1
KR223	AAGGATCCCGCCATTAATTTTAAGCGCAGC	MshE Fragment 2
KR224	AAGGATCCAATACCTGACTCTTCGAGCTTTCTAACC	MshE Fragment 3
KR225	AACATATGATTGTCTCGTTTGCCGAGCAAT	MshE Fragment 4
KR226	AACATATGCGCTTAAAATTAATGGCGAATCTGG	MshE Fragment 5
KR227	AACATATGATTCCGCCTCATTTATTGCTGC	MshE Fragment 6
KR229	GCTTGAACAAGCGCTCAATGCCGCGAAAAATACCGGACGCCGCTTAGG	MshE$_{Q32A}$
KR230	CCTAAGCGGCGTCCGGTATTTTTCGCGGCATTGAGCGCTTGTTCAAGC	MshE$_{Q32A}$
KR231	ACCTTAATTTCGCTTGGCTTTTTAAGCGCTACCCAGTTGCTGAACTTTTTGGCG	MshE$_{E51A}$
KR232	CGCCAAAAAGTTCAGCAACTGGGTAGCGCTTAAAAAGCCAAGCGAAATTAAGGT	MshE$_{E51A}$
KR233	GATACGCTGCGCATAGCCATGAGTGCACCTGCGGATTTGTTTGCTCAAGA	MshE$_{D108A}$
KR234	TCTTGAGCAAACAAATCCGCAGGTGCACTCATGGCTATGCGCAGCGTATC	MshE$_{D108A}$
KR235	GAGAAGCAGTTGGTCGATGGCTTTGCTCGTTACTATCGCCGCACCAAAGA	MshE$_{D142A}$
KR236	TCTTTGGTGCGGCGATAGTAACGAGCAAAGCCATCGACCAACTGCTTCTC	MshE$_{D142A}$
SH60	CACCATGCCAATTAATAAACTGCGTAAAGCGCTTGGTGCATTGCTGGTTGAAGAGGGGATTGTG	MshE$_{R9A\ D12A}$
SH61	CACAATCCCCTCTTCAACCAGCAATGCACCAAGCGCTTTACGCAGTTTATTAATTGGCATGGTG	MshE$_{R9A\ D12A}$
SH64	GATACGCTGCGCATAGCCATGAGTGCACCTGCGGCATTGTTTGCTCAAGAAGCCTTGCTCA	MshE$_{D108A\ D111A}$
SH65	TGAGCAAGGCTTCTTGAGCAAACAATGCCGCAGGTGCACTCATGGCTATGCGCAGCGTATC	MshE$_{D108A\ D111A}$
SH66	AGTTGGTCGATGGCTTTGATCGTTACGCGGCCGCTACCAAAGAAATTGTCTCGTTTGCCG	MshE$_{Y145A\ R146A\ R147A}$
SH67	CGGCAAACGAGACAATTTCTTTGGTAGCGGCCGCGTAACGATCAAAGCCATCGACCAACT	MshE$_{Y145A\ R146A\ R147A}$
SH68	CGGTTGTTAAACTGATCAACTCGCTGTTTGCGGCCGCGATTCAAGTGGGAGCCTCG	MshE$_{E191A\ D192A}$
SH69	CGAGGCTCCCACTTGAATCGCGGCCGCAAACAGCGAGTTGATCAGTTTAACAACCG	MshE$_{E191A\ D192A}$
SH72	GATCTCATATGCCAATTAATAAACTGCGTAAAGCGCTTGGTGACTTGCTGGTTGAAGAG	MshE$_{R9A}$
SH73	CTCTTCAACCAGCAAGTCACCAAGCGCTTTACGCAGTTTATTAATTGGCATATGAGATC	MshE$_{R9A}$
SH74	GATCTCATATGCCAATTAATAAACTGCGTAAACGGCTTGGTGCATTGCTGGTTGAAG	MshE$_{D12A}$
SH75	CTTCAACCAGCAATGCACCAAGCCGTTTACGCAGTTTATTAATTGGCATATGAGATC	MshE$_{D12A}$
SH76	TTCCTTTACTGCCTGAAGTTCATGCCGCGGCATTGCGTGCGCTGGTGATAGG	MshE$_{R88A\ R89A}$
SH77	CCTATCACCAGCGCACGCAATGCCGCGGCATGAACTTCAGGCAGTAAAGGAA	MshE$_{R88A\ R89A}$
SH78	AGTTGGTCGATGGCTTTGATCGTTACTATGCCGCTACCAAAGAAATTGTCTCG	MshE$_{R146A\ R147A}$
SH79	CGAGACAATTTCTTTGGTAGCGGCATAGTAACGATCAAAGCCATCGACCAACT	MshE$_{R146A\ R147A}$
SH85	CGTTTGCGTGCGCTGGTGATAGG	MshE$_{R88A}$
SH86	CGCGGCATGAACTTCAGGC	MshE$_{R88A}$
SH87	CGCGCATTGCGTGCGCTGGTG	MshE$_{R89A}$
SH88	GGCATGAACTTCAGGCAGTAAAGGAA	MshE$_{R89A}$
SH89	CATTGTTTGCTCAAGAAGCCTTGCTCA	MshE$_{D111A}$
SH90	CCGCAGGATCACTCATGGCTATG	MshE$_{D111A}$
KR203	AACATATGTCCTCTGAGGCCGTTTCCC	PA14_29490 5'
KR204	AAGGATCCTTAGCCGCTGGTCACGCGAT	PA14_29490 3'

KR205	AAATTAATATGAACGACACAATTCAACTCAGCG	PA14_59750 5’
KR206	AAAGATCTTTAGTCCTTGGTCACGCGGTTG	PA14_59750 3’
KR207	AACATATGAGCCTGCTGCCCTACGC	PA14_55440 5’
KR208	AAGGATCCTCAGGCGTCCCGGGTCA	PA14_55440 3’
KR209	AACATATGATGACAGCCCCATTACCC	PA14_23990 5’
KR210	AAGGATCCTTATTCTTCCCGGGTCACGC	PA14_23990 3’
KR211	AACATATGTCCGTGTTCGCCACC	PA14_68820 5’
KR212	AAGGATCCCTACTTCTGTTCGCTCTGCGG	PA14_68820 3’
KR213	AACATATGACGAACCTTCAGATTGCCG	PA14_59290 5’
KR214	AAAGATCTTCAGCACGCCTCCTGCGAGTA	PA14_59290 3’
KR215	AACATATGGATATTACCGAGCTGCTCGC	PA14_05180 5’
KR216	AAGGATCCTCAGAAGTTTTCCGGGATCTTCG	PA14_05180 3’
KR217	AACATATGGAATTCGAAAAGCTGCTG	PA14_05190 5’
KR218	AAGGATCCTGGCCTACTGAAGACGGTTCA	PA14_05190 3’
KR219	AACATATGAGTGTGAACCCGATCATCC	PA14_59340 5’
KR220	AACTCGAGTTATCTCCATAGGCTTTGCCG	PA14_59340 3’
KR221	AACATATGCCAATTAATAAACTGCGTAAACG	MshE 5’
KR228	AAGGATCCCTACAGATAAATCGGTTCAACCAAACC	MshE 3’
